# Deciphering casual association between brain structure and liver cirrhosis: Insights from Mendelian randomization

**DOI:** 10.1097/MD.0000000000047976

**Published:** 2026-03-13

**Authors:** Xinyi Xiong, Guang Yang, Yuming Yao, Chiayen Lin

**Affiliations:** a Department of Infectious Diseases, Xiangya Hospital, Central South University, Changsha, Hunan, China; b Department of Orthopedics, Xiangya Hospital, Central South University, Changsha, Hunan, China; c Department of General Surgery, Xiangya Hospital, Central South University, Changsha, Hunan, China; d Department of Pancreatic Surgery, National Clinical Research Center for Geriatric Disorders, Xiangya Hospital, Central South University, Changsha, Hunan, China; e Department of Thyroid Surgery, Hunan Provincial Clinical Medical Research Centre for Thyroid Diseases, Changsha, Hunan, China.

**Keywords:** associations analysis, brain imaging-derived phenotypes, liver cirrhosis, Mendelian randomization

## Abstract

Liver cirrhosis exerts a significant influence on both the burden of family healthcare and public health expenditure. The causal relationship between brain imaging-derived phenotypes (BIDPs) and liver cirrhosis remains uncertain. This is a 2-sample Mendelian randomization (MR) study. Based on the genome-wide association study summary statistics of 1325 BIDPs and 2 cohort of liver cirrhosis traits. The main analyses were conducted using the inverse-variance weighted method. Moreover, weighted median, MR-Egger regression, weighted mode, simple mode method were also performed as sensitivity analyses. 41 BIDPs showed significant MR effects on liver cirrhosis in discovery cohort, 25 BIDPs showed significant MR effects in replication cohort. 20 BIDPs showed significant MR effects in both discovery cohort and replication cohort, with particular emphasis on the potential effects of 5 brain regions, the caudal anterior cingulate, rostral middle frontal, caudal middle frontal, isthmus cingulate, and superior frontal regions. There is a significant correlation between the structural characteristics of brain regions and the occurrence of liver cirrhosis, with close associations identified between the caudal anterior cingulate, rostral middle frontal, caudal middle frontal, isthmus cingulate, and superior frontal regions and liver cirrhosis. This study provides new insights into the brain-liver axis, suggesting that liver cirrhosis may be a disease regulated by the central nervous system.

## Graphical abstract

**Figure:**
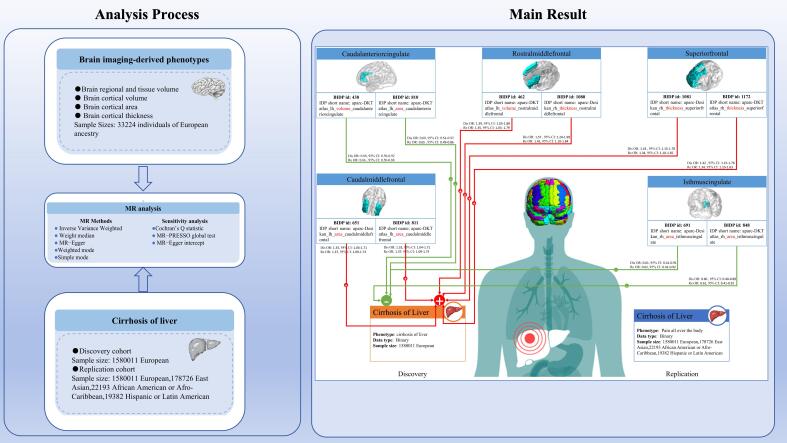

## 1. Introduction

Liver cirrhosis is a consequence of prolonged liver inflammation that triggers widespread hepatic fibrosis, leading to the substitution of normal hepatic architecture with regenerative hepatic nodules, ultimately culminating in liver failure.^[1]^ Not all patients with chronic liver inflammation progress to cirrhosis, however, the rate of progression varies from a few weeks to several decades.^[2]^ This condition is linked to significant morbidity and mortality,^[3]^ with approximately 1 million deaths globally each year attributed to liver cirrhosis.^[4]^ Cirrhosis ranks as the 11th most prevalent cause of mortality and stands as the third leading cause of death among individuals aged 45 to 64 years.^[5]^ Given the preventable nature and profound impact of such diseases, further identification of prognostic factors for their outcomes is deemed imperative.

Numerous factors have been reported to be associated with liver cirrhosis, including viral infections,^[6]^ alcohol consumption,^[7]^ genetic factors,^[8]^ autoimmune factors,^[9]^ vascular factors,^[10]^ medication use,^[11]^ and more. Additionally, the role of the gut-liver-brain axis^[12-14]^ in liver disease has received widespread attention. Such studies indicate that the interactions between the gut and liver can have a certain degree of impact on the brain function, leading to conditions like hepatic encephalopathy, Alzheimer’s disease,^[15]^ Parkinson’s disease,^[16]^ anxiety,^[17]^ depression.^[17,18]^ Recent research suggests that brain regions of the central nervous system, such as the cerebral cortex and white matter, can also have a certain degree of influence on metabolism and specific behaviors, thereby affecting liver function.^[19,20]^ However, the precise localization of these specific brain regions in relation to the liver remains unclear. Clarifying the potential connections between these specific brain regions and the liver may offer new insights for the prevention of such diseases.

Randomized controlled trials are considered the gold standard for causal inference in epidemiological studies.^[21]^ However, they are often difficult to conduct due to ethical constraints and high costs.^[22,23]^ Recent advancements in Mendelian randomization (MR) provide a robust method to mimic randomized controlled trial.^[24,25]^ It is a type of instrumental variables (IVs) analysis that has been employed to test causal hypotheses in observational data.^[26]^ This approach uses genetic variants as proxies for exposure factors, enabling random grouping and the collection of summary statistics on the associations between these variants and phenotypic outcomes in large populations.

In this study, we utilized MR to explore the causal relationships between brain imaging-derived phenotypes (BIDPs) and liver cirrhosis. Our analysis encompassed 1325 BIDPs and 2 cohort of liver cirrhosis. This could provide a scientific foundation for the development of novel preventative strategies, thereby making a positive impact on public health.

## 2. Methods and materials

### 2.1. Data source

#### 2.1.1. BIDPs

Genome-wide association study (GWAS) of BIDPs were conducted with population of 33,224 individuals of European in UK Biobank. Summary statistics for the BIDPs were obtained from the Oxford Brain Imaging Genetics (BIG40) web server (https://open.win.ox.ac.uk/ukbiobank/big40). A total of 1325 BIDPs were collected from multimodal brain imaging for further analysis. These BIDPs comprised 647 phenotypes associated with brain regional and tissue volume measured using magnetic resonance imaging (MRI), 372 phenotypes related to cortical area assessed by MRI, and 306 phenotypes related to cortical thickness also measured using MRI, all these BIDPs were corrected for age and sex as covariates.

#### 2.1.2. Liver cirrhosis

In this study, 2 cohort of liver cirrhosis were included.^[27]^ The first is discover cohort, GWAS meta-analysis were conducted based on 9 studies, comprising 15,225 cases with cirrhosis and 1564,786 controls of European ancestry (GCST90319877). The second is replication cohort, cross-ancestry fixed-effects GWAS meta-analysis were conducted based on individuals of East Asian (9.9%), African American (1.2%), Hispanic (1.0%) and European (87.9%) ancestries, totaling 18,265 cases and 17,82,047 controls (GCST90319878). GWAS summary data of these 2 cohorts were extracted from GWAS Catalog (https://www.ebi.ac.uk/gwas/).

### 2.2. MR study design

**Figure 1.**
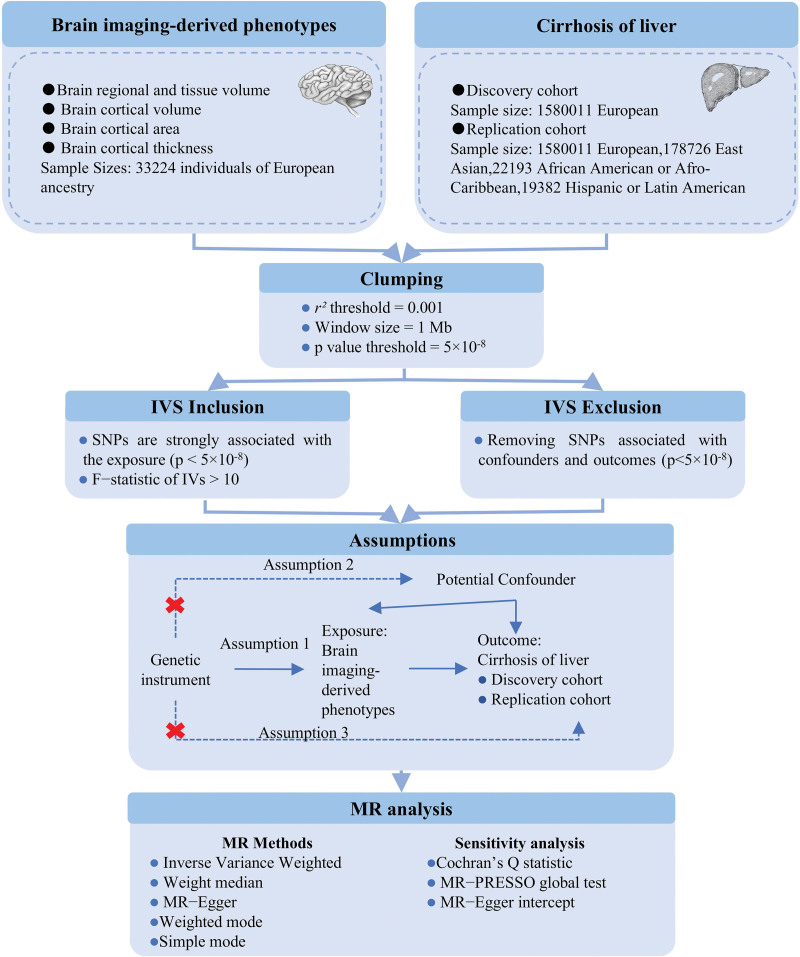
Workflow of MR. Assumption 1: the instrumental variables must be strongly associated with the exposures; Assumption 2: the instrumental variables must be independent of the potential confounders of the association between the exposure and outcome; Assumption 3: the instrumental variables should not be associated with the outcomes directly. IVS = instrumental variables, MR = Mendelian randomization.

This is a 2-sample bidirectional MR using genetic variants to mimic the effect of BIDPs on liver cirrhosis. To ensure the validity of our MR analysis, we adhered to 3 core assumptions – Assumption 1: the IVs must be strongly associated with the exposures; Assumption 2: the IVs must be independent of the potential confounders of the association between the exposure and outcome; Assumption 3: the IVs should not be associated with the outcomes directly (Fig. 1). For assumption 1, single nucleotide polymorphisms (SNPs) with *P* < 5 × 10^−8^ and *F* statistic > 10 were selected as IVs for MR analysis. In addition, a clumping process (*r*^2^ > 0.001, clumping distance = 10,000 kb) was conducted to assess the linkage disequilibrium between the included SNPs. For assumption 2, PhenoScanner^[28]^ and PhenoScanner V2^[29]^ was used to exclude SNPs associated with confounding factors (age, sex, alcohol intake, body mass index). For assumption 3, IVs that are significantly associated with the outcome phenotype (*P* < 5 × 10^−8^) were excluded.

To conduct MR estimates, the random-effects model inverse-variance weighted (IVW) method was applied as the primary statistical analysis approach.^[30]^ Random-effect IVW were conducted to reduce bias when heterogeneity exists.^[31]^ The weighted median method, MR-Egger regression, weighted mode, simple mode method were also performed as sensitivity analyses. MR-Egger intercept test was performed as an indicator of directional pleiotropy (*P* < .05 was considered statistically significant). MR-PRESSO test was also used to evaluate overall horizontal pleiotropy and detect SNP outliers. Cochran’s *Q* statistic was used to check the heterogeneity among SNPs. The leave-one-out method was employed to evaluate whether single SNP could exert bias to the IVW estimate. The R packages “TwoSampleMR” (version 0.6.4) were used to conduct MR. Benjamini–Hochberg procedure implemented in R (version 4.3.2; R Foundation for Statistical Computing, Vienna, Austria) was used to obtain adjusted p values, *P* < .05 was considered as statistically significant in genetic correlation and MR analyses.

## 3. Result

### 3.1. 41 BIDPs showed significant effect on liver cirrhosis in discovery cohort

669 BIDPs were included after IVs inclusion and exclusion, among them, 41 BIDPs showed significant MR effects on liver cirrhosis in discover cohort. 21 BIDPs were observed to exacerbate the occurrence of liver cirrhosis, with their odd radios (ORs) exceeding 1. Among them, aparc-Desikan_rh_thickness_rostralmiddlefrontal(BIDPs id: 1080) rank first, the odd ratio is 1.570 (95% CI: 1.237–1.933). The odd ratio value of aseg_lh_volume_VentralDC (BIDPs id: 202) is the smallest, at 1.175 (95% CI: 1.011–1.366). The other BIDPs are documented in Table S1, Supplemental Digital Content, http://links.lwvw.com/MD/R529 with their ORs ranging between 1.175 and 1.570. 20 BIDPs were found to exert a protective effect against the onset of liver cirrhosis, with their ORs being <1. Among them, the odd ratio value of aparc-pial_lh_area_middletemporal (BIDPs id: 730) is the smallest, at 0.599 (95% CI: 0.441–0.813).

### 3.2. 25 BIDPs showed significant effect on liver cirrhosis in replication cohort

650 BIDPs were included after IVs inclusion and exclusion, among them, 25 BIDPs showed significant MR effects on liver cirrhosis in replication cohort. 13 BIDPs were observed to exacerbate the occurrence of liver cirrhosis, with their ORs exceeding 1. Among them, aparc-a2009s_rh_volume_S-circular-insula-inf (BIDPs id: 621) rank first, the odd ratio is 1.460 (95% CI: 1.091–1.952). The odd ratio value of aparc-a2009s_lh_thickness_G-occipital-middle (BIDPs id: 1196) is the smallest, at 1.278 (95% CI: 1.018–1.605). The other BIDPs are documented in Table S2, Supplemental Digital Content, https://links.lww.com/MD/R529, with their ORs ranging between 1.278 and 1.460. 12 BIDPs were found to exert a protective effect against the onset of liver cirrhosis, with their ORs being <1. Among them, the odd ratio value of ThalamNuclei_rh_volume_VAmc (BIDPs id: 322) is the smallest, at 0.493 (95% CI: 0.284–0.855).

### 3.3. 20 BIDPs showed significant effect in both discovery cohort and replication cohort

**Figure 2.**
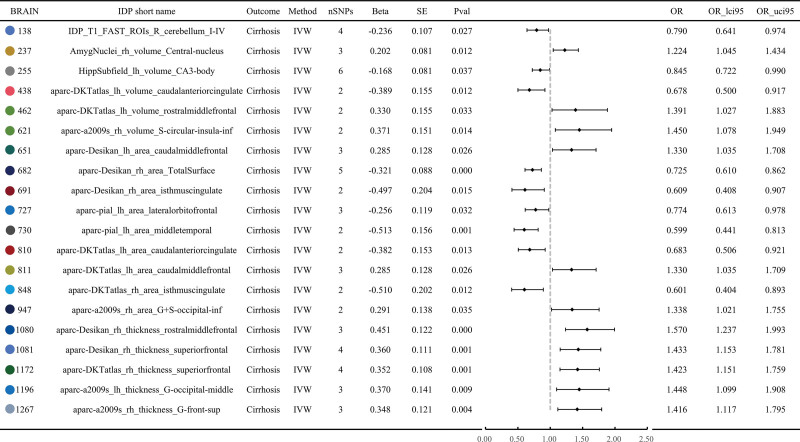
Forest plot of MR result in discovery cohort. IDP = imaging-derived phenotype, IVW = inverse-variance weighted, MR = Mendelian randomization, OR = odds ratio, SE = standard error, SNP = single nucleotide polymorphism.

**Figure 3.**
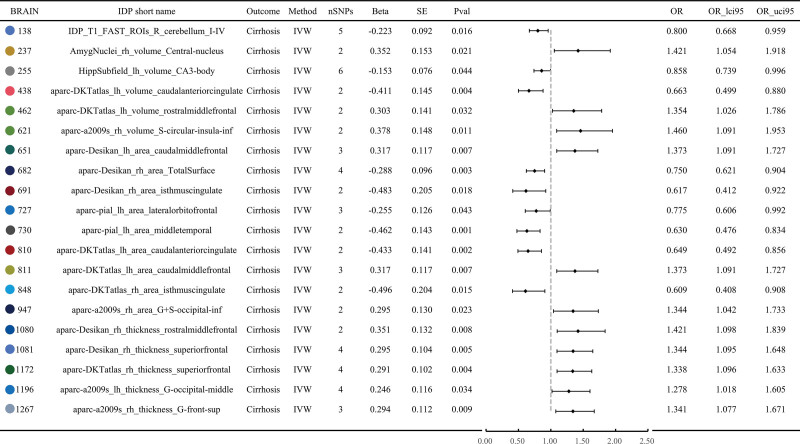
Forest plot of MR result in replication cohort. IDP = imaging-derived phenotype, IVW = inverse-variance weighted, MR = Mendelian randomization, OR = odds ratio, SE = standard error, SNP = single nucleotide polymorphism.

**Figure 4.**
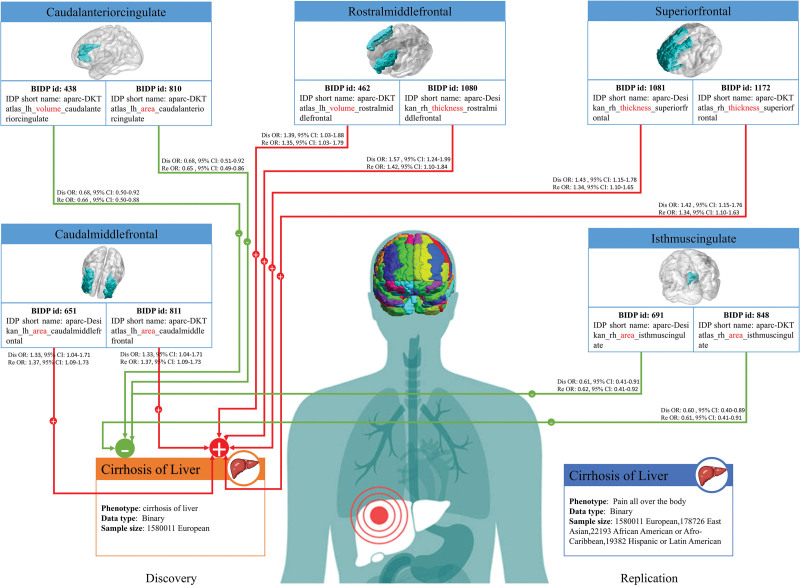
Interaction mode between brain region and liver cirrhosis. BDIP = brain imaging-derived phenotype, IDP = imaging-derived phenotype.

20 BIDPs showed significant MR effects on liver cirrhosis in both discovery cohort and replication cohort (Table S3, Supplemental Digital Content, https://links.lww.com/MD/R529), specifically IDP_T1_FAST_ROIs_R_cerebellum_I-IV(BIDPs id: 138), AmygNuclei_rh_volume_Central-nucleus (BIDPs id: 237), HippSubfield_lh_volume_CA3-body (BIDPs id:255), aparc-DKTatlas_lh_volume_caudalanteriorcingulate (BIDPs id: 438), aparc-DKTatlas_lh_volume_rostralmiddlefrontal (462, aparc-a2009s_rh_volume_S-circular-insula-inf (BIDPs id: 621), aparc-Desikan_lh_area_caudalmiddlefrontal (BIDPs id: 651), aparc-Desikan_rh_area_TotalSurface (BIDPs id: 682), aparc-Desikan_rh_area_isthmuscingulate (BIDPs id: 691), aparc-pial_lh_area_lateralorbitofrontal (BIDPs id: 727), aparc-pial_lh_area_middletemporal (BIDPs id: 730), aparc-DKTatlas_lh_area_caudalanteriorcingulate (BIDPs id: 810), aparc-DKTatlas_lh_area_caudalmiddlefrontal (BIDPs id: 811), aparc-DKTatlas_rh_area_isthmuscingulate (BIDPs id: 848), aparc-a2009s_rh_area_G+S-occipital-inf (BIDPs id: 947), aparc-Desikan_rh_thickness_rostralmiddlefrontal (BIDPs id: 1080), aparc-Desikan_rh_thickness_superiorfrontal (BIDPs id: 1081), aparc-DKTatlas_rh_thickness_superiorfrontal (BIDPs id: 1172), aparc-a2009s_lh_thickness_G-occipital-middle (BIDPs id: 1196), aparc-a2009s_rh_thickness_G-front-sup (BIDPs id: 1267). Their effects on liver fibrosis are illustrated in Figures 2 and 3. Among them, 6 BIDPs were tissue volume measured using MRI, 9 BIDPs related to cortical area assessed by MRI, 5 BIDPs related to cortical thickness also measured using MRI. In addition, certain brain regions appear twice, as detailed in Figure 4. These regions are caudal anterior cingulate, rostral middle frontal, caudal middle frontal, isthmus cingulate and superior frontal, respectively.

### 3.4. Sensitivity analysis, heterogeneity test and pleiotropy test result

In the discovery cohort, 7 BIDPs with the IDs AmygNuclei_rh_volume_Central-nucleus (BIDPs id: 237), aparc-Desikan_rh_area_TotalSurface (BIDPs id: 682), aparc-Desikan_rh_thickness_rostralmiddlefrontal (BIDPs id: 1080), aparc-Desikan_rh_thickness_superiorfrontal (BIDPs id: 1081), aparc-DKTatlas_rh_thickness_superiorfrontal (BIDPs id: 1172), aparc-a2009s_lh_thickness_G-occipital-middle (1196), aparc-a2009s_rh_thickness_G-front-sup (BIDPs id: 1267) show significance in the IVW, simple median, and weighted median models. The remaining BIDPs exhibit significance only in the IVW model. In the replication cohort, 3 BIDPs with the IDs 1081, 1172, 1196 show significance in the IVW, simple median, and weighted median models. The remaining BIDPs exhibit significance only in the IVW model (Table S4, Supplemental Digital Content, https://links.lww.com/MD/R529). In this study, for some BIDPs with only 2 IVs, the MR-Egger intercept could not be calculated (Table S5, Supplemental Digital Content, https://links.lww.com/MD/R529), and no horizontal pleiotropy was detected in the other models. Both Cochran’s *Q* test and MR-PRESSO test did not detect heterogeneity (Table S5, Supplemental Digital Content, https://links.lww.com/MD/R529).

## 4. Discussion

The liver-brain axis has been a central focus for researchers.^[12,32]^ However, previous studies have predominantly explored the influence of the liver on brain function, often overlooking the potential impact of the brain on hepatic processes. Notably, the brain may modulate liver function through the regulation of systemic metabolism or direct control over hepatic metabolic pathways. Furthermore, it can affect liver health by influencing other unhealthy behaviors. In this investigation, employing MR and utilizing MRI imaging GWAS data, we examined how structural characteristics of the brain impact the development of liver cirrhosis. Our analysis revealed 20 BIDPs linked to liver cirrhosis, with particular emphasis on the potential effects of 5 brain regions, the caudal anterior cingulate, rostral middle frontal, caudal middle frontal, isthmus cingulate, and superior frontal regions.

In this study, we observed a negative correlation between the volume and surface area of the caudal anterior cingulate region and the occurrence of liver cirrhosis (Fig. 4), with consistent findings in both the discovery and replication cohorts. The association between the caudal anterior cingulate and liver cirrhosis has not been previously reported, this brain region may impact the liver through the regulation of other lifestyle behaviors and physiological systems. Studies have indicated that the gray matter density of the caudal anterior cingulate mediates the relationship between the visceral adiposity index and binge eating score,^[19]^ both of which further burden the liver.^[19]^ Moreover, the caudal anterior cingulate has been implicated in blood pressure regulation,^[33]^ while arterial pressure has been shown to increase liver stiffness,^[34]^ hastening the progression of liver cirrhosis.

Significant positive correlations between the volume and thickness of the rostral middle frontal region and the occurrence of liver cirrhosis were concurrently observed in both the discovery and replication cohorts, a finding not previously reported in the literature. Studies have suggested that the left rostral middle frontal and cerebellar cortices may predict frontal executive performance in individuals with alcohol use disorder,^[20]^ a known risk factor for liver cirrhosis. Additionally, the rostral middle frontal region has been linked to eating behaviors. In comparison with healthy controls, individuals with eating disorders exhibit reduced gray matter volume in the left lateral orbitofrontal cortex and lower cortical thickness in the left rostral middle frontal gyrus and precuneus,^[35]^ findings that remain evident even after adjusting for body mass index.

Evidence of the association between the caudal middle frontal, isthmus cingulate, and superior frontal brain regions and liver cirrhosis has not been retrieved. The specific mechanisms through which these regions influence the occurrence of liver cirrhosis may require further research for validation. In the context of this study, by identifying these specific regions, we aim to offer new insights for interventions and prevention strategies regarding liver cirrhosis, a severe disease that significantly impacts human health.

This study has several strengths. First, it overcomes the challenges often faced by traditional randomized controlled trials by utilizing MR methods to analyze the potential associations between specific brain regions and the occurrence of liver cirrhosis. Second, the study employs dual cohorts for analysis, with MR analysis conducted on both European and East Asian populations, ensuring the universality of the research findings. However, this study also has limitations. First, it can only report the observed associations and does not allow for a detailed exploration of the specific mechanisms underlying the relationship between brain regions and liver cirrhosis. Second, due to IVs for some brain regions in the GWAS data, we were unable to accurately identify the true associations with liver cirrhosis.

## 5. Conclusion

There is a significant correlation between the structural characteristics of brain regions and the occurrence of liver cirrhosis, with close associations identified between the caudal anterior cingulate, rostral middle frontal, caudal middle frontal, isthmus cingulate, and superior frontal regions and liver cirrhosis. This study provides new insights into the brain-liver axis, suggesting that liver cirrhosis may be a disease regulated by the central nervous system.

## Acknowledgments

The authors thank the participants of all genome-wide association studies (GWAS) cohorts included in the present work and the investigators of the IEU Open GWAS project, FinnGen, UK Biobank for sharing the GWAS summary statistics. This work was supported in part by the High Performance Computing Center of Central South University.

## Author contributions

**Conceptualization:** Xinyi Xiong, Chiayen Lin.

**Data curation:** Xinyi Xiong, Guang Yang, Chiayen Lin.

**Formal analysis:** Xinyi Xiong, Guang Yang.

**Investigation:** Yuming Yao.

**Methodology:** Xinyi Xiong, Yuming Yao.

**Project administration:** Chiayen Lin.

**Supervision:** Chiayen Lin.

**Validation:** Chiayen Lin.

**Writing – original draft:** Xinyi Xiong.

**Writing – review & editing:** Chiayen Lin.

## Supplementary Material
