# Supplementary material for: Deciphering casual association between brain structure and liver cirrhosis: Insights from Mendelian randomization
Source: Medicine (Baltimore). 2026 Mar 13;105(11):e47976. doi: 10.1097/MD.0000000000047976 (PMC12991515; doi:10.1097/MD.0000000000047976)
Supplement: Supplementary file 1 [file medi-105-e47976-s001.docx]

| **Table S1: MR results in discovery cohort** | | | | | | | | | |
| --- | --- | --- | --- | --- | --- | --- | --- | --- | --- |
| BIDPs id | Outcome | Methods | nSNPs | BETA | SE | Pval | OR | OR_lci95 | OR_uci95 |
| 1 | GCST90319877 | IVW | 4 | -0.202 | 0.136 | 0.137 | 0.817 | 0.626 | 1.066 |
| 2 | GCST90319877 | IVW | 3 | -0.151 | 0.173 | 0.384 | 0.860 | 0.613 | 1.208 |
| 3 | GCST90319877 | IVW | 5 | 0.062 | 0.082 | 0.453 | 1.063 | 0.906 | 1.249 |
| 4 | GCST90319877 | IVW | 5 | 0.062 | 0.082 | 0.452 | 1.064 | 0.906 | 1.249 |
| 5 | GCST90319877 | IVW | 3 | -0.155 | 0.190 | 0.413 | 0.856 | 0.591 | 1.242 |
| 6 | GCST90319877 | IVW | 3 | 0.102 | 0.145 | 0.484 | 1.107 | 0.833 | 1.471 |
| 7 | GCST90319877 | IVW | 8 | -0.048 | 0.106 | 0.649 | 0.953 | 0.774 | 1.173 |
| 8 | GCST90319877 | IVW | 8 | -0.048 | 0.106 | 0.652 | 0.953 | 0.774 | 1.173 |
| 9 | GCST90319877 | IVW | 4 | -0.090 | 0.146 | 0.540 | 0.914 | 0.686 | 1.218 |
| 10 | GCST90319877 | IVW | 4 | -0.087 | 0.147 | 0.555 | 0.917 | 0.687 | 1.223 |
| 11 | GCST90319877 | IVW | 2 | 0.112 | 0.148 | 0.448 | 1.119 | 0.837 | 1.496 |
| 12 | GCST90319877 | IVW | 3 | -0.054 | 0.124 | 0.661 | 0.947 | 0.743 | 1.208 |
| 13 | GCST90319877 | IVW | 11 | -0.002 | 0.063 | 0.980 | 0.998 | 0.883 | 1.129 |
| 14 | GCST90319877 | IVW | 8 | -0.044 | 0.068 | 0.521 | 0.957 | 0.837 | 1.094 |
| 730 | GCST90319877 | IVW | 2 | -0.513 | 0.156 | 0.001 | 0.599 | 0.441 | 0.813 |
| 1080 | GCST90319877 | IVW | 3 | 0.451 | 0.122 | 0.000 | 1.570 | 1.237 | 1.993 |
| 17 | GCST90319877 | IVW | 5 | 0.083 | 0.174 | 0.632 | 1.087 | 0.773 | 1.529 |
| 18 | GCST90319877 | IVW | 3 | 0.082 | 0.097 | 0.400 | 1.085 | 0.897 | 1.313 |
| 19 | GCST90319877 | IVW | 4 | -0.024 | 0.119 | 0.838 | 0.976 | 0.773 | 1.232 |
| 20 | GCST90319877 | IVW | 4 | -0.094 | 0.115 | 0.412 | 0.910 | 0.726 | 1.140 |
| 23 | GCST90319877 | IVW | 2 | -0.004 | 0.157 | 0.979 | 0.996 | 0.732 | 1.356 |
| 24 | GCST90319877 | IVW | 2 | -0.204 | 0.156 | 0.191 | 0.815 | 0.600 | 1.107 |
| 25 | GCST90319877 | IVW | 11 | -0.071 | 0.113 | 0.526 | 0.931 | 0.747 | 1.161 |
| 28 | GCST90319877 | IVW | 5 | -0.191 | 0.099 | 0.052 | 0.826 | 0.681 | 1.002 |
| 29 | GCST90319877 | IVW | 8 | -0.040 | 0.079 | 0.612 | 0.961 | 0.824 | 1.121 |
| 32 | GCST90319877 | IVW | 2 | -0.237 | 0.151 | 0.117 | 0.789 | 0.587 | 1.061 |
| 33 | GCST90319877 | IVW | 3 | 0.162 | 0.166 | 0.328 | 1.176 | 0.850 | 1.627 |
| 34 | GCST90319877 | IVW | 3 | 0.161 | 0.117 | 0.168 | 1.174 | 0.934 | 1.475 |
| 35 | GCST90319877 | IVW | 2 | 0.010 | 0.122 | 0.932 | 1.010 | 0.795 | 1.285 |
| 38 | GCST90319877 | IVW | 2 | 0.024 | 0.144 | 0.868 | 1.024 | 0.773 | 1.358 |
| 40 | GCST90319877 | IVW | 2 | 0.243 | 0.195 | 0.212 | 1.276 | 0.870 | 1.870 |
| 60 | GCST90319877 | IVW | 2 | 0.075 | 0.273 | 0.783 | 1.078 | 0.632 | 1.839 |
| 61 | GCST90319877 | IVW | 3 | -0.037 | 0.134 | 0.783 | 0.964 | 0.741 | 1.253 |
| 63 | GCST90319877 | IVW | 3 | -0.135 | 0.119 | 0.256 | 0.874 | 0.692 | 1.103 |
| 66 | GCST90319877 | IVW | 3 | 0.033 | 0.160 | 0.836 | 1.034 | 0.755 | 1.415 |
| 70 | GCST90319877 | IVW | 2 | -0.094 | 0.152 | 0.536 | 0.910 | 0.676 | 1.226 |
| 72 | GCST90319877 | IVW | 14 | 0.059 | 0.050 | 0.240 | 1.060 | 0.962 | 1.170 |
| 73 | GCST90319877 | IVW | 16 | 0.089 | 0.104 | 0.393 | 1.093 | 0.891 | 1.341 |
| 78 | GCST90319877 | IVW | 5 | -0.196 | 0.169 | 0.246 | 0.822 | 0.590 | 1.145 |
| 79 | GCST90319877 | IVW | 7 | 0.108 | 0.141 | 0.444 | 1.114 | 0.845 | 1.470 |
| 80 | GCST90319877 | IVW | 4 | 0.131 | 0.108 | 0.226 | 1.140 | 0.922 | 1.409 |
| 86 | GCST90319877 | IVW | 2 | -0.017 | 0.128 | 0.896 | 0.983 | 0.766 | 1.263 |
| 90 | GCST90319877 | IVW | 5 | 0.065 | 0.214 | 0.760 | 1.068 | 0.702 | 1.624 |
| 91 | GCST90319877 | IVW | 4 | -0.175 | 0.149 | 0.241 | 0.839 | 0.626 | 1.125 |
| 93 | GCST90319877 | IVW | 3 | 0.167 | 0.215 | 0.436 | 1.182 | 0.776 | 1.800 |
| 94 | GCST90319877 | IVW | 2 | -0.143 | 0.140 | 0.308 | 0.867 | 0.659 | 1.141 |
| 95 | GCST90319877 | IVW | 3 | 0.018 | 0.131 | 0.890 | 1.018 | 0.788 | 1.315 |
| 97 | GCST90319877 | IVW | 4 | -0.067 | 0.093 | 0.473 | 0.935 | 0.779 | 1.123 |
| 100 | GCST90319877 | IVW | 3 | 0.174 | 0.331 | 0.598 | 1.191 | 0.623 | 2.277 |
| 104 | GCST90319877 | IVW | 3 | 0.179 | 0.145 | 0.216 | 1.196 | 0.901 | 1.587 |
| 106 | GCST90319877 | IVW | 3 | 0.081 | 0.124 | 0.513 | 1.085 | 0.850 | 1.383 |
| 107 | GCST90319877 | IVW | 2 | -0.064 | 0.121 | 0.595 | 0.938 | 0.740 | 1.189 |
| 111 | GCST90319877 | IVW | 2 | 0.055 | 0.163 | 0.738 | 1.056 | 0.767 | 1.455 |
| 113 | GCST90319877 | IVW | 2 | 0.139 | 0.398 | 0.728 | 1.149 | 0.526 | 2.508 |
| 116 | GCST90319877 | IVW | 3 | -0.161 | 0.116 | 0.166 | 0.851 | 0.677 | 1.069 |
| 118 | GCST90319877 | IVW | 6 | -0.060 | 0.139 | 0.666 | 0.942 | 0.718 | 1.236 |
| 119 | GCST90319877 | IVW | 2 | -0.067 | 0.132 | 0.613 | 0.935 | 0.722 | 1.212 |
| 120 | GCST90319877 | IVW | 3 | -0.157 | 0.183 | 0.391 | 0.855 | 0.598 | 1.223 |
| 121 | GCST90319877 | IVW | 6 | 0.129 | 0.103 | 0.212 | 1.137 | 0.929 | 1.392 |
| 122 | GCST90319877 | IVW | 8 | -0.040 | 0.078 | 0.606 | 0.961 | 0.825 | 1.119 |
| 123 | GCST90319877 | IVW | 9 | 0.002 | 0.070 | 0.977 | 1.002 | 0.874 | 1.149 |
| 124 | GCST90319877 | IVW | 10 | -0.046 | 0.083 | 0.580 | 0.955 | 0.811 | 1.124 |
| 1162 | GCST90319877 | IVW | 2 | 0.413 | 0.169 | 0.014 | 1.512 | 1.085 | 2.106 |
| 126 | GCST90319877 | IVW | 12 | 0.079 | 0.091 | 0.387 | 1.082 | 0.905 | 1.293 |
| 127 | GCST90319877 | IVW | 12 | 0.073 | 0.093 | 0.434 | 1.076 | 0.896 | 1.291 |
| 128 | GCST90319877 | IVW | 2 | 0.196 | 0.129 | 0.128 | 1.217 | 0.945 | 1.567 |
| 129 | GCST90319877 | IVW | 2 | 0.292 | 0.156 | 0.061 | 1.339 | 0.986 | 1.817 |
| 130 | GCST90319877 | IVW | 8 | 0.030 | 0.065 | 0.651 | 1.030 | 0.906 | 1.170 |
| 131 | GCST90319877 | IVW | 7 | -0.044 | 0.068 | 0.514 | 0.957 | 0.838 | 1.093 |
| 132 | GCST90319877 | IVW | 3 | 0.178 | 0.125 | 0.157 | 1.194 | 0.934 | 1.527 |
| 133 | GCST90319877 | IVW | 2 | -0.046 | 0.414 | 0.912 | 0.955 | 0.425 | 2.150 |
| 134 | GCST90319877 | IVW | 11 | 0.074 | 0.060 | 0.218 | 1.077 | 0.957 | 1.211 |
| 135 | GCST90319877 | IVW | 6 | 0.136 | 0.074 | 0.066 | 1.146 | 0.991 | 1.324 |
| 136 | GCST90319877 | IVW | 2 | 0.104 | 0.128 | 0.418 | 1.109 | 0.863 | 1.425 |
| 137 | GCST90319877 | IVW | 2 | 0.181 | 0.115 | 0.114 | 1.199 | 0.957 | 1.501 |
| 848 | GCST90319877 | IVW | 2 | -0.510 | 0.202 | 0.012 | 0.601 | 0.404 | 0.893 |
| 139 | GCST90319877 | IVW | 6 | 0.140 | 0.082 | 0.089 | 1.150 | 0.979 | 1.350 |
| 140 | GCST90319877 | IVW | 6 | -0.007 | 0.082 | 0.936 | 0.993 | 0.846 | 1.167 |
| 141 | GCST90319877 | IVW | 4 | -0.231 | 0.353 | 0.513 | 0.794 | 0.397 | 1.586 |
| 142 | GCST90319877 | IVW | 10 | -0.104 | 0.103 | 0.312 | 0.901 | 0.736 | 1.103 |
| 143 | GCST90319877 | IVW | 5 | 0.107 | 0.089 | 0.233 | 1.113 | 0.934 | 1.325 |
| 144 | GCST90319877 | IVW | 10 | 0.045 | 0.061 | 0.461 | 1.046 | 0.928 | 1.180 |
| 146 | GCST90319877 | IVW | 17 | 0.065 | 0.052 | 0.211 | 1.067 | 0.964 | 1.182 |
| 147 | GCST90319877 | IVW | 15 | 0.019 | 0.047 | 0.688 | 1.019 | 0.930 | 1.117 |
| 148 | GCST90319877 | IVW | 9 | -0.017 | 0.070 | 0.811 | 0.983 | 0.858 | 1.128 |
| 149 | GCST90319877 | IVW | 16 | -0.010 | 0.047 | 0.831 | 0.990 | 0.903 | 1.085 |
| 150 | GCST90319877 | IVW | 11 | 0.060 | 0.053 | 0.262 | 1.062 | 0.956 | 1.178 |
| 151 | GCST90319877 | IVW | 4 | 0.097 | 0.106 | 0.360 | 1.102 | 0.895 | 1.356 |
| 152 | GCST90319877 | IVW | 12 | 0.031 | 0.052 | 0.557 | 1.031 | 0.931 | 1.142 |
| 153 | GCST90319877 | IVW | 13 | -0.070 | 0.105 | 0.510 | 0.933 | 0.759 | 1.147 |
| 154 | GCST90319877 | IVW | 12 | 0.045 | 0.113 | 0.688 | 1.046 | 0.839 | 1.305 |
| 155 | GCST90319877 | IVW | 10 | 0.124 | 0.069 | 0.070 | 1.133 | 0.990 | 1.296 |
| 156 | GCST90319877 | IVW | 6 | -0.137 | 0.213 | 0.519 | 0.872 | 0.574 | 1.323 |
| 157 | GCST90319877 | IVW | 12 | 0.038 | 0.065 | 0.562 | 1.038 | 0.914 | 1.180 |
| 158 | GCST90319877 | IVW | 4 | 0.357 | 0.255 | 0.162 | 1.430 | 0.867 | 2.358 |
| 159 | GCST90319877 | IVW | 11 | 0.179 | 0.115 | 0.121 | 1.196 | 0.954 | 1.499 |
| 160 | GCST90319877 | IVW | 6 | -0.045 | 0.168 | 0.789 | 0.956 | 0.688 | 1.329 |
| 161 | GCST90319877 | IVW | 5 | 0.312 | 0.182 | 0.086 | 1.366 | 0.956 | 1.951 |
| 162 | GCST90319877 | IVW | 3 | 0.088 | 0.120 | 0.465 | 1.092 | 0.863 | 1.381 |
| 163 | GCST90319877 | IVW | 6 | 0.054 | 0.072 | 0.453 | 1.056 | 0.917 | 1.216 |
| 164 | GCST90319877 | IVW | 5 | 0.012 | 0.109 | 0.913 | 1.012 | 0.817 | 1.253 |
| 165 | GCST90319877 | IVW | 3 | -0.117 | 0.272 | 0.668 | 0.890 | 0.522 | 1.517 |
| 166 | GCST90319877 | IVW | 4 | -0.033 | 0.238 | 0.888 | 0.967 | 0.607 | 1.541 |
| 167 | GCST90319877 | IVW | 5 | -0.009 | 0.170 | 0.958 | 0.991 | 0.710 | 1.383 |
| 168 | GCST90319877 | IVW | 3 | 0.116 | 0.127 | 0.361 | 1.123 | 0.875 | 1.442 |
| 170 | GCST90319877 | IVW | 7 | 0.058 | 0.145 | 0.688 | 1.060 | 0.798 | 1.408 |
| 171 | GCST90319877 | IVW | 5 | -0.053 | 0.186 | 0.774 | 0.948 | 0.659 | 1.365 |
| 172 | GCST90319877 | IVW | 2 | -0.009 | 0.094 | 0.923 | 0.991 | 0.824 | 1.191 |
| 173 | GCST90319877 | IVW | 6 | 0.042 | 0.075 | 0.571 | 1.043 | 0.901 | 1.208 |
| 174 | GCST90319877 | IVW | 8 | 0.040 | 0.118 | 0.732 | 1.041 | 0.827 | 1.311 |
| 175 | GCST90319877 | IVW | 12 | -0.021 | 0.067 | 0.759 | 0.980 | 0.859 | 1.118 |
| 176 | GCST90319877 | IVW | 2 | 0.005 | 0.055 | 0.925 | 1.005 | 0.903 | 1.119 |
| 177 | GCST90319877 | IVW | 16 | -0.137 | 0.076 | 0.073 | 0.872 | 0.751 | 1.013 |
| 178 | GCST90319877 | IVW | 4 | -0.114 | 0.092 | 0.215 | 0.892 | 0.745 | 1.068 |
| 179 | GCST90319877 | IVW | 6 | 0.030 | 0.103 | 0.769 | 1.031 | 0.843 | 1.260 |
| 181 | GCST90319877 | IVW | 4 | -0.039 | 0.126 | 0.757 | 0.962 | 0.751 | 1.231 |
| 182 | GCST90319877 | IVW | 12 | -0.055 | 0.082 | 0.497 | 0.946 | 0.806 | 1.110 |
| 183 | GCST90319877 | IVW | 5 | 0.095 | 0.123 | 0.444 | 1.099 | 0.863 | 1.400 |
| 184 | GCST90319877 | IVW | 6 | -0.179 | 0.131 | 0.174 | 0.836 | 0.647 | 1.082 |
| 185 | GCST90319877 | IVW | 3 | -0.234 | 0.205 | 0.253 | 0.792 | 0.530 | 1.182 |
| 186 | GCST90319877 | IVW | 7 | 0.004 | 0.082 | 0.959 | 1.004 | 0.855 | 1.180 |
| 187 | GCST90319877 | IVW | 9 | -0.015 | 0.077 | 0.843 | 0.985 | 0.847 | 1.145 |
| 188 | GCST90319877 | IVW | 3 | 0.266 | 0.278 | 0.340 | 1.305 | 0.756 | 2.251 |
| 190 | GCST90319877 | IVW | 12 | -0.033 | 0.074 | 0.653 | 0.967 | 0.836 | 1.119 |
| 191 | GCST90319877 | IVW | 6 | -0.053 | 0.071 | 0.455 | 0.948 | 0.825 | 1.090 |
| 192 | GCST90319877 | IVW | 3 | -0.050 | 0.098 | 0.609 | 0.951 | 0.785 | 1.153 |
| 193 | GCST90319877 | IVW | 8 | 0.062 | 0.079 | 0.434 | 1.064 | 0.911 | 1.243 |
| 194 | GCST90319877 | IVW | 10 | -0.034 | 0.069 | 0.623 | 0.966 | 0.844 | 1.107 |
| 196 | GCST90319877 | IVW | 9 | -0.027 | 0.071 | 0.697 | 0.973 | 0.847 | 1.117 |
| 197 | GCST90319877 | IVW | 8 | 0.000 | 0.086 | 0.996 | 1.000 | 0.846 | 1.184 |
| 198 | GCST90319877 | IVW | 4 | 0.078 | 0.142 | 0.585 | 1.081 | 0.818 | 1.428 |
| 199 | GCST90319877 | IVW | 8 | -0.008 | 0.058 | 0.887 | 0.992 | 0.885 | 1.112 |
| 201 | GCST90319877 | IVW | 2 | -0.144 | 0.126 | 0.253 | 0.865 | 0.676 | 1.109 |
| 621 | GCST90319877 | IVW | 2 | 0.371 | 0.151 | 0.014 | 1.450 | 1.078 | 1.949 |
| 204 | GCST90319877 | IVW | 5 | 0.150 | 0.100 | 0.132 | 1.162 | 0.956 | 1.414 |
| 205 | GCST90319877 | IVW | 3 | -0.154 | 0.165 | 0.350 | 0.857 | 0.620 | 1.185 |
| 207 | GCST90319877 | IVW | 12 | -0.031 | 0.076 | 0.679 | 0.969 | 0.835 | 1.124 |
| 208 | GCST90319877 | IVW | 6 | 0.086 | 0.077 | 0.261 | 1.090 | 0.938 | 1.268 |
| 210 | GCST90319877 | IVW | 7 | 0.081 | 0.072 | 0.261 | 1.084 | 0.942 | 1.249 |
| 211 | GCST90319877 | IVW | 12 | 0.033 | 0.066 | 0.612 | 1.034 | 0.909 | 1.176 |
| 212 | GCST90319877 | IVW | 4 | -0.153 | 0.128 | 0.232 | 0.858 | 0.667 | 1.103 |
| 213 | GCST90319877 | IVW | 4 | -0.017 | 0.096 | 0.857 | 0.983 | 0.814 | 1.186 |
| 214 | GCST90319877 | IVW | 10 | 0.103 | 0.071 | 0.145 | 1.109 | 0.965 | 1.274 |
| 215 | GCST90319877 | IVW | 4 | 0.000 | 0.127 | 0.999 | 1.000 | 0.779 | 1.283 |
| 216 | GCST90319877 | IVW | 6 | -0.070 | 0.066 | 0.289 | 0.933 | 0.820 | 1.061 |
| 217 | GCST90319877 | IVW | 4 | -0.098 | 0.098 | 0.322 | 0.907 | 0.748 | 1.100 |
| 218 | GCST90319877 | IVW | 3 | -0.086 | 0.170 | 0.614 | 0.918 | 0.657 | 1.282 |
| 219 | GCST90319877 | IVW | 10 | 0.137 | 0.080 | 0.087 | 1.147 | 0.980 | 1.341 |
| 221 | GCST90319877 | IVW | 6 | 0.136 | 0.091 | 0.133 | 1.146 | 0.959 | 1.370 |
| 223 | GCST90319877 | IVW | 6 | -0.041 | 0.075 | 0.589 | 0.960 | 0.829 | 1.113 |
| 224 | GCST90319877 | IVW | 7 | -0.024 | 0.082 | 0.771 | 0.976 | 0.831 | 1.147 |
| 225 | GCST90319877 | IVW | 2 | 0.174 | 0.165 | 0.293 | 1.190 | 0.860 | 1.645 |
| 226 | GCST90319877 | IVW | 3 | -0.045 | 0.117 | 0.697 | 0.956 | 0.760 | 1.201 |
| 228 | GCST90319877 | IVW | 2 | 0.101 | 0.194 | 0.601 | 1.107 | 0.757 | 1.619 |
| 229 | GCST90319877 | IVW | 2 | 0.122 | 0.170 | 0.471 | 1.130 | 0.810 | 1.576 |
| 230 | GCST90319877 | IVW | 2 | 0.094 | 0.150 | 0.530 | 1.099 | 0.819 | 1.475 |
| 231 | GCST90319877 | IVW | 3 | 0.012 | 0.105 | 0.910 | 1.012 | 0.824 | 1.243 |
| 232 | GCST90319877 | IVW | 5 | -0.005 | 0.093 | 0.957 | 0.995 | 0.830 | 1.193 |
| 233 | GCST90319877 | IVW | 10 | 0.040 | 0.065 | 0.541 | 1.041 | 0.916 | 1.183 |
| 234 | GCST90319877 | IVW | 5 | -0.061 | 0.160 | 0.703 | 0.941 | 0.687 | 1.288 |
| 1196 | GCST90319877 | IVW | 3 | 0.370 | 0.141 | 0.009 | 1.448 | 1.099 | 1.908 |
| 236 | GCST90319877 | IVW | 3 | -0.066 | 0.191 | 0.730 | 0.936 | 0.644 | 1.361 |
| 1081 | GCST90319877 | IVW | 4 | 0.360 | 0.111 | 0.001 | 1.433 | 1.153 | 1.781 |
| 1172 | GCST90319877 | IVW | 4 | 0.352 | 0.108 | 0.001 | 1.423 | 1.151 | 1.759 |
| 1267 | GCST90319877 | IVW | 3 | 0.348 | 0.121 | 0.004 | 1.416 | 1.117 | 1.795 |
| 241 | GCST90319877 | IVW | 3 | -0.012 | 0.114 | 0.915 | 0.988 | 0.789 | 1.236 |
| 242 | GCST90319877 | IVW | 5 | 0.027 | 0.101 | 0.790 | 1.027 | 0.842 | 1.253 |
| 243 | GCST90319877 | IVW | 9 | -0.033 | 0.065 | 0.613 | 0.968 | 0.853 | 1.099 |
| 244 | GCST90319877 | IVW | 7 | -0.046 | 0.071 | 0.518 | 0.955 | 0.832 | 1.097 |
| 245 | GCST90319877 | IVW | 5 | -0.092 | 0.103 | 0.373 | 0.912 | 0.745 | 1.116 |
| 246 | GCST90319877 | IVW | 5 | 0.029 | 0.106 | 0.784 | 1.029 | 0.836 | 1.267 |
| 247 | GCST90319877 | IVW | 2 | 0.018 | 0.258 | 0.945 | 1.018 | 0.614 | 1.688 |
| 249 | GCST90319877 | IVW | 7 | -0.032 | 0.066 | 0.626 | 0.968 | 0.851 | 1.102 |
| 250 | GCST90319877 | IVW | 5 | 0.001 | 0.092 | 0.991 | 1.001 | 0.836 | 1.199 |
| 252 | GCST90319877 | IVW | 3 | -0.050 | 0.110 | 0.646 | 0.951 | 0.767 | 1.179 |
| 253 | GCST90319877 | IVW | 3 | -0.039 | 0.133 | 0.768 | 0.962 | 0.741 | 1.247 |
| 254 | GCST90319877 | IVW | 6 | 0.011 | 0.069 | 0.871 | 1.011 | 0.883 | 1.158 |
| 691 | GCST90319877 | IVW | 2 | -0.497 | 0.204 | 0.015 | 0.609 | 0.408 | 0.907 |
| 256 | GCST90319877 | IVW | 5 | -0.049 | 0.086 | 0.572 | 0.953 | 0.805 | 1.127 |
| 257 | GCST90319877 | IVW | 5 | -0.104 | 0.072 | 0.149 | 0.901 | 0.782 | 1.038 |
| 258 | GCST90319877 | IVW | 7 | -0.033 | 0.073 | 0.653 | 0.968 | 0.838 | 1.117 |
| 260 | GCST90319877 | IVW | 4 | 0.001 | 0.149 | 0.994 | 1.001 | 0.748 | 1.339 |
| 261 | GCST90319877 | IVW | 3 | 0.001 | 0.128 | 0.994 | 1.001 | 0.779 | 1.286 |
| 262 | GCST90319877 | IVW | 10 | -0.025 | 0.058 | 0.671 | 0.975 | 0.870 | 1.094 |
| 263 | GCST90319877 | IVW | 9 | -0.041 | 0.056 | 0.466 | 0.960 | 0.860 | 1.072 |
| 264 | GCST90319877 | IVW | 8 | -0.014 | 0.056 | 0.804 | 0.986 | 0.883 | 1.101 |
| 265 | GCST90319877 | IVW | 10 | 0.000 | 0.054 | 0.994 | 1.000 | 0.900 | 1.112 |
| 266 | GCST90319877 | IVW | 9 | -0.023 | 0.061 | 0.710 | 0.977 | 0.866 | 1.103 |
| 267 | GCST90319877 | IVW | 6 | -0.069 | 0.104 | 0.507 | 0.933 | 0.762 | 1.144 |
| 268 | GCST90319877 | IVW | 6 | 0.072 | 0.103 | 0.482 | 1.075 | 0.879 | 1.315 |
| 271 | GCST90319877 | IVW | 5 | -0.029 | 0.095 | 0.763 | 0.972 | 0.806 | 1.171 |
| 272 | GCST90319877 | IVW | 2 | -0.008 | 0.144 | 0.953 | 0.992 | 0.748 | 1.314 |
| 274 | GCST90319877 | IVW | 5 | -0.084 | 0.085 | 0.324 | 0.919 | 0.778 | 1.087 |
| 276 | GCST90319877 | IVW | 6 | -0.031 | 0.075 | 0.681 | 0.970 | 0.837 | 1.123 |
| 711 | GCST90319877 | IVW | 2 | -0.445 | 0.157 | 0.005 | 0.641 | 0.471 | 0.871 |
| 278 | GCST90319877 | IVW | 7 | 0.057 | 0.087 | 0.516 | 1.058 | 0.892 | 1.256 |
| 279 | GCST90319877 | IVW | 7 | -0.030 | 0.073 | 0.682 | 0.970 | 0.841 | 1.120 |
| 280 | GCST90319877 | IVW | 7 | -0.018 | 0.079 | 0.822 | 0.982 | 0.841 | 1.148 |
| 282 | GCST90319877 | IVW | 4 | -0.065 | 0.085 | 0.448 | 0.937 | 0.793 | 1.108 |
| 284 | GCST90319877 | IVW | 8 | 0.015 | 0.063 | 0.819 | 1.015 | 0.896 | 1.149 |
| 285 | GCST90319877 | IVW | 6 | 0.044 | 0.083 | 0.597 | 1.045 | 0.888 | 1.229 |
| 286 | GCST90319877 | IVW | 7 | -0.041 | 0.058 | 0.474 | 0.960 | 0.857 | 1.074 |
| 289 | GCST90319877 | IVW | 3 | 0.012 | 0.165 | 0.943 | 1.012 | 0.733 | 1.398 |
| 290 | GCST90319877 | IVW | 2 | -0.130 | 0.147 | 0.376 | 0.878 | 0.659 | 1.171 |
| 291 | GCST90319877 | IVW | 2 | -0.114 | 0.193 | 0.556 | 0.892 | 0.611 | 1.304 |
| 292 | GCST90319877 | IVW | 2 | 0.015 | 0.167 | 0.930 | 1.015 | 0.731 | 1.409 |
| 293 | GCST90319877 | IVW | 7 | 0.056 | 0.098 | 0.568 | 1.058 | 0.872 | 1.282 |
| 294 | GCST90319877 | IVW | 5 | 0.082 | 0.103 | 0.423 | 1.086 | 0.888 | 1.328 |
| 295 | GCST90319877 | IVW | 3 | -0.230 | 0.133 | 0.084 | 0.794 | 0.612 | 1.032 |
| 296 | GCST90319877 | IVW | 3 | 0.151 | 0.124 | 0.225 | 1.162 | 0.911 | 1.483 |
| 297 | GCST90319877 | IVW | 5 | 0.223 | 0.138 | 0.105 | 1.250 | 0.954 | 1.637 |
| 677 | GCST90319877 | IVW | 2 | 0.335 | 0.155 | 0.030 | 1.398 | 1.033 | 1.893 |
| 300 | GCST90319877 | IVW | 3 | -0.031 | 0.119 | 0.798 | 0.970 | 0.767 | 1.226 |
| 302 | GCST90319877 | IVW | 3 | -0.165 | 0.140 | 0.240 | 0.848 | 0.644 | 1.116 |
| 303 | GCST90319877 | IVW | 3 | -0.309 | 0.263 | 0.238 | 0.734 | 0.439 | 1.228 |
| 304 | GCST90319877 | IVW | 3 | -0.019 | 0.135 | 0.886 | 0.981 | 0.753 | 1.277 |
| 306 | GCST90319877 | IVW | 2 | 0.035 | 0.187 | 0.851 | 1.036 | 0.718 | 1.494 |
| 307 | GCST90319877 | IVW | 2 | 0.067 | 0.374 | 0.858 | 1.069 | 0.514 | 2.223 |
| 308 | GCST90319877 | IVW | 3 | -0.002 | 0.155 | 0.989 | 0.998 | 0.736 | 1.353 |
| 309 | GCST90319877 | IVW | 5 | -0.062 | 0.104 | 0.550 | 0.940 | 0.767 | 1.152 |
| 310 | GCST90319877 | IVW | 2 | 0.073 | 0.144 | 0.612 | 1.075 | 0.812 | 1.425 |
| 313 | GCST90319877 | IVW | 4 | -0.186 | 0.107 | 0.081 | 0.830 | 0.673 | 1.023 |
| 315 | GCST90319877 | IVW | 3 | -0.171 | 0.107 | 0.110 | 0.843 | 0.684 | 1.040 |
| 316 | GCST90319877 | IVW | 2 | 0.072 | 0.160 | 0.652 | 1.075 | 0.786 | 1.471 |
| 317 | GCST90319877 | IVW | 6 | 0.065 | 0.121 | 0.589 | 1.067 | 0.842 | 1.352 |
| 318 | GCST90319877 | IVW | 4 | 0.080 | 0.105 | 0.444 | 1.083 | 0.883 | 1.330 |
| 320 | GCST90319877 | IVW | 3 | 0.194 | 0.126 | 0.123 | 1.215 | 0.949 | 1.555 |
| 321 | GCST90319877 | IVW | 6 | 0.161 | 0.160 | 0.316 | 1.174 | 0.858 | 1.608 |
| 322 | GCST90319877 | IVW | 3 | -0.267 | 0.375 | 0.477 | 0.766 | 0.367 | 1.597 |
| 324 | GCST90319877 | IVW | 4 | -0.005 | 0.223 | 0.982 | 0.995 | 0.643 | 1.541 |
| 325 | GCST90319877 | IVW | 2 | -0.142 | 0.257 | 0.580 | 0.868 | 0.525 | 1.435 |
| 327 | GCST90319877 | IVW | 3 | 0.220 | 0.158 | 0.164 | 1.247 | 0.914 | 1.701 |
| 330 | GCST90319877 | IVW | 2 | 0.018 | 0.150 | 0.902 | 1.019 | 0.760 | 1.366 |
| 331 | GCST90319877 | IVW | 3 | -0.138 | 0.123 | 0.262 | 0.871 | 0.684 | 1.109 |
| 332 | GCST90319877 | IVW | 2 | -0.075 | 0.252 | 0.766 | 0.928 | 0.566 | 1.521 |
| 333 | GCST90319877 | IVW | 2 | -0.085 | 0.210 | 0.687 | 0.919 | 0.609 | 1.387 |
| 334 | GCST90319877 | IVW | 7 | 0.048 | 0.087 | 0.578 | 1.049 | 0.885 | 1.243 |
| 335 | GCST90319877 | IVW | 3 | -0.072 | 0.126 | 0.566 | 0.930 | 0.727 | 1.190 |
| 336 | GCST90319877 | IVW | 5 | 0.121 | 0.114 | 0.291 | 1.128 | 0.902 | 1.412 |
| 337 | GCST90319877 | IVW | 3 | 0.076 | 0.113 | 0.499 | 1.079 | 0.865 | 1.347 |
| 338 | GCST90319877 | IVW | 2 | -0.014 | 0.142 | 0.919 | 0.986 | 0.746 | 1.303 |
| 339 | GCST90319877 | IVW | 9 | 0.027 | 0.065 | 0.681 | 1.027 | 0.904 | 1.168 |
| 340 | GCST90319877 | IVW | 23 | -0.073 | 0.054 | 0.178 | 0.930 | 0.836 | 1.034 |
| 341 | GCST90319877 | IVW | 3 | 0.259 | 0.147 | 0.079 | 1.295 | 0.970 | 1.729 |
| 342 | GCST90319877 | IVW | 7 | 0.073 | 0.102 | 0.476 | 1.075 | 0.881 | 1.312 |
| 343 | GCST90319877 | IVW | 20 | -0.058 | 0.067 | 0.385 | 0.944 | 0.828 | 1.076 |
| 346 | GCST90319877 | IVW | 2 | 0.219 | 0.302 | 0.468 | 1.245 | 0.689 | 2.249 |
| 347 | GCST90319877 | IVW | 12 | -0.027 | 0.049 | 0.582 | 0.973 | 0.884 | 1.072 |
| 353 | GCST90319877 | IVW | 3 | -0.068 | 0.122 | 0.576 | 0.934 | 0.735 | 1.186 |
| 354 | GCST90319877 | IVW | 2 | 0.035 | 0.168 | 0.836 | 1.035 | 0.745 | 1.439 |
| 355 | GCST90319877 | IVW | 8 | -0.116 | 0.085 | 0.170 | 0.890 | 0.754 | 1.051 |
| 360 | GCST90319877 | IVW | 2 | 0.019 | 0.127 | 0.884 | 1.019 | 0.794 | 1.308 |
| 361 | GCST90319877 | IVW | 2 | -0.235 | 0.221 | 0.287 | 0.790 | 0.513 | 1.219 |
| 362 | GCST90319877 | IVW | 4 | 0.097 | 0.138 | 0.483 | 1.102 | 0.841 | 1.444 |
| 363 | GCST90319877 | IVW | 14 | -0.065 | 0.055 | 0.239 | 0.937 | 0.840 | 1.044 |
| 367 | GCST90319877 | IVW | 3 | -0.109 | 0.114 | 0.339 | 0.897 | 0.718 | 1.121 |
| 371 | GCST90319877 | IVW | 4 | 0.053 | 0.087 | 0.542 | 1.055 | 0.889 | 1.251 |
| 373 | GCST90319877 | IVW | 2 | -0.086 | 0.120 | 0.471 | 0.917 | 0.726 | 1.160 |
| 380 | GCST90319877 | IVW | 7 | 0.001 | 0.064 | 0.987 | 1.001 | 0.883 | 1.135 |
| 381 | GCST90319877 | IVW | 2 | -0.264 | 0.189 | 0.161 | 0.768 | 0.530 | 1.111 |
| 388 | GCST90319877 | IVW | 6 | 0.101 | 0.099 | 0.307 | 1.106 | 0.911 | 1.343 |
| 391 | GCST90319877 | IVW | 2 | 0.087 | 0.147 | 0.556 | 1.091 | 0.817 | 1.456 |
| 394 | GCST90319877 | IVW | 2 | -0.240 | 0.221 | 0.276 | 0.786 | 0.510 | 1.212 |
| 395 | GCST90319877 | IVW | 3 | 0.066 | 0.178 | 0.712 | 1.068 | 0.753 | 1.515 |
| 396 | GCST90319877 | IVW | 14 | 0.087 | 0.061 | 0.155 | 1.091 | 0.968 | 1.229 |
| 397 | GCST90319877 | IVW | 2 | 0.048 | 0.065 | 0.458 | 1.049 | 0.924 | 1.190 |
| 399 | GCST90319877 | IVW | 2 | 0.085 | 0.082 | 0.296 | 1.089 | 0.928 | 1.278 |
| 400 | GCST90319877 | IVW | 4 | 0.156 | 0.095 | 0.100 | 1.169 | 0.970 | 1.409 |
| 402 | GCST90319877 | IVW | 2 | 0.154 | 0.133 | 0.247 | 1.166 | 0.899 | 1.513 |
| 404 | GCST90319877 | IVW | 4 | -0.082 | 0.096 | 0.393 | 0.921 | 0.762 | 1.113 |
| 406 | GCST90319877 | IVW | 3 | -0.009 | 0.080 | 0.915 | 0.991 | 0.847 | 1.161 |
| 410 | GCST90319877 | IVW | 2 | 0.042 | 0.063 | 0.507 | 1.043 | 0.921 | 1.180 |
| 412 | GCST90319877 | IVW | 3 | 0.042 | 0.090 | 0.636 | 1.043 | 0.875 | 1.244 |
| 413 | GCST90319877 | IVW | 2 | 0.044 | 0.052 | 0.400 | 1.045 | 0.944 | 1.156 |
| 418 | GCST90319877 | IVW | 4 | 0.139 | 0.086 | 0.106 | 1.150 | 0.971 | 1.361 |
| 419 | GCST90319877 | IVW | 11 | -0.064 | 0.065 | 0.323 | 0.938 | 0.826 | 1.065 |
| 420 | GCST90319877 | IVW | 8 | -0.014 | 0.088 | 0.876 | 0.986 | 0.831 | 1.171 |
| 421 | GCST90319877 | IVW | 4 | 0.012 | 0.028 | 0.675 | 1.012 | 0.958 | 1.069 |
| 423 | GCST90319877 | IVW | 2 | -0.227 | 0.144 | 0.113 | 0.797 | 0.601 | 1.055 |
| 424 | GCST90319877 | IVW | 2 | 0.034 | 0.074 | 0.642 | 1.035 | 0.895 | 1.197 |
| 425 | GCST90319877 | IVW | 3 | 0.016 | 0.107 | 0.880 | 1.016 | 0.823 | 1.254 |
| 429 | GCST90319877 | IVW | 2 | 0.104 | 0.089 | 0.240 | 1.110 | 0.932 | 1.322 |
| 430 | GCST90319877 | IVW | 3 | 0.189 | 0.117 | 0.107 | 1.208 | 0.960 | 1.520 |
| 432 | GCST90319877 | IVW | 3 | 0.050 | 0.184 | 0.784 | 1.052 | 0.733 | 1.509 |
| 433 | GCST90319877 | IVW | 9 | 0.122 | 0.065 | 0.062 | 1.130 | 0.994 | 1.284 |
| 434 | GCST90319877 | IVW | 9 | 0.026 | 0.062 | 0.678 | 1.026 | 0.908 | 1.159 |
| 435 | GCST90319877 | IVW | 3 | 0.079 | 0.114 | 0.491 | 1.082 | 0.865 | 1.353 |
| 437 | GCST90319877 | IVW | 3 | -0.009 | 0.143 | 0.951 | 0.991 | 0.748 | 1.313 |
| 438 | GCST90319877 | IVW | 2 | -0.389 | 0.155 | 0.012 | 0.678 | 0.500 | 0.917 |
| 439 | GCST90319877 | IVW | 2 | -0.286 | 0.176 | 0.104 | 0.751 | 0.532 | 1.061 |
| 440 | GCST90319877 | IVW | 10 | 0.018 | 0.057 | 0.753 | 1.018 | 0.911 | 1.137 |
| 446 | GCST90319877 | IVW | 2 | -0.037 | 0.176 | 0.832 | 0.963 | 0.683 | 1.360 |
| 447 | GCST90319877 | IVW | 4 | 0.066 | 0.098 | 0.501 | 1.069 | 0.881 | 1.296 |
| 448 | GCST90319877 | IVW | 8 | -0.107 | 0.086 | 0.212 | 0.898 | 0.759 | 1.063 |
| 453 | GCST90319877 | IVW | 2 | 0.015 | 0.135 | 0.912 | 1.015 | 0.780 | 1.321 |
| 454 | GCST90319877 | IVW | 2 | -0.068 | 0.223 | 0.760 | 0.934 | 0.603 | 1.446 |
| 455 | GCST90319877 | IVW | 5 | 0.114 | 0.084 | 0.172 | 1.121 | 0.951 | 1.322 |
| 456 | GCST90319877 | IVW | 15 | -0.079 | 0.053 | 0.136 | 0.924 | 0.834 | 1.025 |
| 457 | GCST90319877 | IVW | 2 | 0.057 | 0.064 | 0.373 | 1.059 | 0.933 | 1.202 |
| 460 | GCST90319877 | IVW | 4 | -0.121 | 0.098 | 0.218 | 0.886 | 0.732 | 1.074 |
| 462 | GCST90319877 | IVW | 2 | 0.330 | 0.155 | 0.033 | 1.391 | 1.027 | 1.883 |
| 464 | GCST90319877 | IVW | 4 | 0.046 | 0.085 | 0.588 | 1.047 | 0.886 | 1.238 |
| 466 | GCST90319877 | IVW | 2 | -0.084 | 0.114 | 0.462 | 0.920 | 0.735 | 1.150 |
| 468 | GCST90319877 | IVW | 5 | -0.018 | 0.120 | 0.882 | 0.982 | 0.776 | 1.244 |
| 471 | GCST90319877 | IVW | 8 | -0.040 | 0.064 | 0.532 | 0.961 | 0.848 | 1.089 |
| 472 | GCST90319877 | IVW | 2 | -0.259 | 0.185 | 0.162 | 0.772 | 0.537 | 1.109 |
| 478 | GCST90319877 | IVW | 2 | 0.040 | 0.189 | 0.830 | 1.041 | 0.720 | 1.507 |
| 479 | GCST90319877 | IVW | 6 | 0.102 | 0.099 | 0.302 | 1.107 | 0.912 | 1.344 |
| 480 | GCST90319877 | IVW | 2 | 0.029 | 0.161 | 0.856 | 1.030 | 0.751 | 1.411 |
| 485 | GCST90319877 | IVW | 3 | 0.033 | 0.112 | 0.770 | 1.033 | 0.829 | 1.288 |
| 486 | GCST90319877 | IVW | 3 | 0.177 | 0.145 | 0.223 | 1.193 | 0.898 | 1.585 |
| 487 | GCST90319877 | IVW | 13 | 0.092 | 0.064 | 0.152 | 1.096 | 0.967 | 1.243 |
| 488 | GCST90319877 | IVW | 2 | 0.051 | 0.069 | 0.465 | 1.052 | 0.918 | 1.205 |
| 490 | GCST90319877 | IVW | 2 | 0.083 | 0.081 | 0.310 | 1.086 | 0.926 | 1.274 |
| 491 | GCST90319877 | IVW | 4 | 0.151 | 0.094 | 0.107 | 1.163 | 0.968 | 1.398 |
| 493 | GCST90319877 | IVW | 4 | 0.005 | 0.122 | 0.965 | 1.005 | 0.792 | 1.277 |
| 495 | GCST90319877 | IVW | 5 | 0.086 | 0.089 | 0.334 | 1.089 | 0.916 | 1.296 |
| 497 | GCST90319877 | IVW | 3 | -0.005 | 0.080 | 0.954 | 0.995 | 0.851 | 1.165 |
| 499 | GCST90319877 | IVW | 6 | 0.100 | 0.084 | 0.230 | 1.106 | 0.938 | 1.303 |
| 509 | GCST90319877 | IVW | 4 | 0.007 | 0.098 | 0.942 | 1.007 | 0.831 | 1.221 |
| 510 | GCST90319877 | IVW | 11 | -0.095 | 0.054 | 0.078 | 0.910 | 0.819 | 1.011 |
| 513 | GCST90319877 | IVW | 3 | 0.185 | 0.125 | 0.138 | 1.204 | 0.942 | 1.538 |
| 519 | GCST90319877 | IVW | 2 | -0.245 | 0.144 | 0.089 | 0.783 | 0.590 | 1.038 |
| 521 | GCST90319877 | IVW | 5 | -0.073 | 0.137 | 0.596 | 0.930 | 0.711 | 1.216 |
| 522 | GCST90319877 | IVW | 2 | -0.237 | 0.135 | 0.079 | 0.789 | 0.606 | 1.028 |
| 525 | GCST90319877 | IVW | 2 | -0.080 | 0.118 | 0.494 | 0.923 | 0.733 | 1.162 |
| 526 | GCST90319877 | IVW | 4 | 0.057 | 0.101 | 0.571 | 1.059 | 0.869 | 1.289 |
| 527 | GCST90319877 | IVW | 2 | 0.072 | 0.098 | 0.462 | 1.075 | 0.887 | 1.302 |
| 529 | GCST90319877 | IVW | 5 | -0.132 | 0.088 | 0.132 | 0.876 | 0.738 | 1.041 |
| 540 | GCST90319877 | IVW | 3 | -0.103 | 0.230 | 0.654 | 0.902 | 0.575 | 1.416 |
| 542 | GCST90319877 | IVW | 2 | 0.157 | 0.151 | 0.297 | 1.170 | 0.871 | 1.572 |
| 543 | GCST90319877 | IVW | 12 | -0.009 | 0.088 | 0.914 | 0.991 | 0.834 | 1.176 |
| 548 | GCST90319877 | IVW | 4 | 0.144 | 0.110 | 0.188 | 1.155 | 0.932 | 1.433 |
| 551 | GCST90319877 | IVW | 2 | 0.237 | 0.145 | 0.102 | 1.268 | 0.954 | 1.685 |
| 556 | GCST90319877 | IVW | 2 | 0.027 | 0.221 | 0.903 | 1.027 | 0.666 | 1.585 |
| 560 | GCST90319877 | IVW | 2 | -0.004 | 0.153 | 0.977 | 0.996 | 0.737 | 1.344 |
| 561 | GCST90319877 | IVW | 2 | 0.121 | 0.162 | 0.455 | 1.129 | 0.821 | 1.551 |
| 563 | GCST90319877 | IVW | 2 | -0.117 | 0.154 | 0.448 | 0.890 | 0.658 | 1.203 |
| 564 | GCST90319877 | IVW | 5 | 0.126 | 0.116 | 0.277 | 1.135 | 0.903 | 1.426 |
| 566 | GCST90319877 | IVW | 4 | 0.049 | 0.097 | 0.612 | 1.050 | 0.869 | 1.270 |
| 570 | GCST90319877 | IVW | 2 | -0.079 | 0.097 | 0.417 | 0.924 | 0.765 | 1.117 |
| 572 | GCST90319877 | IVW | 3 | -0.209 | 0.131 | 0.112 | 0.812 | 0.628 | 1.050 |
| 583 | GCST90319877 | IVW | 4 | -0.198 | 0.151 | 0.189 | 0.820 | 0.610 | 1.102 |
| 584 | GCST90319877 | IVW | 8 | 0.008 | 0.065 | 0.898 | 1.008 | 0.888 | 1.146 |
| 595 | GCST90319877 | IVW | 6 | -0.002 | 0.113 | 0.986 | 0.998 | 0.800 | 1.244 |
| 598 | GCST90319877 | IVW | 2 | -0.034 | 0.256 | 0.895 | 0.967 | 0.586 | 1.596 |
| 599 | GCST90319877 | IVW | 4 | -0.004 | 0.083 | 0.961 | 0.996 | 0.846 | 1.172 |
| 600 | GCST90319877 | IVW | 3 | 0.026 | 0.127 | 0.834 | 1.027 | 0.801 | 1.316 |
| 601 | GCST90319877 | IVW | 2 | 0.057 | 0.063 | 0.364 | 1.059 | 0.936 | 1.199 |
| 603 | GCST90319877 | IVW | 3 | 0.128 | 0.102 | 0.210 | 1.136 | 0.931 | 1.388 |
| 615 | GCST90319877 | IVW | 4 | 0.122 | 0.112 | 0.275 | 1.130 | 0.907 | 1.407 |
| 617 | GCST90319877 | IVW | 14 | 0.047 | 0.060 | 0.428 | 1.049 | 0.933 | 1.179 |
| 618 | GCST90319877 | IVW | 2 | 0.062 | 0.056 | 0.273 | 1.064 | 0.952 | 1.188 |
| 620 | GCST90319877 | IVW | 2 | -0.077 | 0.148 | 0.602 | 0.926 | 0.693 | 1.236 |
| 947 | GCST90319877 | IVW | 2 | 0.291 | 0.138 | 0.035 | 1.338 | 1.021 | 1.755 |
| 623 | GCST90319877 | IVW | 2 | -0.081 | 0.141 | 0.565 | 0.922 | 0.699 | 1.216 |
| 629 | GCST90319877 | IVW | 2 | -0.227 | 0.195 | 0.245 | 0.797 | 0.544 | 1.168 |
| 633 | GCST90319877 | IVW | 2 | -0.079 | 0.295 | 0.790 | 0.924 | 0.519 | 1.647 |
| 635 | GCST90319877 | IVW | 2 | -0.086 | 0.162 | 0.597 | 0.918 | 0.667 | 1.262 |
| 636 | GCST90319877 | IVW | 2 | -0.063 | 0.158 | 0.687 | 0.939 | 0.689 | 1.278 |
| 637 | GCST90319877 | IVW | 4 | 0.103 | 0.172 | 0.549 | 1.108 | 0.791 | 1.553 |
| 638 | GCST90319877 | IVW | 4 | 0.050 | 0.122 | 0.681 | 1.051 | 0.828 | 1.335 |
| 640 | GCST90319877 | IVW | 2 | 0.043 | 0.116 | 0.712 | 1.044 | 0.832 | 1.309 |
| 642 | GCST90319877 | IVW | 2 | 0.000 | 0.195 | 0.999 | 1.000 | 0.682 | 1.466 |
| 644 | GCST90319877 | IVW | 2 | -0.132 | 0.182 | 0.469 | 0.876 | 0.613 | 1.252 |
| 648 | GCST90319877 | IVW | 5 | -0.120 | 0.120 | 0.316 | 0.887 | 0.701 | 1.122 |
| 842 | GCST90319877 | IVW | 2 | 0.287 | 0.143 | 0.044 | 1.333 | 1.008 | 1.763 |
| 652 | GCST90319877 | IVW | 8 | 0.070 | 0.059 | 0.233 | 1.073 | 0.956 | 1.204 |
| 653 | GCST90319877 | IVW | 2 | 0.096 | 0.134 | 0.475 | 1.101 | 0.846 | 1.432 |
| 658 | GCST90319877 | IVW | 6 | 0.087 | 0.109 | 0.427 | 1.091 | 0.880 | 1.351 |
| 659 | GCST90319877 | IVW | 3 | 0.093 | 0.116 | 0.423 | 1.098 | 0.874 | 1.378 |
| 660 | GCST90319877 | IVW | 12 | 0.000 | 0.067 | 0.996 | 1.000 | 0.876 | 1.141 |
| 666 | GCST90319877 | IVW | 3 | -0.229 | 0.138 | 0.096 | 0.795 | 0.607 | 1.041 |
| 667 | GCST90319877 | IVW | 5 | 0.133 | 0.072 | 0.065 | 1.142 | 0.992 | 1.315 |
| 668 | GCST90319877 | IVW | 17 | 0.028 | 0.051 | 0.582 | 1.028 | 0.931 | 1.136 |
| 669 | GCST90319877 | IVW | 5 | 0.041 | 0.091 | 0.653 | 1.042 | 0.872 | 1.245 |
| 670 | GCST90319877 | IVW | 2 | -0.061 | 0.174 | 0.724 | 0.940 | 0.669 | 1.322 |
| 671 | GCST90319877 | IVW | 3 | 0.027 | 0.058 | 0.639 | 1.028 | 0.917 | 1.152 |
| 672 | GCST90319877 | IVW | 2 | 0.014 | 0.210 | 0.946 | 1.014 | 0.672 | 1.531 |
| 674 | GCST90319877 | IVW | 2 | 0.178 | 0.230 | 0.441 | 1.194 | 0.760 | 1.875 |
| 675 | GCST90319877 | IVW | 3 | 0.037 | 0.155 | 0.812 | 1.038 | 0.766 | 1.406 |
| 676 | GCST90319877 | IVW | 5 | 0.013 | 0.078 | 0.863 | 1.014 | 0.870 | 1.182 |
| 298 | GCST90319877 | IVW | 2 | 0.287 | 0.131 | 0.029 | 1.332 | 1.030 | 1.722 |
| 678 | GCST90319877 | IVW | 4 | -0.043 | 0.117 | 0.712 | 0.958 | 0.761 | 1.205 |
| 680 | GCST90319877 | IVW | 3 | 0.161 | 0.259 | 0.534 | 1.175 | 0.707 | 1.952 |
| 681 | GCST90319877 | IVW | 2 | 0.010 | 0.177 | 0.955 | 1.010 | 0.714 | 1.429 |
| 810 | GCST90319877 | IVW | 2 | -0.382 | 0.153 | 0.013 | 0.683 | 0.506 | 0.921 |
| 685 | GCST90319877 | IVW | 2 | 0.289 | 0.152 | 0.057 | 1.335 | 0.991 | 1.798 |
| 686 | GCST90319877 | IVW | 10 | 0.084 | 0.076 | 0.265 | 1.088 | 0.938 | 1.262 |
| 689 | GCST90319877 | IVW | 4 | -0.009 | 0.222 | 0.968 | 0.991 | 0.642 | 1.530 |
| 1191 | GCST90319877 | IVW | 2 | -0.373 | 0.168 | 0.026 | 0.689 | 0.496 | 0.957 |
| 692 | GCST90319877 | IVW | 6 | -0.091 | 0.087 | 0.295 | 0.913 | 0.770 | 1.083 |
| 693 | GCST90319877 | IVW | 2 | 0.102 | 0.131 | 0.435 | 1.108 | 0.857 | 1.432 |
| 694 | GCST90319877 | IVW | 11 | 0.024 | 0.066 | 0.717 | 1.024 | 0.900 | 1.165 |
| 695 | GCST90319877 | IVW | 2 | 0.375 | 0.250 | 0.133 | 1.455 | 0.892 | 2.373 |
| 1242 | GCST90319877 | IVW | 2 | -0.357 | 0.162 | 0.027 | 0.700 | 0.510 | 0.961 |
| 697 | GCST90319877 | IVW | 3 | -0.053 | 0.119 | 0.655 | 0.948 | 0.752 | 1.197 |
| 700 | GCST90319877 | IVW | 3 | 0.030 | 0.101 | 0.765 | 1.031 | 0.845 | 1.257 |
| 701 | GCST90319877 | IVW | 4 | 0.133 | 0.157 | 0.397 | 1.142 | 0.840 | 1.553 |
| 702 | GCST90319877 | IVW | 16 | -0.037 | 0.051 | 0.466 | 0.963 | 0.872 | 1.065 |
| 703 | GCST90319877 | IVW | 2 | 0.057 | 0.085 | 0.502 | 1.059 | 0.896 | 1.250 |
| 704 | GCST90319877 | IVW | 4 | -0.257 | 0.143 | 0.073 | 0.774 | 0.584 | 1.024 |
| 705 | GCST90319877 | IVW | 2 | 0.060 | 0.065 | 0.361 | 1.062 | 0.934 | 1.207 |
| 706 | GCST90319877 | IVW | 6 | 0.077 | 0.117 | 0.510 | 1.080 | 0.859 | 1.360 |
| 708 | GCST90319877 | IVW | 2 | -0.131 | 0.269 | 0.626 | 0.877 | 0.518 | 1.485 |
| 709 | GCST90319877 | IVW | 2 | 0.030 | 0.328 | 0.927 | 1.030 | 0.542 | 1.959 |
| 710 | GCST90319877 | IVW | 4 | -0.149 | 0.095 | 0.118 | 0.862 | 0.716 | 1.038 |
| 682 | GCST90319877 | IVW | 5 | -0.321 | 0.088 | 0.000 | 0.725 | 0.610 | 0.862 |
| 712 | GCST90319877 | IVW | 4 | -0.026 | 0.096 | 0.786 | 0.974 | 0.806 | 1.177 |
| 716 | GCST90319877 | IVW | 4 | -0.013 | 0.146 | 0.930 | 0.987 | 0.741 | 1.315 |
| 719 | GCST90319877 | IVW | 3 | -0.073 | 0.230 | 0.753 | 0.930 | 0.592 | 1.461 |
| 720 | GCST90319877 | IVW | 12 | 0.017 | 0.050 | 0.732 | 1.017 | 0.922 | 1.123 |
| 726 | GCST90319877 | IVW | 7 | 0.132 | 0.127 | 0.299 | 1.141 | 0.889 | 1.465 |
| 696 | GCST90319877 | IVW | 2 | -0.309 | 0.145 | 0.033 | 0.734 | 0.553 | 0.975 |
| 728 | GCST90319877 | IVW | 9 | -0.019 | 0.105 | 0.857 | 0.981 | 0.799 | 1.205 |
| 986 | GCST90319877 | IVW | 3 | -0.273 | 0.129 | 0.035 | 0.761 | 0.591 | 0.981 |
| 735 | GCST90319877 | IVW | 4 | -0.076 | 0.106 | 0.472 | 0.926 | 0.752 | 1.141 |
| 736 | GCST90319877 | IVW | 14 | 0.009 | 0.059 | 0.874 | 1.009 | 0.899 | 1.133 |
| 737 | GCST90319877 | IVW | 3 | 0.049 | 0.070 | 0.488 | 1.050 | 0.915 | 1.204 |
| 739 | GCST90319877 | IVW | 4 | 0.030 | 0.056 | 0.588 | 1.031 | 0.924 | 1.150 |
| 740 | GCST90319877 | IVW | 3 | 0.092 | 0.111 | 0.404 | 1.097 | 0.883 | 1.362 |
| 742 | GCST90319877 | IVW | 3 | 0.115 | 0.144 | 0.424 | 1.122 | 0.846 | 1.489 |
| 743 | GCST90319877 | IVW | 4 | 0.042 | 0.117 | 0.722 | 1.043 | 0.828 | 1.312 |
| 744 | GCST90319877 | IVW | 3 | -0.001 | 0.089 | 0.992 | 0.999 | 0.840 | 1.189 |
| 745 | GCST90319877 | IVW | 2 | 0.019 | 0.151 | 0.902 | 1.019 | 0.757 | 1.370 |
| 746 | GCST90319877 | IVW | 3 | 0.040 | 0.106 | 0.704 | 1.041 | 0.845 | 1.283 |
| 748 | GCST90319877 | IVW | 2 | 0.100 | 0.164 | 0.542 | 1.105 | 0.801 | 1.525 |
| 749 | GCST90319877 | IVW | 5 | -0.138 | 0.150 | 0.359 | 0.871 | 0.649 | 1.170 |
| 752 | GCST90319877 | IVW | 3 | 0.182 | 0.133 | 0.172 | 1.200 | 0.924 | 1.558 |
| 753 | GCST90319877 | IVW | 11 | 0.103 | 0.098 | 0.295 | 1.108 | 0.914 | 1.343 |
| 754 | GCST90319877 | IVW | 2 | -0.262 | 0.170 | 0.122 | 0.769 | 0.552 | 1.073 |
| 756 | GCST90319877 | IVW | 3 | -0.010 | 0.138 | 0.940 | 0.990 | 0.755 | 1.298 |
| 759 | GCST90319877 | IVW | 5 | -0.065 | 0.110 | 0.554 | 0.937 | 0.756 | 1.162 |
| 760 | GCST90319877 | IVW | 4 | 0.162 | 0.244 | 0.508 | 1.176 | 0.728 | 1.898 |
| 761 | GCST90319877 | IVW | 11 | 0.040 | 0.071 | 0.576 | 1.041 | 0.905 | 1.197 |
| 764 | GCST90319877 | IVW | 3 | -0.014 | 0.121 | 0.908 | 0.986 | 0.777 | 1.251 |
| 767 | GCST90319877 | IVW | 2 | -0.050 | 0.168 | 0.765 | 0.951 | 0.684 | 1.323 |
| 768 | GCST90319877 | IVW | 5 | 0.108 | 0.119 | 0.362 | 1.114 | 0.883 | 1.407 |
| 769 | GCST90319877 | IVW | 16 | 0.078 | 0.079 | 0.326 | 1.081 | 0.925 | 1.263 |
| 770 | GCST90319877 | IVW | 2 | 0.049 | 0.074 | 0.502 | 1.051 | 0.909 | 1.214 |
| 772 | GCST90319877 | IVW | 2 | 0.063 | 0.064 | 0.323 | 1.065 | 0.940 | 1.208 |
| 773 | GCST90319877 | IVW | 2 | 0.206 | 0.150 | 0.171 | 1.228 | 0.915 | 1.650 |
| 775 | GCST90319877 | IVW | 2 | 0.043 | 0.205 | 0.834 | 1.044 | 0.698 | 1.560 |
| 776 | GCST90319877 | IVW | 2 | 0.242 | 0.154 | 0.116 | 1.274 | 0.942 | 1.723 |
| 777 | GCST90319877 | IVW | 5 | -0.034 | 0.083 | 0.680 | 0.966 | 0.821 | 1.138 |
| 779 | GCST90319877 | IVW | 4 | -0.024 | 0.095 | 0.798 | 0.976 | 0.810 | 1.176 |
| 782 | GCST90319877 | IVW | 2 | -0.062 | 0.117 | 0.597 | 0.940 | 0.748 | 1.182 |
| 783 | GCST90319877 | IVW | 2 | 0.058 | 0.102 | 0.569 | 1.060 | 0.868 | 1.294 |
| 784 | GCST90319877 | IVW | 2 | 0.065 | 0.068 | 0.342 | 1.067 | 0.934 | 1.219 |
| 785 | GCST90319877 | IVW | 4 | 0.000 | 0.077 | 0.996 | 1.000 | 0.861 | 1.163 |
| 786 | GCST90319877 | IVW | 2 | -0.220 | 0.144 | 0.127 | 0.803 | 0.605 | 1.064 |
| 787 | GCST90319877 | IVW | 3 | 0.060 | 0.081 | 0.465 | 1.061 | 0.905 | 1.245 |
| 788 | GCST90319877 | IVW | 3 | 0.217 | 0.119 | 0.068 | 1.243 | 0.984 | 1.569 |
| 790 | GCST90319877 | IVW | 4 | 0.019 | 0.081 | 0.816 | 1.019 | 0.869 | 1.195 |
| 791 | GCST90319877 | IVW | 19 | 0.007 | 0.048 | 0.886 | 1.007 | 0.916 | 1.107 |
| 792 | GCST90319877 | IVW | 13 | 0.023 | 0.063 | 0.714 | 1.023 | 0.904 | 1.158 |
| 795 | GCST90319877 | IVW | 2 | 0.019 | 0.130 | 0.885 | 1.019 | 0.790 | 1.313 |
| 796 | GCST90319877 | IVW | 2 | 0.048 | 0.115 | 0.680 | 1.049 | 0.837 | 1.315 |
| 797 | GCST90319877 | IVW | 2 | -0.014 | 0.108 | 0.895 | 0.986 | 0.798 | 1.218 |
| 799 | GCST90319877 | IVW | 3 | 0.019 | 0.061 | 0.753 | 1.020 | 0.904 | 1.150 |
| 801 | GCST90319877 | IVW | 4 | 0.098 | 0.058 | 0.093 | 1.103 | 0.984 | 1.236 |
| 802 | GCST90319877 | IVW | 5 | 0.027 | 0.133 | 0.842 | 1.027 | 0.791 | 1.334 |
| 804 | GCST90319877 | IVW | 3 | 0.158 | 0.139 | 0.256 | 1.171 | 0.892 | 1.539 |
| 805 | GCST90319877 | IVW | 16 | 0.008 | 0.050 | 0.876 | 1.008 | 0.914 | 1.111 |
| 806 | GCST90319877 | IVW | 13 | -0.019 | 0.058 | 0.749 | 0.982 | 0.876 | 1.100 |
| 807 | GCST90319877 | IVW | 2 | 0.000 | 0.129 | 1.000 | 1.000 | 0.776 | 1.289 |
| 808 | GCST90319877 | IVW | 3 | 0.003 | 0.128 | 0.979 | 1.003 | 0.781 | 1.290 |
| 809 | GCST90319877 | IVW | 2 | -0.116 | 0.154 | 0.452 | 0.891 | 0.659 | 1.204 |
| 727 | GCST90319877 | IVW | 3 | -0.256 | 0.119 | 0.032 | 0.774 | 0.613 | 0.978 |
| 811 | GCST90319877 | IVW | 3 | 0.285 | 0.128 | 0.026 | 1.330 | 1.035 | 1.709 |
| 812 | GCST90319877 | IVW | 6 | 0.104 | 0.070 | 0.136 | 1.109 | 0.968 | 1.271 |
| 818 | GCST90319877 | IVW | 6 | -0.002 | 0.079 | 0.977 | 0.998 | 0.854 | 1.166 |
| 819 | GCST90319877 | IVW | 4 | 0.065 | 0.100 | 0.515 | 1.067 | 0.877 | 1.298 |
| 820 | GCST90319877 | IVW | 13 | 0.010 | 0.064 | 0.875 | 1.010 | 0.891 | 1.145 |
| 1044 | GCST90319877 | IVW | 5 | -0.237 | 0.113 | 0.036 | 0.789 | 0.632 | 0.984 |
| 823 | GCST90319877 | IVW | 3 | -0.121 | 0.136 | 0.375 | 0.886 | 0.679 | 1.157 |
| 824 | GCST90319877 | IVW | 2 | -0.189 | 0.676 | 0.779 | 0.827 | 0.220 | 3.115 |
| 827 | GCST90319877 | IVW | 6 | -0.080 | 0.068 | 0.242 | 0.923 | 0.808 | 1.055 |
| 828 | GCST90319877 | IVW | 18 | 0.019 | 0.051 | 0.706 | 1.019 | 0.923 | 1.126 |
| 829 | GCST90319877 | IVW | 6 | 0.062 | 0.090 | 0.493 | 1.064 | 0.892 | 1.269 |
| 830 | GCST90319877 | IVW | 2 | -0.151 | 0.149 | 0.311 | 0.860 | 0.642 | 1.152 |
| 831 | GCST90319877 | IVW | 3 | 0.027 | 0.058 | 0.634 | 1.028 | 0.918 | 1.151 |
| 832 | GCST90319877 | IVW | 3 | -0.015 | 0.129 | 0.907 | 0.985 | 0.766 | 1.268 |
| 834 | GCST90319877 | IVW | 3 | -0.059 | 0.227 | 0.796 | 0.943 | 0.604 | 1.472 |
| 835 | GCST90319877 | IVW | 4 | 0.054 | 0.151 | 0.721 | 1.055 | 0.785 | 1.420 |
| 836 | GCST90319877 | IVW | 6 | -0.004 | 0.072 | 0.951 | 0.996 | 0.865 | 1.146 |
| 837 | GCST90319877 | IVW | 2 | 0.172 | 0.145 | 0.235 | 1.187 | 0.894 | 1.576 |
| 838 | GCST90319877 | IVW | 3 | -0.023 | 0.156 | 0.881 | 0.977 | 0.720 | 1.325 |
| 839 | GCST90319877 | IVW | 3 | 0.158 | 0.255 | 0.535 | 1.171 | 0.711 | 1.929 |
| 840 | GCST90319877 | IVW | 6 | -0.042 | 0.087 | 0.626 | 0.959 | 0.809 | 1.136 |
| 651 | GCST90319877 | IVW | 3 | 0.285 | 0.128 | 0.026 | 1.330 | 1.035 | 1.708 |
| 843 | GCST90319877 | IVW | 9 | 0.072 | 0.086 | 0.407 | 1.074 | 0.907 | 1.272 |
| 845 | GCST90319877 | IVW | 2 | 0.037 | 0.151 | 0.807 | 1.038 | 0.771 | 1.396 |
| 846 | GCST90319877 | IVW | 3 | 0.088 | 0.213 | 0.679 | 1.092 | 0.719 | 1.658 |
| 847 | GCST90319877 | IVW | 2 | -0.209 | 0.161 | 0.195 | 0.812 | 0.592 | 1.113 |
| 861 | GCST90319877 | IVW | 5 | -0.237 | 0.111 | 0.034 | 0.789 | 0.634 | 0.982 |
| 849 | GCST90319877 | IVW | 6 | -0.088 | 0.086 | 0.303 | 0.915 | 0.774 | 1.083 |
| 850 | GCST90319877 | IVW | 3 | 0.257 | 0.277 | 0.353 | 1.293 | 0.752 | 2.224 |
| 851 | GCST90319877 | IVW | 10 | 0.048 | 0.063 | 0.450 | 1.049 | 0.926 | 1.188 |
| 852 | GCST90319877 | IVW | 2 | 0.136 | 0.168 | 0.419 | 1.146 | 0.824 | 1.594 |
| 853 | GCST90319877 | IVW | 4 | -0.186 | 0.188 | 0.323 | 0.831 | 0.575 | 1.201 |
| 854 | GCST90319877 | IVW | 2 | -0.021 | 0.132 | 0.874 | 0.979 | 0.757 | 1.267 |
| 855 | GCST90319877 | IVW | 3 | 0.125 | 0.128 | 0.328 | 1.133 | 0.882 | 1.455 |
| 858 | GCST90319877 | IVW | 3 | 0.164 | 0.134 | 0.221 | 1.178 | 0.906 | 1.531 |
| 859 | GCST90319877 | IVW | 16 | -0.036 | 0.050 | 0.476 | 0.965 | 0.874 | 1.065 |
| 860 | GCST90319877 | IVW | 3 | -0.053 | 0.084 | 0.524 | 0.948 | 0.805 | 1.117 |
| 138 | GCST90319877 | IVW | 4 | -0.236 | 0.107 | 0.027 | 0.790 | 0.641 | 0.974 |
| 862 | GCST90319877 | IVW | 2 | 0.058 | 0.063 | 0.363 | 1.059 | 0.936 | 1.199 |
| 863 | GCST90319877 | IVW | 6 | 0.056 | 0.136 | 0.681 | 1.058 | 0.810 | 1.380 |
| 865 | GCST90319877 | IVW | 2 | -0.200 | 0.152 | 0.190 | 0.819 | 0.608 | 1.104 |
| 867 | GCST90319877 | IVW | 6 | -0.151 | 0.080 | 0.059 | 0.860 | 0.736 | 1.006 |
| 868 | GCST90319877 | IVW | 3 | -0.171 | 0.130 | 0.191 | 0.843 | 0.653 | 1.089 |
| 869 | GCST90319877 | IVW | 4 | -0.030 | 0.095 | 0.755 | 0.971 | 0.805 | 1.170 |
| 870 | GCST90319877 | IVW | 3 | -0.074 | 0.135 | 0.586 | 0.929 | 0.713 | 1.211 |
| 871 | GCST90319877 | IVW | 5 | 0.001 | 0.091 | 0.989 | 1.001 | 0.837 | 1.197 |
| 873 | GCST90319877 | IVW | 2 | -0.251 | 0.157 | 0.109 | 0.778 | 0.573 | 1.058 |
| 874 | GCST90319877 | IVW | 2 | 0.306 | 0.380 | 0.421 | 1.358 | 0.644 | 2.862 |
| 875 | GCST90319877 | IVW | 3 | -0.062 | 0.089 | 0.490 | 0.940 | 0.790 | 1.120 |
| 878 | GCST90319877 | IVW | 2 | 0.181 | 0.305 | 0.552 | 1.199 | 0.660 | 2.178 |
| 879 | GCST90319877 | IVW | 2 | -0.117 | 0.160 | 0.465 | 0.890 | 0.651 | 1.217 |
| 881 | GCST90319877 | IVW | 3 | -0.029 | 0.139 | 0.834 | 0.971 | 0.740 | 1.274 |
| 882 | GCST90319877 | IVW | 10 | 0.031 | 0.059 | 0.596 | 1.032 | 0.919 | 1.159 |
| 885 | GCST90319877 | IVW | 4 | 0.154 | 0.098 | 0.116 | 1.166 | 0.963 | 1.412 |
| 887 | GCST90319877 | IVW | 5 | -0.077 | 0.150 | 0.608 | 0.926 | 0.690 | 1.243 |
| 889 | GCST90319877 | IVW | 3 | 0.179 | 0.117 | 0.126 | 1.196 | 0.951 | 1.503 |
| 893 | GCST90319877 | IVW | 10 | -0.028 | 0.081 | 0.734 | 0.973 | 0.830 | 1.141 |
| 895 | GCST90319877 | IVW | 2 | -0.094 | 0.171 | 0.585 | 0.911 | 0.651 | 1.274 |
| 897 | GCST90319877 | IVW | 3 | -0.014 | 0.131 | 0.912 | 0.986 | 0.762 | 1.275 |
| 898 | GCST90319877 | IVW | 2 | -0.012 | 0.111 | 0.917 | 0.988 | 0.795 | 1.229 |
| 899 | GCST90319877 | IVW | 2 | 0.061 | 0.108 | 0.570 | 1.063 | 0.861 | 1.313 |
| 900 | GCST90319877 | IVW | 4 | 0.003 | 0.063 | 0.959 | 1.003 | 0.886 | 1.136 |
| 901 | GCST90319877 | IVW | 2 | 0.069 | 0.109 | 0.527 | 1.071 | 0.865 | 1.327 |
| 911 | GCST90319877 | IVW | 2 | -0.053 | 0.139 | 0.700 | 0.948 | 0.722 | 1.244 |
| 912 | GCST90319877 | IVW | 3 | -0.185 | 0.125 | 0.139 | 0.831 | 0.651 | 1.062 |
| 822 | GCST90319877 | IVW | 4 | -0.228 | 0.108 | 0.034 | 0.796 | 0.645 | 0.983 |
| 915 | GCST90319877 | IVW | 17 | 0.056 | 0.055 | 0.315 | 1.057 | 0.949 | 1.178 |
| 919 | GCST90319877 | IVW | 2 | -0.049 | 0.232 | 0.832 | 0.952 | 0.604 | 1.500 |
| 920 | GCST90319877 | IVW | 5 | 0.111 | 0.092 | 0.227 | 1.118 | 0.933 | 1.339 |
| 921 | GCST90319877 | IVW | 2 | 0.190 | 0.153 | 0.215 | 1.209 | 0.896 | 1.631 |
| 925 | GCST90319877 | IVW | 2 | 0.140 | 0.282 | 0.620 | 1.150 | 0.662 | 1.998 |
| 928 | GCST90319877 | IVW | 3 | -0.033 | 0.146 | 0.821 | 0.968 | 0.727 | 1.287 |
| 934 | GCST90319877 | IVW | 4 | -0.078 | 0.094 | 0.412 | 0.925 | 0.769 | 1.113 |
| 935 | GCST90319877 | IVW | 5 | -0.014 | 0.097 | 0.886 | 0.986 | 0.815 | 1.193 |
| 936 | GCST90319877 | IVW | 4 | 0.112 | 0.141 | 0.427 | 1.118 | 0.849 | 1.473 |
| 938 | GCST90319877 | IVW | 4 | 0.080 | 0.082 | 0.334 | 1.083 | 0.921 | 1.273 |
| 939 | GCST90319877 | IVW | 2 | 0.083 | 0.143 | 0.565 | 1.086 | 0.820 | 1.439 |
| 942 | GCST90319877 | IVW | 3 | 0.124 | 0.092 | 0.176 | 1.132 | 0.946 | 1.356 |
| 944 | GCST90319877 | IVW | 4 | -0.099 | 0.112 | 0.377 | 0.906 | 0.727 | 1.128 |
| 235 | GCST90319877 | IVW | 3 | 0.278 | 0.121 | 0.021 | 1.320 | 1.042 | 1.673 |
| 956 | GCST90319877 | IVW | 15 | 0.123 | 0.080 | 0.126 | 1.131 | 0.966 | 1.323 |
| 958 | GCST90319877 | IVW | 2 | -0.125 | 0.150 | 0.405 | 0.882 | 0.657 | 1.185 |
| 959 | GCST90319877 | IVW | 3 | 0.097 | 0.226 | 0.670 | 1.101 | 0.707 | 1.717 |
| 965 | GCST90319877 | IVW | 3 | -0.104 | 0.170 | 0.540 | 0.901 | 0.647 | 1.257 |
| 967 | GCST90319877 | IVW | 8 | 0.059 | 0.080 | 0.457 | 1.061 | 0.908 | 1.241 |
| 970 | GCST90319877 | IVW | 2 | -0.201 | 0.179 | 0.262 | 0.818 | 0.576 | 1.162 |
| 971 | GCST90319877 | IVW | 3 | 0.111 | 0.091 | 0.223 | 1.118 | 0.934 | 1.337 |
| 972 | GCST90319877 | IVW | 3 | 0.031 | 0.104 | 0.768 | 1.031 | 0.841 | 1.265 |
| 973 | GCST90319877 | IVW | 2 | -0.059 | 0.112 | 0.597 | 0.942 | 0.756 | 1.174 |
| 974 | GCST90319877 | IVW | 2 | 0.071 | 0.076 | 0.353 | 1.073 | 0.924 | 1.246 |
| 975 | GCST90319877 | IVW | 4 | -0.092 | 0.190 | 0.628 | 0.912 | 0.628 | 1.324 |
| 983 | GCST90319877 | IVW | 2 | -0.226 | 0.252 | 0.369 | 0.797 | 0.486 | 1.307 |
| 277 | GCST90319877 | IVW | 5 | -0.213 | 0.094 | 0.024 | 0.809 | 0.672 | 0.972 |
| 987 | GCST90319877 | IVW | 10 | 0.011 | 0.062 | 0.855 | 1.011 | 0.896 | 1.142 |
| 988 | GCST90319877 | IVW | 2 | -0.049 | 0.160 | 0.758 | 0.952 | 0.696 | 1.302 |
| 989 | GCST90319877 | IVW | 13 | -0.015 | 0.069 | 0.829 | 0.985 | 0.861 | 1.128 |
| 990 | GCST90319877 | IVW | 3 | 0.036 | 0.070 | 0.603 | 1.037 | 0.904 | 1.190 |
| 994 | GCST90319877 | IVW | 2 | 0.225 | 0.231 | 0.330 | 1.252 | 0.796 | 1.969 |
| 995 | GCST90319877 | IVW | 3 | -0.065 | 0.104 | 0.529 | 0.937 | 0.764 | 1.148 |
| 997 | GCST90319877 | IVW | 2 | 0.083 | 0.136 | 0.541 | 1.086 | 0.833 | 1.418 |
| 1001 | GCST90319877 | IVW | 2 | -0.208 | 0.177 | 0.239 | 0.812 | 0.574 | 1.148 |
| 1002 | GCST90319877 | IVW | 2 | 0.123 | 0.137 | 0.373 | 1.130 | 0.863 | 1.480 |
| 1005 | GCST90319877 | IVW | 2 | -0.262 | 0.192 | 0.173 | 0.770 | 0.528 | 1.122 |
| 1006 | GCST90319877 | IVW | 3 | -0.060 | 0.108 | 0.582 | 0.942 | 0.762 | 1.165 |
| 1008 | GCST90319877 | IVW | 3 | 0.122 | 0.289 | 0.672 | 1.130 | 0.641 | 1.993 |
| 1009 | GCST90319877 | IVW | 2 | -0.044 | 0.294 | 0.881 | 0.957 | 0.538 | 1.702 |
| 1010 | GCST90319877 | IVW | 5 | 0.079 | 0.115 | 0.492 | 1.082 | 0.864 | 1.355 |
| 1012 | GCST90319877 | IVW | 3 | -0.021 | 0.095 | 0.825 | 0.979 | 0.814 | 1.179 |
| 1016 | GCST90319877 | IVW | 3 | 0.040 | 0.160 | 0.805 | 1.040 | 0.760 | 1.423 |
| 1020 | GCST90319877 | IVW | 4 | 0.145 | 0.202 | 0.471 | 1.156 | 0.779 | 1.717 |
| 1023 | GCST90319877 | IVW | 2 | -0.174 | 0.149 | 0.242 | 0.840 | 0.627 | 1.125 |
| 1024 | GCST90319877 | IVW | 2 | -0.200 | 0.121 | 0.097 | 0.819 | 0.646 | 1.037 |
| 1026 | GCST90319877 | IVW | 2 | -0.388 | 0.376 | 0.302 | 0.679 | 0.325 | 1.417 |
| 1027 | GCST90319877 | IVW | 4 | 0.063 | 0.122 | 0.608 | 1.065 | 0.838 | 1.352 |
| 1029 | GCST90319877 | IVW | 2 | 0.155 | 0.160 | 0.332 | 1.168 | 0.854 | 1.598 |
| 1030 | GCST90319877 | IVW | 2 | -0.124 | 0.164 | 0.449 | 0.883 | 0.641 | 1.218 |
| 1039 | GCST90319877 | IVW | 2 | -0.072 | 0.421 | 0.863 | 0.930 | 0.407 | 2.124 |
| 913 | GCST90319877 | IVW | 5 | -0.209 | 0.093 | 0.024 | 0.812 | 0.677 | 0.973 |
| 1047 | GCST90319877 | IVW | 4 | 0.034 | 0.178 | 0.847 | 1.035 | 0.730 | 1.467 |
| 1048 | GCST90319877 | IVW | 6 | 0.116 | 0.107 | 0.277 | 1.123 | 0.911 | 1.383 |
| 1050 | GCST90319877 | IVW | 2 | 0.137 | 0.307 | 0.655 | 1.147 | 0.628 | 2.094 |
| 1054 | GCST90319877 | IVW | 4 | 0.267 | 0.208 | 0.199 | 1.306 | 0.869 | 1.962 |
| 1058 | GCST90319877 | IVW | 2 | -0.218 | 0.127 | 0.086 | 0.804 | 0.627 | 1.031 |
| 1063 | GCST90319877 | IVW | 2 | 0.173 | 0.178 | 0.331 | 1.189 | 0.839 | 1.685 |
| 1064 | GCST90319877 | IVW | 4 | -0.058 | 0.120 | 0.627 | 0.943 | 0.745 | 1.194 |
| 1065 | GCST90319877 | IVW | 2 | -0.144 | 0.200 | 0.472 | 0.866 | 0.586 | 1.281 |
| 1070 | GCST90319877 | IVW | 3 | -0.233 | 0.216 | 0.281 | 0.792 | 0.519 | 1.210 |
| 1075 | GCST90319877 | IVW | 2 | -0.015 | 0.154 | 0.921 | 0.985 | 0.729 | 1.331 |
| 1076 | GCST90319877 | IVW | 3 | -0.057 | 0.163 | 0.729 | 0.945 | 0.686 | 1.301 |
| 1078 | GCST90319877 | IVW | 5 | -0.150 | 0.196 | 0.446 | 0.861 | 0.586 | 1.265 |
| 239 | GCST90319877 | IVW | 2 | 0.208 | 0.099 | 0.036 | 1.232 | 1.014 | 1.496 |
| 237 | GCST90319877 | IVW | 3 | 0.202 | 0.081 | 0.012 | 1.224 | 1.045 | 1.434 |
| 1082 | GCST90319877 | IVW | 3 | 0.013 | 0.176 | 0.942 | 1.013 | 0.717 | 1.431 |
| 1083 | GCST90319877 | IVW | 3 | -0.187 | 0.117 | 0.111 | 0.830 | 0.659 | 1.044 |
| 1084 | GCST90319877 | IVW | 2 | 0.053 | 0.286 | 0.852 | 1.055 | 0.602 | 1.847 |
| 1088 | GCST90319877 | IVW | 2 | -0.047 | 0.061 | 0.443 | 0.954 | 0.847 | 1.075 |
| 1094 | GCST90319877 | IVW | 3 | 0.159 | 0.149 | 0.286 | 1.172 | 0.875 | 1.569 |
| 1095 | GCST90319877 | IVW | 2 | -0.161 | 0.145 | 0.267 | 0.851 | 0.640 | 1.131 |
| 1096 | GCST90319877 | IVW | 3 | -0.141 | 0.250 | 0.574 | 0.869 | 0.533 | 1.418 |
| 1098 | GCST90319877 | IVW | 2 | -0.173 | 0.134 | 0.199 | 0.841 | 0.647 | 1.095 |
| 1106 | GCST90319877 | IVW | 2 | -0.143 | 0.170 | 0.401 | 0.867 | 0.620 | 1.210 |
| 1110 | GCST90319877 | IVW | 2 | 0.052 | 0.266 | 0.846 | 1.053 | 0.625 | 1.773 |
| 1112 | GCST90319877 | IVW | 2 | -0.025 | 0.302 | 0.934 | 0.975 | 0.539 | 1.764 |
| 1113 | GCST90319877 | IVW | 2 | 0.097 | 0.401 | 0.809 | 1.102 | 0.502 | 2.416 |
| 1117 | GCST90319877 | IVW | 2 | -0.174 | 0.149 | 0.243 | 0.840 | 0.627 | 1.126 |
| 1118 | GCST90319877 | IVW | 2 | -0.224 | 0.132 | 0.090 | 0.799 | 0.617 | 1.036 |
| 1121 | GCST90319877 | IVW | 3 | 0.214 | 0.119 | 0.072 | 1.238 | 0.981 | 1.562 |
| 1123 | GCST90319877 | IVW | 3 | 0.146 | 0.131 | 0.263 | 1.158 | 0.896 | 1.497 |
| 1124 | GCST90319877 | IVW | 2 | -0.123 | 0.162 | 0.448 | 0.884 | 0.644 | 1.214 |
| 1131 | GCST90319877 | IVW | 2 | 0.325 | 0.172 | 0.059 | 1.384 | 0.988 | 1.939 |
| 1133 | GCST90319877 | IVW | 3 | -0.067 | 0.262 | 0.797 | 0.935 | 0.559 | 1.562 |
| 1138 | GCST90319877 | IVW | 4 | -0.170 | 0.129 | 0.188 | 0.843 | 0.654 | 1.087 |
| 1139 | GCST90319877 | IVW | 2 | 0.055 | 0.182 | 0.761 | 1.057 | 0.740 | 1.509 |
| 1141 | GCST90319877 | IVW | 3 | 0.069 | 0.230 | 0.766 | 1.071 | 0.682 | 1.683 |
| 1142 | GCST90319877 | IVW | 5 | 0.188 | 0.102 | 0.065 | 1.207 | 0.989 | 1.473 |
| 1149 | GCST90319877 | IVW | 2 | -0.119 | 0.179 | 0.507 | 0.888 | 0.625 | 1.261 |
| 1153 | GCST90319877 | IVW | 2 | 0.097 | 0.425 | 0.820 | 1.102 | 0.479 | 2.533 |
| 1154 | GCST90319877 | IVW | 2 | -0.039 | 0.215 | 0.856 | 0.962 | 0.631 | 1.466 |
| 1155 | GCST90319877 | IVW | 3 | -0.020 | 0.155 | 0.900 | 0.981 | 0.724 | 1.329 |
| 1161 | GCST90319877 | IVW | 4 | -0.181 | 0.185 | 0.328 | 0.835 | 0.581 | 1.199 |
| 238 | GCST90319877 | IVW | 2 | 0.172 | 0.083 | 0.039 | 1.187 | 1.009 | 1.397 |
| 1166 | GCST90319877 | IVW | 3 | 0.007 | 0.117 | 0.952 | 1.007 | 0.801 | 1.267 |
| 1169 | GCST90319877 | IVW | 5 | -0.158 | 0.196 | 0.418 | 0.854 | 0.582 | 1.252 |
| 1171 | GCST90319877 | IVW | 3 | 0.179 | 0.242 | 0.461 | 1.196 | 0.743 | 1.923 |
| 16 | GCST90319877 | IVW | 9 | 0.168 | 0.071 | 0.018 | 1.183 | 1.029 | 1.361 |
| 1173 | GCST90319877 | IVW | 3 | -0.153 | 0.136 | 0.261 | 0.858 | 0.657 | 1.121 |
| 1175 | GCST90319877 | IVW | 3 | -0.058 | 0.214 | 0.786 | 0.943 | 0.620 | 1.437 |
| 1180 | GCST90319877 | IVW | 2 | -0.129 | 0.205 | 0.528 | 0.879 | 0.589 | 1.313 |
| 1181 | GCST90319877 | IVW | 3 | -0.054 | 0.225 | 0.809 | 0.947 | 0.609 | 1.473 |
| 1187 | GCST90319877 | IVW | 2 | 0.023 | 0.234 | 0.923 | 1.023 | 0.646 | 1.619 |
| 1188 | GCST90319877 | IVW | 2 | -0.248 | 0.139 | 0.074 | 0.781 | 0.595 | 1.024 |
| 15 | GCST90319877 | IVW | 5 | -0.207 | 0.092 | 0.025 | 0.813 | 0.678 | 0.975 |
| 1192 | GCST90319877 | IVW | 4 | 0.096 | 0.106 | 0.366 | 1.101 | 0.894 | 1.355 |
| 1193 | GCST90319877 | IVW | 5 | 0.048 | 0.140 | 0.730 | 1.049 | 0.798 | 1.381 |
| 125 | GCST90319877 | IVW | 9 | 0.167 | 0.078 | 0.033 | 1.182 | 1.014 | 1.378 |
| 1197 | GCST90319877 | IVW | 2 | 0.059 | 0.397 | 0.881 | 1.061 | 0.488 | 2.308 |
| 1205 | GCST90319877 | IVW | 3 | -0.013 | 0.090 | 0.887 | 0.987 | 0.827 | 1.178 |
| 1207 | GCST90319877 | IVW | 3 | -0.351 | 0.269 | 0.191 | 0.704 | 0.416 | 1.192 |
| 1214 | GCST90319877 | IVW | 2 | 0.427 | 0.451 | 0.344 | 1.532 | 0.634 | 3.705 |
| 1215 | GCST90319877 | IVW | 4 | 0.088 | 0.134 | 0.510 | 1.092 | 0.840 | 1.419 |
| 1218 | GCST90319877 | IVW | 2 | 0.260 | 0.166 | 0.116 | 1.297 | 0.938 | 1.795 |
| 1219 | GCST90319877 | IVW | 2 | 0.186 | 0.137 | 0.177 | 1.204 | 0.920 | 1.576 |
| 1231 | GCST90319877 | IVW | 4 | -0.070 | 0.110 | 0.524 | 0.933 | 0.752 | 1.156 |
| 1241 | GCST90319877 | IVW | 2 | -0.304 | 0.199 | 0.127 | 0.738 | 0.500 | 1.090 |
| 255 | GCST90319877 | IVW | 6 | -0.168 | 0.081 | 0.037 | 0.845 | 0.722 | 0.990 |
| 1245 | GCST90319877 | IVW | 2 | -0.036 | 0.265 | 0.892 | 0.965 | 0.574 | 1.621 |
| 1259 | GCST90319877 | IVW | 2 | 0.218 | 0.158 | 0.168 | 1.244 | 0.912 | 1.695 |
| 1266 | GCST90319877 | IVW | 5 | 0.185 | 0.150 | 0.216 | 1.203 | 0.897 | 1.613 |
| 202 | GCST90319877 | IVW | 9 | 0.161 | 0.077 | 0.036 | 1.175 | 1.011 | 1.366 |
| 1271 | GCST90319877 | IVW | 2 | -0.165 | 0.166 | 0.321 | 0.848 | 0.612 | 1.175 |
| 1273 | GCST90319877 | IVW | 3 | -0.132 | 0.131 | 0.311 | 0.876 | 0.678 | 1.132 |
| 1276 | GCST90319877 | IVW | 2 | 0.071 | 0.196 | 0.719 | 1.073 | 0.730 | 1.577 |
| 1277 | GCST90319877 | IVW | 2 | 0.008 | 0.229 | 0.972 | 1.008 | 0.643 | 1.579 |
| 1279 | GCST90319877 | IVW | 3 | -0.027 | 0.084 | 0.744 | 0.973 | 0.826 | 1.147 |
| 1281 | GCST90319877 | IVW | 5 | -0.109 | 0.210 | 0.604 | 0.897 | 0.594 | 1.353 |
| 1289 | GCST90319877 | IVW | 3 | -0.160 | 0.140 | 0.255 | 0.852 | 0.647 | 1.122 |
| 1294 | GCST90319877 | IVW | 2 | 0.090 | 0.147 | 0.540 | 1.094 | 0.820 | 1.460 |
| 1296 | GCST90319877 | IVW | 2 | -0.230 | 0.142 | 0.104 | 0.794 | 0.602 | 1.049 |
| 1305 | GCST90319877 | IVW | 3 | -0.217 | 0.153 | 0.157 | 0.805 | 0.596 | 1.087 |
| 1312 | GCST90319877 | IVW | 3 | -0.111 | 0.186 | 0.550 | 0.895 | 0.621 | 1.289 |

BIDPs=brain imaging-derived phenotypes;IVW=inverse-variance weighted;nSNPs=number of single nucleotide polymorphisms;OR=odds ratio;SE=standard error;CI=confidence interval;Pval=P value.

| **Table S2: MR results in replication cohort** | | | | | | | | | |
| --- | --- | --- | --- | --- | --- | --- | --- | --- | --- |
| BIDPs id | Outcome | OR.method | nSNPs | BETA | SE | Pval | OR | OR_lci95 | OR_uci95 |
| 1 | GCST90319878 | IVW | 4 | -0.146 | 0.130 | 0.261 | 0.864 | 0.670 | 1.115 |
| 2 | GCST90319878 | IVW | 3 | -0.089 | 0.155 | 0.565 | 0.915 | 0.676 | 1.239 |
| 3 | GCST90319878 | IVW | 5 | 0.061 | 0.076 | 0.422 | 1.063 | 0.915 | 1.235 |
| 4 | GCST90319878 | IVW | 5 | 0.061 | 0.077 | 0.422 | 1.063 | 0.915 | 1.236 |
| 5 | GCST90319878 | IVW | 3 | -0.106 | 0.143 | 0.457 | 0.899 | 0.680 | 1.189 |
| 6 | GCST90319878 | IVW | 3 | 0.062 | 0.129 | 0.629 | 1.064 | 0.826 | 1.372 |
| 7 | GCST90319878 | IVW | 8 | -0.023 | 0.101 | 0.823 | 0.978 | 0.802 | 1.191 |
| 8 | GCST90319878 | IVW | 8 | -0.022 | 0.101 | 0.825 | 0.978 | 0.802 | 1.192 |
| 9 | GCST90319878 | IVW | 4 | -0.050 | 0.114 | 0.663 | 0.952 | 0.761 | 1.190 |
| 10 | GCST90319878 | IVW | 4 | -0.047 | 0.114 | 0.679 | 0.954 | 0.762 | 1.193 |
| 11 | GCST90319878 | IVW | 3 | 0.087 | 0.147 | 0.553 | 1.091 | 0.818 | 1.455 |
| 12 | GCST90319878 | IVW | 3 | -0.054 | 0.122 | 0.659 | 0.948 | 0.746 | 1.203 |
| 13 | GCST90319878 | IVW | 11 | -0.013 | 0.058 | 0.816 | 0.987 | 0.881 | 1.105 |
| 14 | GCST90319878 | IVW | 9 | -0.021 | 0.062 | 0.733 | 0.979 | 0.867 | 1.105 |
| 15 | GCST90319878 | IVW | 5 | -0.115 | 0.098 | 0.238 | 0.891 | 0.736 | 1.079 |
| 16 | GCST90319878 | IVW | 9 | 0.130 | 0.074 | 0.077 | 1.139 | 0.986 | 1.315 |
| 17 | GCST90319878 | IVW | 5 | 0.116 | 0.148 | 0.431 | 1.123 | 0.841 | 1.501 |
| 18 | GCST90319878 | IVW | 3 | 0.109 | 0.085 | 0.200 | 1.115 | 0.944 | 1.316 |
| 19 | GCST90319878 | IVW | 4 | -0.013 | 0.116 | 0.911 | 0.987 | 0.787 | 1.239 |
| 20 | GCST90319878 | IVW | 3 | -0.130 | 0.123 | 0.291 | 0.878 | 0.690 | 1.118 |
| 24 | GCST90319878 | IVW | 2 | -0.082 | 0.142 | 0.567 | 0.922 | 0.697 | 1.218 |
| 25 | GCST90319878 | IVW | 11 | -0.078 | 0.095 | 0.410 | 0.925 | 0.767 | 1.114 |
| 28 | GCST90319878 | IVW | 5 | -0.148 | 0.091 | 0.102 | 0.862 | 0.722 | 1.030 |
| 29 | GCST90319878 | IVW | 8 | -0.019 | 0.076 | 0.798 | 0.981 | 0.846 | 1.138 |
| 32 | GCST90319878 | IVW | 2 | -0.266 | 0.138 | 0.054 | 0.766 | 0.584 | 1.005 |
| 33 | GCST90319878 | IVW | 3 | 0.186 | 0.166 | 0.263 | 1.204 | 0.870 | 1.667 |
| 34 | GCST90319878 | IVW | 3 | 0.100 | 0.109 | 0.357 | 1.105 | 0.893 | 1.368 |
| 35 | GCST90319878 | IVW | 2 | -0.016 | 0.154 | 0.915 | 0.984 | 0.728 | 1.329 |
| 40 | GCST90319878 | IVW | 2 | 0.255 | 0.162 | 0.117 | 1.290 | 0.938 | 1.773 |
| 60 | GCST90319878 | IVW | 2 | 0.068 | 0.263 | 0.795 | 1.071 | 0.639 | 1.794 |
| 61 | GCST90319878 | IVW | 3 | -0.013 | 0.113 | 0.907 | 0.987 | 0.790 | 1.232 |
| 63 | GCST90319878 | IVW | 3 | -0.076 | 0.115 | 0.510 | 0.927 | 0.739 | 1.162 |
| 66 | GCST90319878 | IVW | 2 | -0.023 | 0.136 | 0.864 | 0.977 | 0.748 | 1.276 |
| 70 | GCST90319878 | IVW | 2 | -0.127 | 0.137 | 0.355 | 0.881 | 0.674 | 1.152 |
| 72 | GCST90319878 | IVW | 14 | 0.054 | 0.046 | 0.242 | 1.055 | 0.964 | 1.155 |
| 73 | GCST90319878 | IVW | 16 | 0.086 | 0.094 | 0.359 | 1.090 | 0.907 | 1.310 |
| 78 | GCST90319878 | IVW | 5 | -0.190 | 0.162 | 0.241 | 0.827 | 0.602 | 1.136 |
| 79 | GCST90319878 | IVW | 7 | 0.079 | 0.140 | 0.574 | 1.082 | 0.823 | 1.423 |
| 80 | GCST90319878 | IVW | 4 | 0.116 | 0.103 | 0.259 | 1.123 | 0.918 | 1.374 |
| 86 | GCST90319878 | IVW | 2 | -0.046 | 0.127 | 0.717 | 0.955 | 0.744 | 1.226 |
| 90 | GCST90319878 | IVW | 5 | 0.062 | 0.208 | 0.767 | 1.064 | 0.708 | 1.598 |
| 91 | GCST90319878 | IVW | 4 | -0.176 | 0.144 | 0.222 | 0.839 | 0.633 | 1.112 |
| 93 | GCST90319878 | IVW | 2 | 0.169 | 0.235 | 0.472 | 1.185 | 0.747 | 1.879 |
| 94 | GCST90319878 | IVW | 2 | -0.070 | 0.130 | 0.589 | 0.932 | 0.722 | 1.203 |
| 95 | GCST90319878 | IVW | 3 | 0.011 | 0.122 | 0.928 | 1.011 | 0.796 | 1.285 |
| 97 | GCST90319878 | IVW | 5 | -0.083 | 0.087 | 0.340 | 0.920 | 0.776 | 1.092 |
| 100 | GCST90319878 | IVW | 4 | 0.039 | 0.231 | 0.865 | 1.040 | 0.661 | 1.636 |
| 104 | GCST90319878 | IVW | 3 | 0.200 | 0.125 | 0.108 | 1.221 | 0.957 | 1.559 |
| 106 | GCST90319878 | IVW | 3 | 0.050 | 0.119 | 0.674 | 1.052 | 0.832 | 1.329 |
| 107 | GCST90319878 | IVW | 2 | -0.101 | 0.115 | 0.379 | 0.904 | 0.722 | 1.132 |
| 111 | GCST90319878 | IVW | 2 | 0.055 | 0.163 | 0.738 | 1.056 | 0.767 | 1.455 |
| 113 | GCST90319878 | IVW | 2 | 0.183 | 0.393 | 0.642 | 1.201 | 0.556 | 2.593 |
| 116 | GCST90319878 | IVW | 3 | -0.140 | 0.135 | 0.297 | 0.869 | 0.668 | 1.131 |
| 118 | GCST90319878 | IVW | 6 | -0.057 | 0.129 | 0.662 | 0.945 | 0.734 | 1.217 |
| 119 | GCST90319878 | IVW | 2 | -0.098 | 0.122 | 0.423 | 0.907 | 0.714 | 1.152 |
| 120 | GCST90319878 | IVW | 3 | -0.112 | 0.178 | 0.530 | 0.894 | 0.631 | 1.268 |
| 121 | GCST90319878 | IVW | 6 | 0.132 | 0.085 | 0.121 | 1.141 | 0.966 | 1.348 |
| 122 | GCST90319878 | IVW | 9 | 0.010 | 0.098 | 0.921 | 1.010 | 0.833 | 1.223 |
| 123 | GCST90319878 | IVW | 10 | 0.053 | 0.071 | 0.453 | 1.055 | 0.917 | 1.213 |
| 124 | GCST90319878 | IVW | 9 | -0.010 | 0.066 | 0.879 | 0.990 | 0.870 | 1.126 |
| 125 | GCST90319878 | IVW | 8 | 0.143 | 0.074 | 0.053 | 1.154 | 0.998 | 1.334 |
| 126 | GCST90319878 | IVW | 10 | 0.068 | 0.119 | 0.568 | 1.071 | 0.847 | 1.353 |
| 127 | GCST90319878 | IVW | 11 | 0.067 | 0.117 | 0.565 | 1.070 | 0.851 | 1.345 |
| 128 | GCST90319878 | IVW | 2 | 0.211 | 0.121 | 0.080 | 1.235 | 0.975 | 1.564 |
| 129 | GCST90319878 | IVW | 2 | 0.287 | 0.147 | 0.051 | 1.333 | 0.999 | 1.778 |
| 130 | GCST90319878 | IVW | 8 | 0.025 | 0.063 | 0.687 | 1.026 | 0.907 | 1.159 |
| 131 | GCST90319878 | IVW | 7 | -0.061 | 0.066 | 0.356 | 0.941 | 0.826 | 1.071 |
| 132 | GCST90319878 | IVW | 3 | 0.112 | 0.117 | 0.339 | 1.118 | 0.889 | 1.407 |
| 133 | GCST90319878 | IVW | 2 | -0.014 | 0.331 | 0.965 | 0.986 | 0.515 | 1.885 |
| 134 | GCST90319878 | IVW | 10 | 0.047 | 0.081 | 0.566 | 1.048 | 0.893 | 1.229 |
| 135 | GCST90319878 | IVW | 5 | 0.137 | 0.122 | 0.262 | 1.147 | 0.902 | 1.458 |
| 137 | GCST90319878 | IVW | 2 | 0.183 | 0.193 | 0.342 | 1.201 | 0.823 | 1.753 |
| 322 | GCST90319878 | IVW | 2 | -0.708 | 0.281 | 0.012 | 0.493 | 0.284 | 0.855 |
| 139 | GCST90319878 | IVW | 5 | 0.114 | 0.094 | 0.225 | 1.120 | 0.932 | 1.346 |
| 140 | GCST90319878 | IVW | 6 | -0.004 | 0.079 | 0.961 | 0.996 | 0.854 | 1.162 |
| 141 | GCST90319878 | IVW | 2 | -0.559 | 0.654 | 0.393 | 0.572 | 0.159 | 2.061 |
| 142 | GCST90319878 | IVW | 10 | -0.096 | 0.093 | 0.299 | 0.908 | 0.758 | 1.089 |
| 143 | GCST90319878 | IVW | 4 | 0.054 | 0.101 | 0.595 | 1.055 | 0.866 | 1.286 |
| 144 | GCST90319878 | IVW | 10 | 0.028 | 0.060 | 0.642 | 1.028 | 0.914 | 1.158 |
| 146 | GCST90319878 | IVW | 17 | 0.042 | 0.048 | 0.391 | 1.042 | 0.948 | 1.146 |
| 147 | GCST90319878 | IVW | 15 | 0.009 | 0.045 | 0.837 | 1.009 | 0.924 | 1.102 |
| 148 | GCST90319878 | IVW | 10 | -0.018 | 0.066 | 0.783 | 0.982 | 0.863 | 1.117 |
| 149 | GCST90319878 | IVW | 16 | -0.027 | 0.045 | 0.539 | 0.973 | 0.891 | 1.062 |
| 150 | GCST90319878 | IVW | 11 | 0.052 | 0.051 | 0.307 | 1.054 | 0.953 | 1.165 |
| 151 | GCST90319878 | IVW | 4 | 0.052 | 0.100 | 0.602 | 1.053 | 0.866 | 1.281 |
| 152 | GCST90319878 | IVW | 12 | 0.025 | 0.049 | 0.615 | 1.025 | 0.930 | 1.130 |
| 153 | GCST90319878 | IVW | 14 | -0.049 | 0.101 | 0.625 | 0.952 | 0.782 | 1.160 |
| 154 | GCST90319878 | IVW | 12 | -0.013 | 0.112 | 0.905 | 0.987 | 0.792 | 1.229 |
| 155 | GCST90319878 | IVW | 9 | 0.069 | 0.077 | 0.369 | 1.072 | 0.922 | 1.246 |
| 156 | GCST90319878 | IVW | 5 | -0.289 | 0.251 | 0.249 | 0.749 | 0.459 | 1.224 |
| 157 | GCST90319878 | IVW | 11 | -0.003 | 0.056 | 0.964 | 0.997 | 0.894 | 1.113 |
| 158 | GCST90319878 | IVW | 3 | 0.468 | 0.378 | 0.216 | 1.596 | 0.761 | 3.350 |
| 159 | GCST90319878 | IVW | 10 | 0.216 | 0.145 | 0.135 | 1.242 | 0.935 | 1.648 |
| 160 | GCST90319878 | IVW | 5 | -0.292 | 0.262 | 0.266 | 0.747 | 0.446 | 1.249 |
| 161 | GCST90319878 | IVW | 5 | 0.401 | 0.232 | 0.084 | 1.493 | 0.948 | 2.352 |
| 162 | GCST90319878 | IVW | 3 | 0.023 | 0.112 | 0.840 | 1.023 | 0.822 | 1.273 |
| 163 | GCST90319878 | IVW | 5 | -0.065 | 0.088 | 0.458 | 0.937 | 0.788 | 1.113 |
| 164 | GCST90319878 | IVW | 3 | -0.084 | 0.124 | 0.495 | 0.919 | 0.721 | 1.171 |
| 165 | GCST90319878 | IVW | 4 | -0.132 | 0.181 | 0.466 | 0.877 | 0.615 | 1.249 |
| 166 | GCST90319878 | IVW | 4 | -0.056 | 0.205 | 0.785 | 0.946 | 0.633 | 1.412 |
| 167 | GCST90319878 | IVW | 5 | -0.037 | 0.145 | 0.797 | 0.963 | 0.725 | 1.280 |
| 168 | GCST90319878 | IVW | 3 | 0.070 | 0.119 | 0.557 | 1.072 | 0.850 | 1.352 |
| 170 | GCST90319878 | IVW | 7 | 0.007 | 0.122 | 0.953 | 1.007 | 0.793 | 1.279 |
| 171 | GCST90319878 | IVW | 5 | -0.047 | 0.158 | 0.768 | 0.954 | 0.700 | 1.301 |
| 172 | GCST90319878 | IVW | 2 | 0.047 | 0.088 | 0.596 | 1.048 | 0.882 | 1.244 |
| 173 | GCST90319878 | IVW | 6 | 0.055 | 0.070 | 0.434 | 1.056 | 0.921 | 1.211 |
| 174 | GCST90319878 | IVW | 7 | 0.035 | 0.119 | 0.768 | 1.036 | 0.820 | 1.309 |
| 175 | GCST90319878 | IVW | 11 | -0.034 | 0.069 | 0.627 | 0.967 | 0.845 | 1.107 |
| 176 | GCST90319878 | IVW | 2 | 0.041 | 0.029 | 0.149 | 1.042 | 0.985 | 1.102 |
| 177 | GCST90319878 | IVW | 17 | -0.115 | 0.063 | 0.070 | 0.891 | 0.787 | 1.009 |
| 178 | GCST90319878 | IVW | 4 | -0.071 | 0.078 | 0.361 | 0.931 | 0.800 | 1.085 |
| 179 | GCST90319878 | IVW | 6 | 0.038 | 0.102 | 0.711 | 1.038 | 0.851 | 1.267 |
| 181 | GCST90319878 | IVW | 3 | -0.009 | 0.141 | 0.947 | 0.991 | 0.751 | 1.306 |
| 182 | GCST90319878 | IVW | 10 | -0.048 | 0.071 | 0.498 | 0.953 | 0.829 | 1.095 |
| 183 | GCST90319878 | IVW | 5 | 0.073 | 0.145 | 0.614 | 1.076 | 0.810 | 1.429 |
| 184 | GCST90319878 | IVW | 5 | -0.168 | 0.144 | 0.245 | 0.845 | 0.637 | 1.122 |
| 185 | GCST90319878 | IVW | 3 | -0.254 | 0.185 | 0.170 | 0.776 | 0.540 | 1.115 |
| 186 | GCST90319878 | IVW | 7 | 0.004 | 0.077 | 0.963 | 1.004 | 0.864 | 1.166 |
| 187 | GCST90319878 | IVW | 9 | -0.052 | 0.058 | 0.373 | 0.950 | 0.848 | 1.064 |
| 188 | GCST90319878 | IVW | 3 | 0.287 | 0.250 | 0.251 | 1.332 | 0.817 | 2.172 |
| 190 | GCST90319878 | IVW | 12 | -0.021 | 0.070 | 0.769 | 0.980 | 0.854 | 1.124 |
| 191 | GCST90319878 | IVW | 6 | -0.030 | 0.065 | 0.642 | 0.970 | 0.853 | 1.103 |
| 192 | GCST90319878 | IVW | 3 | -0.058 | 0.091 | 0.524 | 0.944 | 0.789 | 1.128 |
| 193 | GCST90319878 | IVW | 8 | 0.093 | 0.067 | 0.167 | 1.097 | 0.962 | 1.251 |
| 194 | GCST90319878 | IVW | 10 | -0.049 | 0.064 | 0.443 | 0.952 | 0.840 | 1.079 |
| 196 | GCST90319878 | IVW | 9 | -0.025 | 0.065 | 0.704 | 0.976 | 0.858 | 1.109 |
| 197 | GCST90319878 | IVW | 8 | 0.058 | 0.105 | 0.583 | 1.059 | 0.862 | 1.301 |
| 198 | GCST90319878 | IVW | 3 | 0.117 | 0.099 | 0.239 | 1.124 | 0.925 | 1.366 |
| 199 | GCST90319878 | IVW | 8 | -0.030 | 0.056 | 0.585 | 0.970 | 0.870 | 1.082 |
| 201 | GCST90319878 | IVW | 2 | -0.134 | 0.124 | 0.279 | 0.875 | 0.687 | 1.114 |
| 202 | GCST90319878 | IVW | 9 | 0.120 | 0.065 | 0.065 | 1.127 | 0.993 | 1.281 |
| 204 | GCST90319878 | IVW | 4 | 0.104 | 0.103 | 0.311 | 1.110 | 0.907 | 1.357 |
| 205 | GCST90319878 | IVW | 3 | -0.130 | 0.169 | 0.442 | 0.878 | 0.631 | 1.222 |
| 207 | GCST90319878 | IVW | 12 | -0.022 | 0.073 | 0.759 | 0.978 | 0.848 | 1.128 |
| 208 | GCST90319878 | IVW | 6 | 0.073 | 0.072 | 0.316 | 1.075 | 0.933 | 1.240 |
| 210 | GCST90319878 | IVW | 7 | 0.090 | 0.068 | 0.188 | 1.094 | 0.957 | 1.250 |
| 211 | GCST90319878 | IVW | 12 | 0.043 | 0.061 | 0.476 | 1.044 | 0.927 | 1.176 |
| 212 | GCST90319878 | IVW | 4 | -0.077 | 0.094 | 0.414 | 0.926 | 0.771 | 1.113 |
| 213 | GCST90319878 | IVW | 5 | -0.017 | 0.088 | 0.848 | 0.983 | 0.828 | 1.168 |
| 214 | GCST90319878 | IVW | 10 | 0.130 | 0.086 | 0.130 | 1.139 | 0.962 | 1.348 |
| 215 | GCST90319878 | IVW | 3 | 0.179 | 0.148 | 0.227 | 1.196 | 0.895 | 1.599 |
| 216 | GCST90319878 | IVW | 7 | -0.051 | 0.063 | 0.418 | 0.950 | 0.839 | 1.076 |
| 217 | GCST90319878 | IVW | 4 | -0.095 | 0.096 | 0.321 | 0.910 | 0.754 | 1.097 |
| 218 | GCST90319878 | IVW | 3 | -0.075 | 0.163 | 0.646 | 0.928 | 0.674 | 1.278 |
| 219 | GCST90319878 | IVW | 10 | 0.116 | 0.066 | 0.080 | 1.123 | 0.986 | 1.278 |
| 221 | GCST90319878 | IVW | 5 | 0.111 | 0.094 | 0.238 | 1.117 | 0.929 | 1.343 |
| 223 | GCST90319878 | IVW | 7 | -0.051 | 0.069 | 0.458 | 0.950 | 0.831 | 1.087 |
| 224 | GCST90319878 | IVW | 7 | -0.016 | 0.080 | 0.840 | 0.984 | 0.841 | 1.151 |
| 226 | GCST90319878 | IVW | 3 | -0.022 | 0.110 | 0.839 | 0.978 | 0.789 | 1.212 |
| 230 | GCST90319878 | IVW | 2 | 0.040 | 0.142 | 0.777 | 1.041 | 0.789 | 1.374 |
| 231 | GCST90319878 | IVW | 3 | 0.057 | 0.100 | 0.567 | 1.059 | 0.870 | 1.288 |
| 232 | GCST90319878 | IVW | 5 | -0.006 | 0.092 | 0.946 | 0.994 | 0.830 | 1.190 |
| 233 | GCST90319878 | IVW | 9 | -0.002 | 0.064 | 0.980 | 0.998 | 0.881 | 1.131 |
| 234 | GCST90319878 | IVW | 5 | -0.080 | 0.168 | 0.633 | 0.923 | 0.663 | 1.284 |
| 235 | GCST90319878 | IVW | 2 | 0.252 | 0.154 | 0.102 | 1.286 | 0.951 | 1.739 |
| 236 | GCST90319878 | IVW | 3 | -0.062 | 0.187 | 0.742 | 0.940 | 0.652 | 1.356 |
| 621 | GCST90319878 | IVW | 2 | 0.378 | 0.148 | 0.011 | 1.460 | 1.091 | 1.953 |
| 241 | GCST90319878 | IVW | 3 | 0.020 | 0.108 | 0.854 | 1.020 | 0.825 | 1.261 |
| 242 | GCST90319878 | IVW | 5 | 0.025 | 0.095 | 0.794 | 1.025 | 0.850 | 1.236 |
| 243 | GCST90319878 | IVW | 9 | -0.042 | 0.062 | 0.495 | 0.959 | 0.850 | 1.082 |
| 244 | GCST90319878 | IVW | 7 | -0.006 | 0.066 | 0.925 | 0.994 | 0.873 | 1.131 |
| 245 | GCST90319878 | IVW | 5 | -0.103 | 0.092 | 0.261 | 0.902 | 0.753 | 1.080 |
| 246 | GCST90319878 | IVW | 5 | 0.027 | 0.118 | 0.818 | 1.028 | 0.815 | 1.295 |
| 247 | GCST90319878 | IVW | 3 | 0.047 | 0.145 | 0.744 | 1.048 | 0.789 | 1.393 |
| 249 | GCST90319878 | IVW | 7 | -0.037 | 0.063 | 0.559 | 0.964 | 0.852 | 1.090 |
| 250 | GCST90319878 | IVW | 5 | 0.027 | 0.086 | 0.751 | 1.028 | 0.869 | 1.215 |
| 252 | GCST90319878 | IVW | 3 | -0.029 | 0.115 | 0.798 | 0.971 | 0.775 | 1.216 |
| 253 | GCST90319878 | IVW | 4 | -0.028 | 0.124 | 0.820 | 0.972 | 0.763 | 1.239 |
| 254 | GCST90319878 | IVW | 6 | 0.014 | 0.066 | 0.830 | 1.014 | 0.891 | 1.155 |
| 848 | GCST90319878 | IVW | 2 | -0.496 | 0.204 | 0.015 | 0.609 | 0.408 | 0.908 |
| 256 | GCST90319878 | IVW | 5 | -0.045 | 0.082 | 0.582 | 0.956 | 0.815 | 1.122 |
| 257 | GCST90319878 | IVW | 5 | -0.074 | 0.070 | 0.290 | 0.929 | 0.811 | 1.065 |
| 258 | GCST90319878 | IVW | 7 | -0.030 | 0.069 | 0.668 | 0.971 | 0.848 | 1.111 |
| 260 | GCST90319878 | IVW | 4 | 0.015 | 0.140 | 0.916 | 1.015 | 0.771 | 1.335 |
| 261 | GCST90319878 | IVW | 4 | -0.031 | 0.119 | 0.793 | 0.969 | 0.768 | 1.224 |
| 262 | GCST90319878 | IVW | 10 | -0.018 | 0.055 | 0.745 | 0.982 | 0.882 | 1.094 |
| 263 | GCST90319878 | IVW | 9 | -0.052 | 0.053 | 0.333 | 0.950 | 0.856 | 1.054 |
| 264 | GCST90319878 | IVW | 8 | -0.008 | 0.053 | 0.882 | 0.992 | 0.893 | 1.102 |
| 265 | GCST90319878 | IVW | 11 | 0.006 | 0.051 | 0.905 | 1.006 | 0.911 | 1.112 |
| 266 | GCST90319878 | IVW | 9 | -0.006 | 0.057 | 0.923 | 0.994 | 0.890 | 1.111 |
| 267 | GCST90319878 | IVW | 6 | -0.058 | 0.091 | 0.529 | 0.944 | 0.789 | 1.129 |
| 268 | GCST90319878 | IVW | 6 | 0.062 | 0.096 | 0.521 | 1.063 | 0.881 | 1.284 |
| 271 | GCST90319878 | IVW | 5 | -0.031 | 0.090 | 0.730 | 0.969 | 0.812 | 1.157 |
| 272 | GCST90319878 | IVW | 2 | -0.016 | 0.136 | 0.907 | 0.984 | 0.753 | 1.286 |
| 274 | GCST90319878 | IVW | 6 | -0.053 | 0.081 | 0.514 | 0.948 | 0.809 | 1.112 |
| 276 | GCST90319878 | IVW | 6 | 0.002 | 0.071 | 0.983 | 1.002 | 0.871 | 1.152 |
| 277 | GCST90319878 | IVW | 5 | -0.124 | 0.086 | 0.148 | 0.883 | 0.747 | 1.045 |
| 278 | GCST90319878 | IVW | 7 | 0.043 | 0.074 | 0.556 | 1.044 | 0.904 | 1.207 |
| 279 | GCST90319878 | IVW | 7 | -0.018 | 0.069 | 0.793 | 0.982 | 0.857 | 1.125 |
| 280 | GCST90319878 | IVW | 7 | -0.036 | 0.071 | 0.616 | 0.965 | 0.840 | 1.109 |
| 282 | GCST90319878 | IVW | 4 | -0.052 | 0.081 | 0.521 | 0.949 | 0.809 | 1.113 |
| 284 | GCST90319878 | IVW | 8 | 0.032 | 0.060 | 0.592 | 1.033 | 0.918 | 1.161 |
| 285 | GCST90319878 | IVW | 6 | 0.040 | 0.078 | 0.611 | 1.041 | 0.893 | 1.213 |
| 286 | GCST90319878 | IVW | 7 | -0.035 | 0.056 | 0.533 | 0.966 | 0.866 | 1.077 |
| 289 | GCST90319878 | IVW | 2 | -0.133 | 0.140 | 0.341 | 0.875 | 0.665 | 1.152 |
| 290 | GCST90319878 | IVW | 2 | -0.103 | 0.141 | 0.465 | 0.902 | 0.685 | 1.189 |
| 291 | GCST90319878 | IVW | 2 | -0.083 | 0.195 | 0.669 | 0.920 | 0.627 | 1.349 |
| 292 | GCST90319878 | IVW | 2 | -0.079 | 0.157 | 0.614 | 0.924 | 0.678 | 1.257 |
| 293 | GCST90319878 | IVW | 7 | 0.042 | 0.080 | 0.600 | 1.043 | 0.891 | 1.220 |
| 294 | GCST90319878 | IVW | 5 | 0.028 | 0.098 | 0.777 | 1.028 | 0.849 | 1.245 |
| 295 | GCST90319878 | IVW | 3 | -0.140 | 0.097 | 0.147 | 0.869 | 0.719 | 1.050 |
| 296 | GCST90319878 | IVW | 2 | 0.141 | 0.134 | 0.290 | 1.152 | 0.886 | 1.497 |
| 297 | GCST90319878 | IVW | 4 | 0.114 | 0.144 | 0.427 | 1.121 | 0.846 | 1.486 |
| 300 | GCST90319878 | IVW | 3 | -0.011 | 0.111 | 0.921 | 0.989 | 0.795 | 1.230 |
| 302 | GCST90319878 | IVW | 3 | -0.171 | 0.141 | 0.224 | 0.843 | 0.640 | 1.110 |
| 303 | GCST90319878 | IVW | 3 | -0.307 | 0.217 | 0.158 | 0.736 | 0.481 | 1.127 |
| 304 | GCST90319878 | IVW | 3 | 0.015 | 0.124 | 0.905 | 1.015 | 0.796 | 1.294 |
| 306 | GCST90319878 | IVW | 2 | -0.013 | 0.199 | 0.947 | 0.987 | 0.668 | 1.458 |
| 307 | GCST90319878 | IVW | 2 | 0.179 | 0.262 | 0.494 | 1.196 | 0.716 | 2.000 |
| 308 | GCST90319878 | IVW | 3 | -0.005 | 0.138 | 0.971 | 0.995 | 0.760 | 1.303 |
| 309 | GCST90319878 | IVW | 5 | -0.061 | 0.096 | 0.526 | 0.941 | 0.779 | 1.136 |
| 310 | GCST90319878 | IVW | 2 | 0.099 | 0.130 | 0.444 | 1.104 | 0.856 | 1.425 |
| 313 | GCST90319878 | IVW | 4 | -0.163 | 0.108 | 0.129 | 0.849 | 0.688 | 1.048 |
| 315 | GCST90319878 | IVW | 3 | -0.166 | 0.100 | 0.096 | 0.847 | 0.696 | 1.030 |
| 316 | GCST90319878 | IVW | 2 | 0.029 | 0.156 | 0.851 | 1.030 | 0.759 | 1.397 |
| 317 | GCST90319878 | IVW | 6 | 0.013 | 0.097 | 0.894 | 1.013 | 0.837 | 1.226 |
| 318 | GCST90319878 | IVW | 4 | 0.078 | 0.097 | 0.424 | 1.081 | 0.894 | 1.307 |
| 320 | GCST90319878 | IVW | 3 | 0.165 | 0.121 | 0.175 | 1.179 | 0.929 | 1.496 |
| 321 | GCST90319878 | IVW | 6 | 0.138 | 0.128 | 0.280 | 1.148 | 0.893 | 1.476 |
| 691 | GCST90319878 | IVW | 2 | -0.483 | 0.205 | 0.018 | 0.617 | 0.412 | 0.922 |
| 324 | GCST90319878 | IVW | 4 | 0.050 | 0.202 | 0.804 | 1.051 | 0.707 | 1.563 |
| 325 | GCST90319878 | IVW | 2 | -0.132 | 0.196 | 0.502 | 0.877 | 0.597 | 1.287 |
| 327 | GCST90319878 | IVW | 3 | 0.139 | 0.118 | 0.237 | 1.150 | 0.913 | 1.448 |
| 330 | GCST90319878 | IVW | 2 | -0.026 | 0.136 | 0.849 | 0.975 | 0.747 | 1.271 |
| 331 | GCST90319878 | IVW | 3 | -0.100 | 0.117 | 0.390 | 0.904 | 0.719 | 1.137 |
| 332 | GCST90319878 | IVW | 2 | -0.052 | 0.134 | 0.698 | 0.949 | 0.729 | 1.235 |
| 333 | GCST90319878 | IVW | 2 | -0.031 | 0.144 | 0.831 | 0.970 | 0.732 | 1.285 |
| 334 | GCST90319878 | IVW | 7 | 0.050 | 0.082 | 0.541 | 1.051 | 0.896 | 1.234 |
| 335 | GCST90319878 | IVW | 3 | -0.017 | 0.116 | 0.886 | 0.983 | 0.783 | 1.235 |
| 336 | GCST90319878 | IVW | 4 | 0.073 | 0.124 | 0.556 | 1.076 | 0.843 | 1.373 |
| 337 | GCST90319878 | IVW | 3 | 0.035 | 0.106 | 0.745 | 1.035 | 0.840 | 1.275 |
| 338 | GCST90319878 | IVW | 2 | 0.015 | 0.133 | 0.913 | 1.015 | 0.782 | 1.317 |
| 339 | GCST90319878 | IVW | 9 | 0.029 | 0.061 | 0.639 | 1.029 | 0.913 | 1.159 |
| 340 | GCST90319878 | IVW | 23 | -0.067 | 0.049 | 0.165 | 0.935 | 0.850 | 1.028 |
| 341 | GCST90319878 | IVW | 3 | 0.279 | 0.148 | 0.060 | 1.321 | 0.988 | 1.767 |
| 342 | GCST90319878 | IVW | 8 | 0.029 | 0.094 | 0.754 | 1.030 | 0.857 | 1.238 |
| 343 | GCST90319878 | IVW | 20 | -0.073 | 0.060 | 0.224 | 0.930 | 0.827 | 1.045 |
| 346 | GCST90319878 | IVW | 2 | 0.259 | 0.290 | 0.373 | 1.295 | 0.733 | 2.288 |
| 347 | GCST90319878 | IVW | 11 | -0.027 | 0.048 | 0.574 | 0.973 | 0.886 | 1.069 |
| 353 | GCST90319878 | IVW | 3 | -0.030 | 0.117 | 0.798 | 0.970 | 0.771 | 1.221 |
| 354 | GCST90319878 | IVW | 2 | 0.137 | 0.178 | 0.443 | 1.147 | 0.808 | 1.627 |
| 355 | GCST90319878 | IVW | 8 | -0.141 | 0.088 | 0.110 | 0.868 | 0.730 | 1.033 |
| 360 | GCST90319878 | IVW | 2 | 0.022 | 0.121 | 0.858 | 1.022 | 0.806 | 1.295 |
| 361 | GCST90319878 | IVW | 2 | -0.208 | 0.242 | 0.389 | 0.812 | 0.506 | 1.304 |
| 362 | GCST90319878 | IVW | 4 | 0.055 | 0.092 | 0.549 | 1.057 | 0.882 | 1.266 |
| 363 | GCST90319878 | IVW | 13 | -0.068 | 0.055 | 0.217 | 0.934 | 0.839 | 1.041 |
| 367 | GCST90319878 | IVW | 3 | -0.026 | 0.109 | 0.813 | 0.975 | 0.787 | 1.206 |
| 371 | GCST90319878 | IVW | 5 | 0.071 | 0.098 | 0.470 | 1.073 | 0.886 | 1.299 |
| 373 | GCST90319878 | IVW | 2 | -0.053 | 0.115 | 0.644 | 0.948 | 0.756 | 1.188 |
| 380 | GCST90319878 | IVW | 7 | 0.005 | 0.062 | 0.934 | 1.005 | 0.890 | 1.135 |
| 388 | GCST90319878 | IVW | 7 | 0.067 | 0.094 | 0.471 | 1.070 | 0.891 | 1.285 |
| 394 | GCST90319878 | IVW | 2 | -0.251 | 0.163 | 0.122 | 0.778 | 0.565 | 1.070 |
| 395 | GCST90319878 | IVW | 3 | 0.010 | 0.132 | 0.937 | 1.011 | 0.780 | 1.310 |
| 396 | GCST90319878 | IVW | 13 | 0.060 | 0.055 | 0.274 | 1.062 | 0.954 | 1.182 |
| 397 | GCST90319878 | IVW | 2 | 0.056 | 0.064 | 0.378 | 1.058 | 0.933 | 1.199 |
| 399 | GCST90319878 | IVW | 2 | 0.110 | 0.134 | 0.413 | 1.116 | 0.858 | 1.452 |
| 400 | GCST90319878 | IVW | 4 | 0.116 | 0.091 | 0.204 | 1.123 | 0.939 | 1.343 |
| 402 | GCST90319878 | IVW | 2 | 0.159 | 0.132 | 0.227 | 1.173 | 0.906 | 1.519 |
| 404 | GCST90319878 | IVW | 4 | -0.114 | 0.097 | 0.241 | 0.892 | 0.737 | 1.080 |
| 406 | GCST90319878 | IVW | 3 | 0.026 | 0.078 | 0.743 | 1.026 | 0.880 | 1.196 |
| 410 | GCST90319878 | IVW | 2 | 0.047 | 0.063 | 0.454 | 1.048 | 0.927 | 1.185 |
| 412 | GCST90319878 | IVW | 2 | 0.018 | 0.161 | 0.913 | 1.018 | 0.743 | 1.395 |
| 413 | GCST90319878 | IVW | 2 | 0.043 | 0.051 | 0.399 | 1.044 | 0.945 | 1.154 |
| 418 | GCST90319878 | IVW | 4 | 0.077 | 0.070 | 0.271 | 1.080 | 0.942 | 1.238 |
| 419 | GCST90319878 | IVW | 10 | -0.096 | 0.059 | 0.102 | 0.908 | 0.809 | 1.019 |
| 420 | GCST90319878 | IVW | 8 | -0.024 | 0.093 | 0.798 | 0.977 | 0.815 | 1.171 |
| 421 | GCST90319878 | IVW | 4 | 0.013 | 0.025 | 0.608 | 1.013 | 0.965 | 1.063 |
| 423 | GCST90319878 | IVW | 2 | -0.244 | 0.152 | 0.108 | 0.784 | 0.582 | 1.055 |
| 424 | GCST90319878 | IVW | 2 | 0.034 | 0.074 | 0.642 | 1.035 | 0.895 | 1.197 |
| 425 | GCST90319878 | IVW | 3 | 0.055 | 0.099 | 0.581 | 1.056 | 0.870 | 1.282 |
| 429 | GCST90319878 | IVW | 2 | 0.137 | 0.147 | 0.353 | 1.147 | 0.859 | 1.530 |
| 430 | GCST90319878 | IVW | 3 | 0.203 | 0.127 | 0.111 | 1.225 | 0.954 | 1.572 |
| 432 | GCST90319878 | IVW | 3 | -0.003 | 0.135 | 0.984 | 0.997 | 0.766 | 1.299 |
| 433 | GCST90319878 | IVW | 8 | 0.111 | 0.064 | 0.084 | 1.117 | 0.985 | 1.267 |
| 434 | GCST90319878 | IVW | 9 | 0.024 | 0.061 | 0.702 | 1.024 | 0.908 | 1.155 |
| 435 | GCST90319878 | IVW | 4 | 0.063 | 0.102 | 0.540 | 1.065 | 0.871 | 1.301 |
| 437 | GCST90319878 | IVW | 3 | 0.026 | 0.151 | 0.865 | 1.026 | 0.763 | 1.380 |
| 730 | GCST90319878 | IVW | 2 | -0.462 | 0.143 | 0.001 | 0.630 | 0.476 | 0.834 |
| 439 | GCST90319878 | IVW | 2 | -0.298 | 0.160 | 0.063 | 0.742 | 0.542 | 1.016 |
| 440 | GCST90319878 | IVW | 9 | 0.006 | 0.056 | 0.920 | 1.006 | 0.902 | 1.122 |
| 446 | GCST90319878 | IVW | 2 | -0.017 | 0.147 | 0.907 | 0.983 | 0.737 | 1.310 |
| 447 | GCST90319878 | IVW | 4 | 0.064 | 0.096 | 0.503 | 1.067 | 0.883 | 1.288 |
| 448 | GCST90319878 | IVW | 8 | -0.122 | 0.085 | 0.151 | 0.885 | 0.749 | 1.046 |
| 453 | GCST90319878 | IVW | 2 | 0.016 | 0.131 | 0.905 | 1.016 | 0.786 | 1.313 |
| 454 | GCST90319878 | IVW | 2 | -0.119 | 0.215 | 0.580 | 0.887 | 0.582 | 1.354 |
| 455 | GCST90319878 | IVW | 5 | 0.057 | 0.067 | 0.394 | 1.059 | 0.928 | 1.208 |
| 456 | GCST90319878 | IVW | 14 | -0.074 | 0.054 | 0.166 | 0.928 | 0.836 | 1.031 |
| 457 | GCST90319878 | IVW | 2 | 0.067 | 0.063 | 0.293 | 1.069 | 0.944 | 1.210 |
| 460 | GCST90319878 | IVW | 4 | -0.060 | 0.093 | 0.519 | 0.942 | 0.786 | 1.130 |
| 237 | GCST90319878 | IVW | 2 | 0.352 | 0.153 | 0.021 | 1.421 | 1.054 | 1.918 |
| 464 | GCST90319878 | IVW | 5 | 0.064 | 0.096 | 0.505 | 1.066 | 0.884 | 1.285 |
| 466 | GCST90319878 | IVW | 2 | -0.054 | 0.110 | 0.627 | 0.948 | 0.764 | 1.176 |
| 468 | GCST90319878 | IVW | 4 | -0.073 | 0.116 | 0.531 | 0.930 | 0.740 | 1.168 |
| 471 | GCST90319878 | IVW | 8 | -0.041 | 0.061 | 0.502 | 0.960 | 0.851 | 1.082 |
| 478 | GCST90319878 | IVW | 2 | -0.033 | 0.154 | 0.829 | 0.967 | 0.715 | 1.309 |
| 479 | GCST90319878 | IVW | 7 | 0.068 | 0.094 | 0.465 | 1.071 | 0.891 | 1.286 |
| 480 | GCST90319878 | IVW | 2 | 0.047 | 0.153 | 0.758 | 1.048 | 0.777 | 1.414 |
| 482 | GCST90319878 | IVW | 2 | 0.047 | 0.139 | 0.736 | 1.048 | 0.798 | 1.376 |
| 485 | GCST90319878 | IVW | 3 | 0.032 | 0.107 | 0.767 | 1.032 | 0.837 | 1.272 |
| 486 | GCST90319878 | IVW | 3 | 0.104 | 0.111 | 0.349 | 1.110 | 0.892 | 1.380 |
| 487 | GCST90319878 | IVW | 13 | 0.061 | 0.055 | 0.268 | 1.063 | 0.954 | 1.183 |
| 488 | GCST90319878 | IVW | 2 | 0.061 | 0.069 | 0.375 | 1.063 | 0.929 | 1.216 |
| 490 | GCST90319878 | IVW | 2 | 0.106 | 0.134 | 0.429 | 1.112 | 0.855 | 1.446 |
| 491 | GCST90319878 | IVW | 4 | 0.111 | 0.091 | 0.222 | 1.118 | 0.935 | 1.336 |
| 493 | GCST90319878 | IVW | 4 | 0.014 | 0.122 | 0.906 | 1.014 | 0.799 | 1.287 |
| 495 | GCST90319878 | IVW | 5 | 0.087 | 0.084 | 0.303 | 1.091 | 0.925 | 1.286 |
| 497 | GCST90319878 | IVW | 3 | 0.029 | 0.078 | 0.712 | 1.029 | 0.883 | 1.199 |
| 499 | GCST90319878 | IVW | 6 | 0.101 | 0.077 | 0.192 | 1.106 | 0.951 | 1.287 |
| 509 | GCST90319878 | IVW | 4 | 0.030 | 0.100 | 0.764 | 1.030 | 0.847 | 1.253 |
| 510 | GCST90319878 | IVW | 11 | -0.084 | 0.051 | 0.098 | 0.920 | 0.833 | 1.016 |
| 513 | GCST90319878 | IVW | 3 | 0.108 | 0.099 | 0.277 | 1.114 | 0.917 | 1.353 |
| 519 | GCST90319878 | IVW | 2 | -0.241 | 0.138 | 0.081 | 0.786 | 0.600 | 1.030 |
| 521 | GCST90319878 | IVW | 5 | -0.068 | 0.132 | 0.608 | 0.935 | 0.721 | 1.211 |
| 522 | GCST90319878 | IVW | 2 | -0.226 | 0.133 | 0.088 | 0.798 | 0.615 | 1.034 |
| 525 | GCST90319878 | IVW | 2 | -0.054 | 0.112 | 0.629 | 0.948 | 0.761 | 1.179 |
| 526 | GCST90319878 | IVW | 5 | 0.066 | 0.106 | 0.537 | 1.068 | 0.867 | 1.316 |
| 527 | GCST90319878 | IVW | 2 | 0.072 | 0.098 | 0.462 | 1.075 | 0.887 | 1.302 |
| 529 | GCST90319878 | IVW | 5 | -0.088 | 0.084 | 0.293 | 0.916 | 0.777 | 1.079 |
| 540 | GCST90319878 | IVW | 3 | -0.147 | 0.232 | 0.526 | 0.863 | 0.548 | 1.359 |
| 542 | GCST90319878 | IVW | 2 | 0.091 | 0.143 | 0.528 | 1.095 | 0.826 | 1.450 |
| 543 | GCST90319878 | IVW | 12 | -0.018 | 0.077 | 0.814 | 0.982 | 0.845 | 1.141 |
| 548 | GCST90319878 | IVW | 4 | 0.178 | 0.102 | 0.080 | 1.195 | 0.979 | 1.459 |
| 551 | GCST90319878 | IVW | 2 | 0.234 | 0.147 | 0.112 | 1.263 | 0.947 | 1.685 |
| 556 | GCST90319878 | IVW | 2 | -0.102 | 0.286 | 0.721 | 0.903 | 0.516 | 1.581 |
| 560 | GCST90319878 | IVW | 2 | 0.061 | 0.145 | 0.672 | 1.063 | 0.800 | 1.412 |
| 561 | GCST90319878 | IVW | 2 | 0.149 | 0.175 | 0.396 | 1.160 | 0.824 | 1.634 |
| 563 | GCST90319878 | IVW | 2 | -0.065 | 0.142 | 0.650 | 0.937 | 0.709 | 1.239 |
| 564 | GCST90319878 | IVW | 4 | 0.043 | 0.161 | 0.790 | 1.044 | 0.761 | 1.432 |
| 566 | GCST90319878 | IVW | 4 | 0.095 | 0.092 | 0.300 | 1.100 | 0.919 | 1.316 |
| 570 | GCST90319878 | IVW | 2 | -0.038 | 0.095 | 0.688 | 0.962 | 0.798 | 1.160 |
| 572 | GCST90319878 | IVW | 4 | -0.175 | 0.127 | 0.169 | 0.840 | 0.655 | 1.077 |
| 583 | GCST90319878 | IVW | 4 | -0.192 | 0.128 | 0.134 | 0.825 | 0.641 | 1.061 |
| 584 | GCST90319878 | IVW | 8 | 0.009 | 0.063 | 0.889 | 1.009 | 0.892 | 1.141 |
| 593 | GCST90319878 | IVW | 2 | -0.279 | 0.191 | 0.143 | 0.756 | 0.521 | 1.099 |
| 595 | GCST90319878 | IVW | 6 | -0.003 | 0.108 | 0.976 | 0.997 | 0.807 | 1.231 |
| 598 | GCST90319878 | IVW | 2 | -0.064 | 0.209 | 0.758 | 0.938 | 0.622 | 1.413 |
| 599 | GCST90319878 | IVW | 4 | 0.003 | 0.080 | 0.969 | 1.003 | 0.858 | 1.172 |
| 600 | GCST90319878 | IVW | 3 | 0.006 | 0.118 | 0.958 | 1.006 | 0.799 | 1.268 |
| 601 | GCST90319878 | IVW | 2 | 0.056 | 0.063 | 0.371 | 1.058 | 0.935 | 1.197 |
| 603 | GCST90319878 | IVW | 3 | 0.087 | 0.099 | 0.378 | 1.091 | 0.899 | 1.324 |
| 615 | GCST90319878 | IVW | 4 | 0.146 | 0.103 | 0.155 | 1.157 | 0.946 | 1.415 |
| 617 | GCST90319878 | IVW | 14 | 0.032 | 0.057 | 0.580 | 1.032 | 0.923 | 1.154 |
| 618 | GCST90319878 | IVW | 2 | 0.078 | 0.098 | 0.426 | 1.081 | 0.892 | 1.311 |
| 620 | GCST90319878 | IVW | 2 | -0.128 | 0.136 | 0.347 | 0.880 | 0.674 | 1.148 |
| 1080 | GCST90319878 | IVW | 2 | 0.351 | 0.132 | 0.008 | 1.421 | 1.098 | 1.839 |
| 623 | GCST90319878 | IVW | 2 | -0.080 | 0.127 | 0.528 | 0.923 | 0.720 | 1.183 |
| 629 | GCST90319878 | IVW | 2 | -0.227 | 0.195 | 0.245 | 0.797 | 0.544 | 1.168 |
| 633 | GCST90319878 | IVW | 2 | -0.218 | 0.199 | 0.273 | 0.804 | 0.545 | 1.188 |
| 634 | GCST90319878 | IVW | 2 | 0.092 | 0.189 | 0.626 | 1.097 | 0.757 | 1.589 |
| 635 | GCST90319878 | IVW | 2 | -0.139 | 0.152 | 0.360 | 0.870 | 0.646 | 1.172 |
| 636 | GCST90319878 | IVW | 2 | -0.067 | 0.155 | 0.666 | 0.935 | 0.690 | 1.268 |
| 637 | GCST90319878 | IVW | 4 | 0.106 | 0.173 | 0.541 | 1.111 | 0.792 | 1.559 |
| 638 | GCST90319878 | IVW | 4 | -0.043 | 0.128 | 0.736 | 0.958 | 0.746 | 1.230 |
| 640 | GCST90319878 | IVW | 2 | 0.055 | 0.110 | 0.616 | 1.057 | 0.852 | 1.311 |
| 642 | GCST90319878 | IVW | 2 | 0.042 | 0.202 | 0.833 | 1.043 | 0.703 | 1.549 |
| 644 | GCST90319878 | IVW | 2 | -0.137 | 0.167 | 0.412 | 0.872 | 0.628 | 1.210 |
| 648 | GCST90319878 | IVW | 4 | -0.100 | 0.120 | 0.405 | 0.905 | 0.715 | 1.145 |
| 1054 | GCST90319878 | IVW | 3 | 0.336 | 0.117 | 0.004 | 1.399 | 1.112 | 1.760 |
| 652 | GCST90319878 | IVW | 7 | 0.045 | 0.060 | 0.448 | 1.046 | 0.931 | 1.176 |
| 653 | GCST90319878 | IVW | 2 | 0.137 | 0.120 | 0.256 | 1.146 | 0.905 | 1.451 |
| 658 | GCST90319878 | IVW | 6 | 0.065 | 0.108 | 0.546 | 1.068 | 0.863 | 1.320 |
| 659 | GCST90319878 | IVW | 3 | 0.119 | 0.108 | 0.268 | 1.127 | 0.912 | 1.391 |
| 660 | GCST90319878 | IVW | 10 | -0.020 | 0.075 | 0.785 | 0.980 | 0.846 | 1.135 |
| 666 | GCST90319878 | IVW | 3 | -0.110 | 0.131 | 0.398 | 0.895 | 0.693 | 1.157 |
| 667 | GCST90319878 | IVW | 5 | 0.074 | 0.065 | 0.253 | 1.077 | 0.949 | 1.222 |
| 668 | GCST90319878 | IVW | 17 | 0.027 | 0.044 | 0.539 | 1.027 | 0.943 | 1.119 |
| 669 | GCST90319878 | IVW | 5 | 0.071 | 0.096 | 0.458 | 1.074 | 0.889 | 1.296 |
| 670 | GCST90319878 | IVW | 2 | -0.091 | 0.135 | 0.499 | 0.913 | 0.700 | 1.189 |
| 671 | GCST90319878 | IVW | 2 | -0.129 | 0.159 | 0.419 | 0.879 | 0.644 | 1.201 |
| 672 | GCST90319878 | IVW | 2 | -0.022 | 0.190 | 0.906 | 0.978 | 0.674 | 1.419 |
| 674 | GCST90319878 | IVW | 2 | 0.143 | 0.177 | 0.420 | 1.154 | 0.815 | 1.633 |
| 675 | GCST90319878 | IVW | 3 | 0.114 | 0.160 | 0.475 | 1.121 | 0.819 | 1.534 |
| 676 | GCST90319878 | IVW | 6 | 0.051 | 0.072 | 0.476 | 1.052 | 0.915 | 1.211 |
| 811 | GCST90319878 | IVW | 3 | 0.317 | 0.117 | 0.007 | 1.373 | 1.091 | 1.727 |
| 678 | GCST90319878 | IVW | 4 | -0.003 | 0.100 | 0.973 | 0.997 | 0.820 | 1.211 |
| 680 | GCST90319878 | IVW | 3 | 0.136 | 0.230 | 0.554 | 1.146 | 0.730 | 1.800 |
| 810 | GCST90319878 | IVW | 2 | -0.433 | 0.141 | 0.002 | 0.649 | 0.492 | 0.856 |
| 685 | GCST90319878 | IVW | 2 | 0.249 | 0.195 | 0.200 | 1.283 | 0.876 | 1.880 |
| 686 | GCST90319878 | IVW | 10 | 0.081 | 0.075 | 0.278 | 1.084 | 0.937 | 1.256 |
| 689 | GCST90319878 | IVW | 4 | 0.034 | 0.219 | 0.876 | 1.035 | 0.673 | 1.591 |
| 438 | GCST90319878 | IVW | 2 | -0.411 | 0.145 | 0.004 | 0.663 | 0.499 | 0.880 |
| 692 | GCST90319878 | IVW | 6 | -0.073 | 0.082 | 0.375 | 0.930 | 0.792 | 1.092 |
| 693 | GCST90319878 | IVW | 2 | 0.061 | 0.122 | 0.617 | 1.063 | 0.837 | 1.350 |
| 694 | GCST90319878 | IVW | 10 | 0.005 | 0.065 | 0.938 | 1.005 | 0.885 | 1.142 |
| 695 | GCST90319878 | IVW | 2 | 0.269 | 0.154 | 0.081 | 1.308 | 0.967 | 1.770 |
| 697 | GCST90319878 | IVW | 3 | -0.053 | 0.109 | 0.627 | 0.948 | 0.766 | 1.175 |
| 700 | GCST90319878 | IVW | 3 | 0.002 | 0.095 | 0.984 | 1.002 | 0.831 | 1.208 |
| 701 | GCST90319878 | IVW | 4 | 0.076 | 0.132 | 0.563 | 1.079 | 0.834 | 1.397 |
| 702 | GCST90319878 | IVW | 17 | -0.022 | 0.043 | 0.605 | 0.978 | 0.900 | 1.063 |
| 703 | GCST90319878 | IVW | 2 | 0.080 | 0.080 | 0.317 | 1.084 | 0.926 | 1.269 |
| 704 | GCST90319878 | IVW | 4 | -0.170 | 0.099 | 0.086 | 0.844 | 0.695 | 1.024 |
| 705 | GCST90319878 | IVW | 2 | 0.066 | 0.065 | 0.315 | 1.068 | 0.939 | 1.214 |
| 706 | GCST90319878 | IVW | 6 | 0.085 | 0.103 | 0.410 | 1.088 | 0.890 | 1.332 |
| 708 | GCST90319878 | IVW | 2 | -0.141 | 0.251 | 0.575 | 0.869 | 0.531 | 1.421 |
| 709 | GCST90319878 | IVW | 2 | 0.102 | 0.368 | 0.781 | 1.108 | 0.539 | 2.278 |
| 710 | GCST90319878 | IVW | 4 | -0.154 | 0.088 | 0.082 | 0.858 | 0.721 | 1.020 |
| 711 | GCST90319878 | IVW | 2 | -0.364 | 0.146 | 0.013 | 0.695 | 0.522 | 0.925 |
| 712 | GCST90319878 | IVW | 3 | -0.001 | 0.083 | 0.992 | 0.999 | 0.850 | 1.175 |
| 716 | GCST90319878 | IVW | 4 | -0.049 | 0.119 | 0.680 | 0.952 | 0.754 | 1.202 |
| 719 | GCST90319878 | IVW | 3 | -0.062 | 0.242 | 0.796 | 0.939 | 0.585 | 1.508 |
| 720 | GCST90319878 | IVW | 11 | -0.010 | 0.049 | 0.832 | 0.990 | 0.898 | 1.090 |
| 726 | GCST90319878 | IVW | 7 | 0.136 | 0.120 | 0.257 | 1.146 | 0.906 | 1.449 |
| 682 | GCST90319878 | IVW | 4 | -0.288 | 0.096 | 0.003 | 0.750 | 0.621 | 0.904 |
| 728 | GCST90319878 | IVW | 8 | -0.019 | 0.114 | 0.870 | 0.981 | 0.785 | 1.228 |
| 729 | GCST90319878 | IVW | 2 | 0.157 | 0.188 | 0.405 | 1.170 | 0.809 | 1.692 |
| 786 | GCST90319878 | IVW | 2 | -0.260 | 0.129 | 0.043 | 0.771 | 0.599 | 0.992 |
| 735 | GCST90319878 | IVW | 4 | -0.059 | 0.072 | 0.414 | 0.943 | 0.819 | 1.086 |
| 736 | GCST90319878 | IVW | 13 | -0.003 | 0.057 | 0.956 | 0.997 | 0.892 | 1.114 |
| 737 | GCST90319878 | IVW | 3 | 0.061 | 0.069 | 0.375 | 1.063 | 0.929 | 1.217 |
| 739 | GCST90319878 | IVW | 3 | -0.050 | 0.125 | 0.691 | 0.951 | 0.745 | 1.216 |
| 740 | GCST90319878 | IVW | 3 | 0.055 | 0.106 | 0.603 | 1.057 | 0.859 | 1.300 |
| 742 | GCST90319878 | IVW | 3 | 0.070 | 0.125 | 0.577 | 1.072 | 0.839 | 1.371 |
| 743 | GCST90319878 | IVW | 4 | 0.109 | 0.122 | 0.375 | 1.115 | 0.877 | 1.417 |
| 744 | GCST90319878 | IVW | 4 | 0.025 | 0.101 | 0.803 | 1.025 | 0.842 | 1.249 |
| 745 | GCST90319878 | IVW | 2 | 0.051 | 0.144 | 0.724 | 1.052 | 0.793 | 1.397 |
| 746 | GCST90319878 | IVW | 3 | 0.070 | 0.101 | 0.490 | 1.073 | 0.879 | 1.308 |
| 748 | GCST90319878 | IVW | 2 | 0.095 | 0.155 | 0.541 | 1.100 | 0.811 | 1.491 |
| 749 | GCST90319878 | IVW | 5 | -0.141 | 0.119 | 0.233 | 0.868 | 0.688 | 1.095 |
| 752 | GCST90319878 | IVW | 3 | 0.171 | 0.142 | 0.230 | 1.187 | 0.898 | 1.569 |
| 753 | GCST90319878 | IVW | 11 | 0.102 | 0.097 | 0.291 | 1.108 | 0.916 | 1.339 |
| 754 | GCST90319878 | IVW | 2 | -0.255 | 0.169 | 0.130 | 0.775 | 0.557 | 1.078 |
| 756 | GCST90319878 | IVW | 3 | -0.010 | 0.138 | 0.940 | 0.990 | 0.755 | 1.298 |
| 759 | GCST90319878 | IVW | 6 | -0.095 | 0.101 | 0.349 | 0.909 | 0.745 | 1.109 |
| 760 | GCST90319878 | IVW | 4 | 0.163 | 0.231 | 0.481 | 1.177 | 0.748 | 1.850 |
| 761 | GCST90319878 | IVW | 10 | 0.036 | 0.073 | 0.625 | 1.036 | 0.898 | 1.196 |
| 764 | GCST90319878 | IVW | 3 | -0.009 | 0.112 | 0.937 | 0.991 | 0.796 | 1.234 |
| 767 | GCST90319878 | IVW | 2 | -0.084 | 0.164 | 0.609 | 0.920 | 0.667 | 1.268 |
| 768 | GCST90319878 | IVW | 5 | 0.063 | 0.101 | 0.533 | 1.065 | 0.874 | 1.297 |
| 769 | GCST90319878 | IVW | 17 | 0.073 | 0.070 | 0.300 | 1.076 | 0.937 | 1.235 |
| 770 | GCST90319878 | IVW | 2 | 0.064 | 0.072 | 0.374 | 1.066 | 0.926 | 1.228 |
| 772 | GCST90319878 | IVW | 2 | 0.063 | 0.064 | 0.325 | 1.065 | 0.939 | 1.207 |
| 773 | GCST90319878 | IVW | 2 | 0.171 | 0.175 | 0.329 | 1.187 | 0.842 | 1.674 |
| 775 | GCST90319878 | IVW | 2 | 0.049 | 0.190 | 0.796 | 1.050 | 0.724 | 1.525 |
| 776 | GCST90319878 | IVW | 2 | 0.243 | 0.209 | 0.245 | 1.275 | 0.847 | 1.919 |
| 777 | GCST90319878 | IVW | 5 | -0.026 | 0.080 | 0.748 | 0.975 | 0.833 | 1.140 |
| 779 | GCST90319878 | IVW | 4 | 0.017 | 0.100 | 0.862 | 1.018 | 0.836 | 1.238 |
| 782 | GCST90319878 | IVW | 2 | -0.017 | 0.110 | 0.876 | 0.983 | 0.792 | 1.220 |
| 783 | GCST90319878 | IVW | 3 | 0.027 | 0.086 | 0.752 | 1.027 | 0.868 | 1.216 |
| 784 | GCST90319878 | IVW | 2 | 0.054 | 0.067 | 0.426 | 1.055 | 0.925 | 1.204 |
| 785 | GCST90319878 | IVW | 4 | 0.009 | 0.077 | 0.911 | 1.009 | 0.867 | 1.174 |
| 727 | GCST90319878 | IVW | 3 | -0.255 | 0.126 | 0.043 | 0.775 | 0.606 | 0.992 |
| 787 | GCST90319878 | IVW | 3 | 0.079 | 0.109 | 0.467 | 1.083 | 0.874 | 1.341 |
| 788 | GCST90319878 | IVW | 3 | 0.233 | 0.122 | 0.058 | 1.262 | 0.993 | 1.604 |
| 790 | GCST90319878 | IVW | 4 | -0.017 | 0.079 | 0.831 | 0.983 | 0.843 | 1.147 |
| 791 | GCST90319878 | IVW | 19 | -0.021 | 0.042 | 0.613 | 0.979 | 0.901 | 1.063 |
| 792 | GCST90319878 | IVW | 12 | 0.015 | 0.061 | 0.803 | 1.015 | 0.901 | 1.145 |
| 793 | GCST90319878 | IVW | 2 | -0.199 | 0.184 | 0.278 | 0.820 | 0.572 | 1.174 |
| 794 | GCST90319878 | IVW | 2 | -0.077 | 0.188 | 0.680 | 0.926 | 0.641 | 1.337 |
| 795 | GCST90319878 | IVW | 2 | 0.020 | 0.120 | 0.869 | 1.020 | 0.806 | 1.290 |
| 796 | GCST90319878 | IVW | 2 | 0.102 | 0.107 | 0.337 | 1.108 | 0.899 | 1.366 |
| 797 | GCST90319878 | IVW | 2 | 0.045 | 0.100 | 0.655 | 1.046 | 0.859 | 1.273 |
| 799 | GCST90319878 | IVW | 3 | 0.023 | 0.061 | 0.703 | 1.024 | 0.908 | 1.155 |
| 801 | GCST90319878 | IVW | 4 | 0.120 | 0.075 | 0.108 | 1.128 | 0.974 | 1.306 |
| 802 | GCST90319878 | IVW | 5 | 0.043 | 0.139 | 0.760 | 1.043 | 0.794 | 1.371 |
| 804 | GCST90319878 | IVW | 3 | 0.089 | 0.107 | 0.405 | 1.093 | 0.887 | 1.347 |
| 805 | GCST90319878 | IVW | 17 | 0.008 | 0.045 | 0.856 | 1.008 | 0.923 | 1.101 |
| 806 | GCST90319878 | IVW | 12 | -0.031 | 0.056 | 0.582 | 0.970 | 0.868 | 1.083 |
| 807 | GCST90319878 | IVW | 3 | -0.116 | 0.099 | 0.242 | 0.891 | 0.734 | 1.081 |
| 808 | GCST90319878 | IVW | 4 | -0.003 | 0.118 | 0.978 | 0.997 | 0.791 | 1.256 |
| 809 | GCST90319878 | IVW | 2 | -0.061 | 0.141 | 0.667 | 0.941 | 0.714 | 1.240 |
| 138 | GCST90319878 | IVW | 5 | -0.223 | 0.092 | 0.016 | 0.800 | 0.668 | 0.959 |
| 651 | GCST90319878 | IVW | 3 | 0.317 | 0.117 | 0.007 | 1.373 | 1.091 | 1.727 |
| 812 | GCST90319878 | IVW | 5 | 0.063 | 0.081 | 0.436 | 1.065 | 0.909 | 1.249 |
| 818 | GCST90319878 | IVW | 6 | -0.009 | 0.076 | 0.903 | 0.991 | 0.853 | 1.150 |
| 819 | GCST90319878 | IVW | 4 | 0.006 | 0.092 | 0.945 | 1.006 | 0.840 | 1.206 |
| 820 | GCST90319878 | IVW | 11 | -0.006 | 0.071 | 0.927 | 0.994 | 0.865 | 1.142 |
| 822 | GCST90319878 | IVW | 4 | -0.182 | 0.098 | 0.063 | 0.834 | 0.688 | 1.010 |
| 823 | GCST90319878 | IVW | 3 | -0.171 | 0.125 | 0.173 | 0.843 | 0.659 | 1.078 |
| 824 | GCST90319878 | IVW | 2 | -0.236 | 0.657 | 0.719 | 0.789 | 0.218 | 2.861 |
| 827 | GCST90319878 | IVW | 6 | -0.100 | 0.060 | 0.098 | 0.905 | 0.804 | 1.019 |
| 828 | GCST90319878 | IVW | 18 | 0.009 | 0.042 | 0.839 | 1.009 | 0.928 | 1.096 |
| 829 | GCST90319878 | IVW | 6 | 0.091 | 0.093 | 0.327 | 1.095 | 0.913 | 1.314 |
| 830 | GCST90319878 | IVW | 2 | -0.144 | 0.136 | 0.292 | 0.866 | 0.663 | 1.131 |
| 831 | GCST90319878 | IVW | 2 | -0.127 | 0.160 | 0.427 | 0.881 | 0.643 | 1.206 |
| 832 | GCST90319878 | IVW | 3 | -0.038 | 0.111 | 0.733 | 0.963 | 0.775 | 1.196 |
| 834 | GCST90319878 | IVW | 3 | -0.035 | 0.224 | 0.877 | 0.966 | 0.623 | 1.497 |
| 835 | GCST90319878 | IVW | 4 | 0.077 | 0.150 | 0.609 | 1.080 | 0.805 | 1.449 |
| 836 | GCST90319878 | IVW | 6 | 0.000 | 0.074 | 0.998 | 1.000 | 0.864 | 1.156 |
| 837 | GCST90319878 | IVW | 2 | 0.229 | 0.165 | 0.163 | 1.258 | 0.911 | 1.737 |
| 838 | GCST90319878 | IVW | 3 | 0.003 | 0.135 | 0.985 | 1.003 | 0.770 | 1.306 |
| 839 | GCST90319878 | IVW | 3 | 0.134 | 0.226 | 0.555 | 1.143 | 0.734 | 1.781 |
| 840 | GCST90319878 | IVW | 6 | -0.060 | 0.079 | 0.451 | 0.942 | 0.806 | 1.100 |
| 842 | GCST90319878 | IVW | 2 | 0.249 | 0.185 | 0.178 | 1.283 | 0.892 | 1.846 |
| 843 | GCST90319878 | IVW | 9 | 0.064 | 0.085 | 0.450 | 1.066 | 0.903 | 1.260 |
| 846 | GCST90319878 | IVW | 3 | 0.082 | 0.205 | 0.690 | 1.085 | 0.726 | 1.622 |
| 847 | GCST90319878 | IVW | 2 | -0.223 | 0.134 | 0.095 | 0.800 | 0.615 | 1.040 |
| 255 | GCST90319878 | IVW | 6 | -0.153 | 0.076 | 0.044 | 0.858 | 0.739 | 0.996 |
| 849 | GCST90319878 | IVW | 6 | -0.070 | 0.081 | 0.382 | 0.932 | 0.796 | 1.091 |
| 850 | GCST90319878 | IVW | 3 | 0.188 | 0.284 | 0.508 | 1.207 | 0.691 | 2.107 |
| 851 | GCST90319878 | IVW | 9 | 0.035 | 0.059 | 0.553 | 1.036 | 0.922 | 1.164 |
| 852 | GCST90319878 | IVW | 2 | 0.001 | 0.152 | 0.997 | 1.001 | 0.743 | 1.347 |
| 853 | GCST90319878 | IVW | 3 | -0.086 | 0.191 | 0.653 | 0.918 | 0.632 | 1.334 |
| 854 | GCST90319878 | IVW | 2 | -0.017 | 0.123 | 0.893 | 0.984 | 0.774 | 1.251 |
| 855 | GCST90319878 | IVW | 3 | 0.121 | 0.116 | 0.295 | 1.129 | 0.900 | 1.416 |
| 858 | GCST90319878 | IVW | 3 | 0.095 | 0.103 | 0.357 | 1.100 | 0.898 | 1.347 |
| 859 | GCST90319878 | IVW | 17 | -0.019 | 0.042 | 0.652 | 0.981 | 0.903 | 1.066 |
| 860 | GCST90319878 | IVW | 3 | -0.029 | 0.079 | 0.711 | 0.971 | 0.831 | 1.135 |
| 861 | GCST90319878 | IVW | 5 | -0.155 | 0.088 | 0.079 | 0.857 | 0.720 | 1.018 |
| 862 | GCST90319878 | IVW | 2 | 0.063 | 0.063 | 0.318 | 1.065 | 0.941 | 1.206 |
| 863 | GCST90319878 | IVW | 6 | 0.051 | 0.124 | 0.677 | 1.053 | 0.826 | 1.342 |
| 865 | GCST90319878 | IVW | 2 | -0.210 | 0.139 | 0.131 | 0.811 | 0.618 | 1.064 |
| 867 | GCST90319878 | IVW | 6 | -0.138 | 0.079 | 0.078 | 0.871 | 0.746 | 1.016 |
| 868 | GCST90319878 | IVW | 3 | -0.099 | 0.117 | 0.397 | 0.906 | 0.720 | 1.140 |
| 869 | GCST90319878 | IVW | 3 | -0.004 | 0.082 | 0.957 | 0.996 | 0.848 | 1.169 |
| 870 | GCST90319878 | IVW | 3 | -0.103 | 0.118 | 0.384 | 0.903 | 0.717 | 1.137 |
| 871 | GCST90319878 | IVW | 5 | -0.020 | 0.103 | 0.845 | 0.980 | 0.801 | 1.200 |
| 873 | GCST90319878 | IVW | 2 | -0.210 | 0.149 | 0.158 | 0.811 | 0.606 | 1.085 |
| 874 | GCST90319878 | IVW | 2 | 0.331 | 0.368 | 0.369 | 1.392 | 0.676 | 2.864 |
| 875 | GCST90319878 | IVW | 3 | -0.043 | 0.086 | 0.619 | 0.958 | 0.810 | 1.133 |
| 878 | GCST90319878 | IVW | 2 | 0.197 | 0.332 | 0.552 | 1.218 | 0.636 | 2.332 |
| 879 | GCST90319878 | IVW | 2 | -0.170 | 0.148 | 0.251 | 0.843 | 0.631 | 1.128 |
| 881 | GCST90319878 | IVW | 3 | -0.075 | 0.130 | 0.565 | 0.928 | 0.719 | 1.197 |
| 882 | GCST90319878 | IVW | 9 | 0.010 | 0.058 | 0.864 | 1.010 | 0.901 | 1.132 |
| 885 | GCST90319878 | IVW | 4 | 0.081 | 0.079 | 0.305 | 1.085 | 0.929 | 1.266 |
| 887 | GCST90319878 | IVW | 5 | -0.062 | 0.157 | 0.694 | 0.940 | 0.691 | 1.279 |
| 889 | GCST90319878 | IVW | 3 | 0.137 | 0.106 | 0.196 | 1.147 | 0.932 | 1.411 |
| 893 | GCST90319878 | IVW | 8 | -0.077 | 0.098 | 0.429 | 0.926 | 0.764 | 1.121 |
| 895 | GCST90319878 | IVW | 2 | -0.028 | 0.156 | 0.856 | 0.972 | 0.716 | 1.320 |
| 897 | GCST90319878 | IVW | 3 | 0.012 | 0.110 | 0.912 | 1.012 | 0.816 | 1.256 |
| 898 | GCST90319878 | IVW | 3 | 0.049 | 0.152 | 0.749 | 1.050 | 0.780 | 1.413 |
| 899 | GCST90319878 | IVW | 2 | 0.088 | 0.104 | 0.395 | 1.092 | 0.891 | 1.338 |
| 900 | GCST90319878 | IVW | 3 | -0.208 | 0.121 | 0.087 | 0.812 | 0.640 | 1.030 |
| 901 | GCST90319878 | IVW | 2 | 0.039 | 0.109 | 0.723 | 1.039 | 0.840 | 1.286 |
| 911 | GCST90319878 | IVW | 2 | -0.034 | 0.131 | 0.792 | 0.966 | 0.748 | 1.248 |
| 912 | GCST90319878 | IVW | 3 | -0.198 | 0.120 | 0.098 | 0.820 | 0.649 | 1.037 |
| 913 | GCST90319878 | IVW | 6 | -0.163 | 0.090 | 0.070 | 0.849 | 0.712 | 1.013 |
| 915 | GCST90319878 | IVW | 17 | 0.034 | 0.047 | 0.466 | 1.035 | 0.943 | 1.136 |
| 919 | GCST90319878 | IVW | 2 | -0.084 | 0.209 | 0.688 | 0.919 | 0.610 | 1.386 |
| 920 | GCST90319878 | IVW | 5 | 0.155 | 0.085 | 0.069 | 1.168 | 0.988 | 1.381 |
| 921 | GCST90319878 | IVW | 2 | 0.182 | 0.157 | 0.248 | 1.199 | 0.881 | 1.632 |
| 925 | GCST90319878 | IVW | 2 | 0.161 | 0.234 | 0.493 | 1.174 | 0.742 | 1.859 |
| 928 | GCST90319878 | IVW | 3 | -0.104 | 0.141 | 0.462 | 0.901 | 0.683 | 1.189 |
| 934 | GCST90319878 | IVW | 4 | -0.095 | 0.093 | 0.305 | 0.909 | 0.758 | 1.091 |
| 935 | GCST90319878 | IVW | 5 | 0.018 | 0.094 | 0.850 | 1.018 | 0.847 | 1.224 |
| 936 | GCST90319878 | IVW | 4 | 0.036 | 0.165 | 0.826 | 1.037 | 0.751 | 1.432 |
| 938 | GCST90319878 | IVW | 4 | 0.049 | 0.078 | 0.532 | 1.050 | 0.901 | 1.223 |
| 942 | GCST90319878 | IVW | 3 | 0.105 | 0.090 | 0.244 | 1.111 | 0.931 | 1.325 |
| 944 | GCST90319878 | IVW | 5 | -0.064 | 0.101 | 0.525 | 0.938 | 0.769 | 1.144 |
| 677 | GCST90319878 | IVW | 2 | 0.310 | 0.144 | 0.032 | 1.363 | 1.028 | 1.807 |
| 956 | GCST90319878 | IVW | 15 | 0.102 | 0.075 | 0.174 | 1.108 | 0.956 | 1.284 |
| 958 | GCST90319878 | IVW | 2 | -0.117 | 0.140 | 0.405 | 0.890 | 0.676 | 1.171 |
| 959 | GCST90319878 | IVW | 3 | 0.021 | 0.170 | 0.904 | 1.021 | 0.732 | 1.424 |
| 964 | GCST90319878 | IVW | 2 | -0.028 | 0.323 | 0.931 | 0.972 | 0.516 | 1.833 |
| 965 | GCST90319878 | IVW | 4 | -0.050 | 0.127 | 0.691 | 0.951 | 0.741 | 1.220 |
| 967 | GCST90319878 | IVW | 7 | 0.033 | 0.079 | 0.672 | 1.034 | 0.886 | 1.207 |
| 970 | GCST90319878 | IVW | 2 | -0.214 | 0.176 | 0.225 | 0.807 | 0.572 | 1.140 |
| 971 | GCST90319878 | IVW | 3 | 0.109 | 0.089 | 0.220 | 1.115 | 0.937 | 1.328 |
| 972 | GCST90319878 | IVW | 3 | 0.028 | 0.098 | 0.775 | 1.028 | 0.849 | 1.246 |
| 973 | GCST90319878 | IVW | 2 | -0.017 | 0.106 | 0.875 | 0.984 | 0.800 | 1.210 |
| 974 | GCST90319878 | IVW | 2 | 0.079 | 0.076 | 0.302 | 1.082 | 0.932 | 1.256 |
| 975 | GCST90319878 | IVW | 4 | -0.109 | 0.161 | 0.496 | 0.896 | 0.654 | 1.229 |
| 979 | GCST90319878 | IVW | 2 | -0.320 | 0.225 | 0.155 | 0.726 | 0.467 | 1.129 |
| 986 | GCST90319878 | IVW | 3 | -0.235 | 0.121 | 0.052 | 0.790 | 0.623 | 1.002 |
| 987 | GCST90319878 | IVW | 10 | 0.016 | 0.061 | 0.793 | 1.016 | 0.902 | 1.144 |
| 988 | GCST90319878 | IVW | 2 | -0.085 | 0.151 | 0.573 | 0.919 | 0.684 | 1.234 |
| 989 | GCST90319878 | IVW | 13 | -0.006 | 0.062 | 0.922 | 0.994 | 0.881 | 1.122 |
| 990 | GCST90319878 | IVW | 3 | 0.048 | 0.090 | 0.593 | 1.049 | 0.880 | 1.252 |
| 994 | GCST90319878 | IVW | 2 | 0.160 | 0.223 | 0.473 | 1.173 | 0.758 | 1.814 |
| 995 | GCST90319878 | IVW | 3 | -0.085 | 0.093 | 0.359 | 0.918 | 0.765 | 1.102 |
| 997 | GCST90319878 | IVW | 2 | 0.055 | 0.134 | 0.681 | 1.057 | 0.813 | 1.373 |
| 1001 | GCST90319878 | IVW | 2 | -0.208 | 0.174 | 0.232 | 0.812 | 0.577 | 1.143 |
| 1002 | GCST90319878 | IVW | 2 | 0.089 | 0.132 | 0.499 | 1.093 | 0.844 | 1.416 |
| 1005 | GCST90319878 | IVW | 2 | -0.262 | 0.192 | 0.173 | 0.770 | 0.528 | 1.122 |
| 1006 | GCST90319878 | IVW | 3 | -0.016 | 0.102 | 0.879 | 0.985 | 0.806 | 1.203 |
| 1008 | GCST90319878 | IVW | 3 | 0.086 | 0.299 | 0.773 | 1.090 | 0.607 | 1.960 |
| 1009 | GCST90319878 | IVW | 2 | -0.104 | 0.257 | 0.685 | 0.901 | 0.544 | 1.491 |
| 1010 | GCST90319878 | IVW | 5 | 0.110 | 0.133 | 0.410 | 1.116 | 0.860 | 1.449 |
| 1012 | GCST90319878 | IVW | 3 | 0.025 | 0.089 | 0.776 | 1.026 | 0.861 | 1.222 |
| 1016 | GCST90319878 | IVW | 3 | 0.031 | 0.150 | 0.839 | 1.031 | 0.768 | 1.385 |
| 1020 | GCST90319878 | IVW | 3 | 0.237 | 0.134 | 0.076 | 1.267 | 0.975 | 1.646 |
| 1023 | GCST90319878 | IVW | 2 | -0.079 | 0.137 | 0.564 | 0.924 | 0.707 | 1.208 |
| 1027 | GCST90319878 | IVW | 4 | 0.019 | 0.104 | 0.852 | 1.020 | 0.831 | 1.251 |
| 1029 | GCST90319878 | IVW | 2 | -0.007 | 0.262 | 0.980 | 0.993 | 0.594 | 1.660 |
| 1036 | GCST90319878 | IVW | 2 | -0.143 | 0.251 | 0.568 | 0.867 | 0.530 | 1.418 |
| 1039 | GCST90319878 | IVW | 3 | -0.115 | 0.399 | 0.774 | 0.892 | 0.408 | 1.950 |
| 1044 | GCST90319878 | IVW | 4 | -0.124 | 0.158 | 0.431 | 0.883 | 0.648 | 1.204 |
| 1047 | GCST90319878 | IVW | 4 | 0.087 | 0.148 | 0.559 | 1.090 | 0.815 | 1.458 |
| 1048 | GCST90319878 | IVW | 5 | 0.155 | 0.100 | 0.120 | 1.168 | 0.960 | 1.421 |
| 1049 | GCST90319878 | IVW | 2 | -0.137 | 0.120 | 0.252 | 0.872 | 0.689 | 1.102 |
| 1050 | GCST90319878 | IVW | 2 | 0.096 | 0.215 | 0.656 | 1.100 | 0.723 | 1.676 |
| 462 | GCST90319878 | IVW | 2 | 0.303 | 0.141 | 0.032 | 1.354 | 1.026 | 1.786 |
| 1061 | GCST90319878 | IVW | 2 | 0.167 | 0.446 | 0.709 | 1.181 | 0.492 | 2.834 |
| 1063 | GCST90319878 | IVW | 2 | 0.190 | 0.170 | 0.265 | 1.209 | 0.866 | 1.688 |
| 1064 | GCST90319878 | IVW | 3 | 0.032 | 0.126 | 0.798 | 1.033 | 0.807 | 1.321 |
| 1065 | GCST90319878 | IVW | 2 | -0.161 | 0.140 | 0.250 | 0.851 | 0.647 | 1.120 |
| 1070 | GCST90319878 | IVW | 2 | -0.144 | 0.397 | 0.718 | 0.866 | 0.398 | 1.887 |
| 1075 | GCST90319878 | IVW | 2 | -0.003 | 0.137 | 0.982 | 0.997 | 0.762 | 1.305 |
| 1076 | GCST90319878 | IVW | 3 | -0.123 | 0.138 | 0.371 | 0.884 | 0.675 | 1.158 |
| 1078 | GCST90319878 | IVW | 4 | -0.118 | 0.209 | 0.573 | 0.889 | 0.591 | 1.338 |
| 1081 | GCST90319878 | IVW | 4 | 0.295 | 0.104 | 0.005 | 1.344 | 1.095 | 1.648 |
| 947 | GCST90319878 | IVW | 2 | 0.295 | 0.130 | 0.023 | 1.344 | 1.042 | 1.733 |
| 1082 | GCST90319878 | IVW | 2 | 0.089 | 0.148 | 0.547 | 1.093 | 0.817 | 1.463 |
| 1083 | GCST90319878 | IVW | 2 | -0.179 | 0.172 | 0.297 | 0.836 | 0.597 | 1.171 |
| 1088 | GCST90319878 | IVW | 2 | -0.044 | 0.060 | 0.467 | 0.957 | 0.851 | 1.077 |
| 1092 | GCST90319878 | IVW | 2 | -0.191 | 0.141 | 0.175 | 0.826 | 0.627 | 1.089 |
| 1094 | GCST90319878 | IVW | 3 | 0.076 | 0.118 | 0.520 | 1.079 | 0.856 | 1.360 |
| 1095 | GCST90319878 | IVW | 2 | -0.079 | 0.132 | 0.549 | 0.924 | 0.713 | 1.197 |
| 1096 | GCST90319878 | IVW | 2 | -0.101 | 0.405 | 0.803 | 0.904 | 0.409 | 1.997 |
| 1110 | GCST90319878 | IVW | 2 | 0.375 | 0.239 | 0.116 | 1.455 | 0.911 | 2.321 |
| 1111 | GCST90319878 | IVW | 2 | -0.236 | 0.149 | 0.113 | 0.790 | 0.591 | 1.057 |
| 1117 | GCST90319878 | IVW | 2 | -0.079 | 0.137 | 0.565 | 0.924 | 0.707 | 1.208 |
| 1121 | GCST90319878 | IVW | 3 | 0.135 | 0.111 | 0.223 | 1.144 | 0.921 | 1.422 |
| 1123 | GCST90319878 | IVW | 3 | 0.017 | 0.150 | 0.908 | 1.017 | 0.758 | 1.365 |
| 1130 | GCST90319878 | IVW | 2 | -0.137 | 0.244 | 0.575 | 0.872 | 0.540 | 1.407 |
| 1131 | GCST90319878 | IVW | 2 | 0.258 | 0.155 | 0.096 | 1.294 | 0.955 | 1.753 |
| 1133 | GCST90319878 | IVW | 2 | -0.071 | 0.396 | 0.857 | 0.931 | 0.429 | 2.023 |
| 1138 | GCST90319878 | IVW | 3 | 0.010 | 0.155 | 0.948 | 1.010 | 0.745 | 1.369 |
| 1139 | GCST90319878 | IVW | 2 | 0.034 | 0.151 | 0.820 | 1.035 | 0.770 | 1.392 |
| 1141 | GCST90319878 | IVW | 3 | 0.094 | 0.196 | 0.630 | 1.099 | 0.748 | 1.614 |
| 1142 | GCST90319878 | IVW | 5 | 0.148 | 0.097 | 0.125 | 1.160 | 0.960 | 1.402 |
| 1152 | GCST90319878 | IVW | 2 | 0.160 | 0.445 | 0.719 | 1.174 | 0.491 | 2.807 |
| 1154 | GCST90319878 | IVW | 2 | -0.179 | 0.257 | 0.486 | 0.836 | 0.505 | 1.384 |
| 1155 | GCST90319878 | IVW | 2 | 0.124 | 0.147 | 0.401 | 1.132 | 0.848 | 1.511 |
| 1161 | GCST90319878 | IVW | 3 | -0.106 | 0.252 | 0.672 | 0.899 | 0.549 | 1.472 |
| 1162 | GCST90319878 | IVW | 2 | 0.284 | 0.159 | 0.074 | 1.328 | 0.972 | 1.814 |
| 1166 | GCST90319878 | IVW | 3 | 0.008 | 0.098 | 0.937 | 1.008 | 0.831 | 1.222 |
| 1169 | GCST90319878 | IVW | 4 | -0.125 | 0.210 | 0.551 | 0.882 | 0.585 | 1.331 |
| 1171 | GCST90319878 | IVW | 4 | 0.184 | 0.200 | 0.357 | 1.202 | 0.813 | 1.778 |
| 1267 | GCST90319878 | IVW | 3 | 0.294 | 0.112 | 0.009 | 1.341 | 1.077 | 1.671 |
| 1173 | GCST90319878 | IVW | 2 | -0.089 | 0.159 | 0.576 | 0.915 | 0.671 | 1.249 |
| 1175 | GCST90319878 | IVW | 2 | 0.037 | 0.318 | 0.907 | 1.038 | 0.556 | 1.936 |
| 1181 | GCST90319878 | IVW | 3 | -0.111 | 0.170 | 0.511 | 0.895 | 0.642 | 1.247 |
| 1187 | GCST90319878 | IVW | 2 | 0.054 | 0.193 | 0.779 | 1.056 | 0.723 | 1.541 |
| 1192 | GCST90319878 | IVW | 4 | 0.040 | 0.101 | 0.693 | 1.041 | 0.854 | 1.268 |
| 1193 | GCST90319878 | IVW | 5 | 0.061 | 0.117 | 0.601 | 1.063 | 0.845 | 1.338 |
| 1172 | GCST90319878 | IVW | 4 | 0.291 | 0.102 | 0.004 | 1.338 | 1.096 | 1.633 |
| 1202 | GCST90319878 | IVW | 2 | 0.151 | 0.205 | 0.463 | 1.163 | 0.777 | 1.739 |
| 1205 | GCST90319878 | IVW | 3 | -0.007 | 0.092 | 0.939 | 0.993 | 0.829 | 1.190 |
| 1207 | GCST90319878 | IVW | 2 | -0.261 | 0.463 | 0.574 | 0.771 | 0.311 | 1.910 |
| 1211 | GCST90319878 | IVW | 2 | -0.118 | 0.105 | 0.257 | 0.888 | 0.724 | 1.090 |
| 1215 | GCST90319878 | IVW | 4 | 0.066 | 0.099 | 0.504 | 1.068 | 0.880 | 1.296 |
| 1218 | GCST90319878 | IVW | 2 | 0.255 | 0.158 | 0.106 | 1.291 | 0.947 | 1.758 |
| 1219 | GCST90319878 | IVW | 2 | 0.208 | 0.132 | 0.116 | 1.231 | 0.950 | 1.595 |
| 1231 | GCST90319878 | IVW | 4 | -0.071 | 0.102 | 0.485 | 0.931 | 0.762 | 1.137 |
| 1234 | GCST90319878 | IVW | 2 | -0.047 | 0.279 | 0.866 | 0.954 | 0.552 | 1.649 |
| 1245 | GCST90319878 | IVW | 2 | -0.087 | 0.247 | 0.724 | 0.917 | 0.565 | 1.487 |
| 1253 | GCST90319878 | IVW | 2 | 0.159 | 0.414 | 0.700 | 1.172 | 0.521 | 2.638 |
| 1259 | GCST90319878 | IVW | 2 | 0.252 | 0.157 | 0.110 | 1.286 | 0.945 | 1.751 |
| 1262 | GCST90319878 | IVW | 2 | -0.126 | 0.149 | 0.397 | 0.881 | 0.658 | 1.181 |
| 1266 | GCST90319878 | IVW | 5 | 0.167 | 0.112 | 0.134 | 1.182 | 0.950 | 1.472 |
| 1196 | GCST90319878 | IVW | 4 | 0.246 | 0.116 | 0.034 | 1.278 | 1.018 | 1.605 |
| 1273 | GCST90319878 | IVW | 2 | 0.000 | 0.167 | 0.998 | 1.000 | 0.720 | 1.387 |
| 1276 | GCST90319878 | IVW | 3 | 0.189 | 0.224 | 0.399 | 1.208 | 0.779 | 1.873 |
| 1279 | GCST90319878 | IVW | 3 | -0.019 | 0.077 | 0.801 | 0.981 | 0.844 | 1.140 |
| 1281 | GCST90319878 | IVW | 4 | -0.065 | 0.214 | 0.761 | 0.937 | 0.616 | 1.425 |
| 1289 | GCST90319878 | IVW | 3 | -0.140 | 0.168 | 0.405 | 0.869 | 0.625 | 1.209 |
| 1294 | GCST90319878 | IVW | 2 | 0.080 | 0.145 | 0.581 | 1.083 | 0.816 | 1.438 |
| 1305 | GCST90319878 | IVW | 3 | -0.232 | 0.125 | 0.065 | 0.793 | 0.620 | 1.014 |
| 1312 | GCST90319878 | IVW | 3 | -0.138 | 0.184 | 0.454 | 0.871 | 0.607 | 1.250 |
| 1326 | GCST90319878 | IVW | 12 | -0.130 | 0.081 | 0.109 | 0.878 | 0.748 | 1.030 |

BIDPs=brain imaging-derived phenotypes;IVW=inverse-variance weighted;nSNPs= number of single nucleotide polymorphisms;OR=odds ratio;SE=standard error;CI = confidence interval;Pval=P value.

| **Table S3: BIDPs showed significant effect in both discovery cohort and replication cohort** | | | | | | | | | | | | |
| --- | --- | --- | --- | --- | --- | --- | --- | --- | --- | --- | --- | --- |
| BIDPs id | IDP description | IDP short name | Outcome | Trait.s. | Methods | nSNPs | BETA | SE | Pval | OR | OR_lci95 | OR_uci95 |
| 138 | Volume of grey matter in Right I-IV Cerebellum | IDP_T1_FAST_ROIs_R_cerebellum_I-IV | GCST90319877 | cirrhosis of liver | IVW | 4 | -0.236 | 0.107 | 0.027 | 0.790 | 0.641 | 0.974 |
| 237 | Volume of Central-nucleus in the right hemisphere generated by subcortical volumetric sub-segmentation of the Amygdala Nuclei | AmygNuclei_rh_volume_Central-nucleus |  | cirrhosis of liver | IVW | 3 | 0.202 | 0.081 | 0.012 | 1.224 | 1.045 | 1.434 |
| 255 | Volume of CA3-body in the left hemisphere generated by subcortical volumetric sub-segmentation of the Hippocampal Subfields | HippSubfield_lh_volume_CA3-body |  | cirrhosis of liver | IVW | 6 | -0.168 | 0.081 | 0.037 | 0.845 | 0.722 | 0.990 |
| 438 | Volume of caudalanteriorcingulate in the left hemisphere generated by parcellation of the white surface using DKT parcellation | aparc-DKTatlas_lh_volume_caudalanteriorcingulate |  | cirrhosis of liver | IVW | 2 | -0.389 | 0.155 | 0.012 | 0.678 | 0.500 | 0.917 |
| 462 | Volume of rostralmiddlefrontal in the left hemisphere generated by parcellation of the white surface using DKT parcellation | aparc-DKTatlas_lh_volume_rostralmiddlefrontal |  | cirrhosis of liver | IVW | 2 | 0.330 | 0.155 | 0.033 | 1.391 | 1.027 | 1.883 |
| 621 | Volume of S-circular-insula-inf in the right hemisphere generated by parcellation of the white surface using Destrieux (a2009s) parcellation | aparc-a2009s_rh_volume_S-circular-insula-inf |  | cirrhosis of liver | IVW | 2 | 0.371 | 0.151 | 0.014 | 1.450 | 1.078 | 1.949 |
| 651 | Area of caudalmiddlefrontal in the left hemisphere generated by parcellation of the white surface using Desikan-Killiany parcellation | aparc-Desikan_lh_area_caudalmiddlefrontal |  | cirrhosis of liver | IVW | 3 | 0.285 | 0.128 | 0.026 | 1.330 | 1.035 | 1.708 |
| 682 | Area of TotalSurface in the right hemisphere generated by parcellation of the white surface using Desikan-Killiany parcellation | aparc-Desikan_rh_area_TotalSurface |  | cirrhosis of liver | IVW | 5 | -0.321 | 0.088 | 0.000 | 0.725 | 0.610 | 0.862 |
| 691 | Area of isthmuscingulate in the right hemisphere generated by parcellation of the white surface using Desikan-Killiany parcellation | aparc-Desikan_rh_area_isthmuscingulate |  | cirrhosis of liver | IVW | 2 | -0.497 | 0.204 | 0.015 | 0.609 | 0.408 | 0.907 |
| 727 | Area of lateralorbitofrontal in the left hemisphere generated by parcellation of the pial surface using Desikan-Killiany parcellation | aparc-pial_lh_area_lateralorbitofrontal |  | cirrhosis of liver | IVW | 3 | -0.256 | 0.119 | 0.032 | 0.774 | 0.613 | 0.978 |
| 730 | Area of middletemporal in the left hemisphere generated by parcellation of the pial surface using Desikan-Killiany parcellation | aparc-pial_lh_area_middletemporal |  | cirrhosis of liver | IVW | 2 | -0.513 | 0.156 | 0.001 | 0.599 | 0.441 | 0.813 |
| 810 | Area of caudalanteriorcingulate in the left hemisphere generated by parcellation of the white surface using DKT parcellation | aparc-DKTatlas_lh_area_caudalanteriorcingulate |  | cirrhosis of liver | IVW | 2 | -0.382 | 0.153 | 0.013 | 0.683 | 0.506 | 0.921 |
| 811 | Area of caudalmiddlefrontal in the left hemisphere generated by parcellation of the white surface using DKT parcellation | aparc-DKTatlas_lh_area_caudalmiddlefrontal |  | cirrhosis of liver | IVW | 3 | 0.285 | 0.128 | 0.026 | 1.330 | 1.035 | 1.709 |
| 848 | Area of isthmuscingulate in the right hemisphere generated by parcellation of the white surface using DKT parcellation | aparc-DKTatlas_rh_area_isthmuscingulate |  | cirrhosis of liver | IVW | 2 | -0.510 | 0.202 | 0.012 | 0.601 | 0.404 | 0.893 |
| 947 | Area of G+S-occipital-inf in the right hemisphere generated by parcellation of the white surface using Destrieux (a2009s) parcellation | aparc-a2009s_rh_area_G+S-occipital-inf |  | cirrhosis of liver | IVW | 2 | 0.291 | 0.138 | 0.035 | 1.338 | 1.021 | 1.755 |
| 1080 | Mean thickness of rostralmiddlefrontal in the right hemisphere generated by parcellation of the white surface using Desikan-Killiany parcellation | aparc-Desikan_rh_thickness_rostralmiddlefrontal |  | cirrhosis of liver | IVW | 3 | 0.451 | 0.122 | 0.000 | 1.570 | 1.237 | 1.993 |
| 1081 | Mean thickness of superiorfrontal in the right hemisphere generated by parcellation of the white surface using Desikan-Killiany parcellation | aparc-Desikan_rh_thickness_superiorfrontal |  | cirrhosis of liver | IVW | 4 | 0.360 | 0.111 | 0.001 | 1.433 | 1.153 | 1.781 |
| 1172 | Mean thickness of superiorfrontal in the right hemisphere generated by parcellation of the white surface using DKT parcellation | aparc-DKTatlas_rh_thickness_superiorfrontal |  | cirrhosis of liver | IVW | 4 | 0.352 | 0.108 | 0.001 | 1.423 | 1.151 | 1.759 |
| 1196 | Mean thickness of G-occipital-middle in the left hemisphere generated by parcellation of the white surface using Destrieux (a2009s) parcellation | aparc-a2009s_lh_thickness_G-occipital-middle |  | cirrhosis of liver | IVW | 3 | 0.370 | 0.141 | 0.009 | 1.448 | 1.099 | 1.908 |
| 1267 | Mean thickness of G-front-sup in the right hemisphere generated by parcellation of the white surface using Destrieux (a2009s) parcellation | aparc-a2009s_rh_thickness_G-front-sup |  | cirrhosis of liver | IVW | 3 | 0.348 | 0.121 | 0.004 | 1.416 | 1.117 | 1.795 |
| 138 | Volume of grey matter in Right I-IV Cerebellum | IDP_T1_FAST_ROIs_R_cerebellum_I-IV | GCST90319878 | cirrhosis of liver | IVW | 5 | -0.223 | 0.092 | 0.016 | 0.800 | 0.668 | 0.959 |
| 237 | Volume of Central-nucleus in the right hemisphere generated by subcortical volumetric sub-segmentation of the Amygdala Nuclei | AmygNuclei_rh_volume_Central-nucleus |  | cirrhosis of liver | IVW | 2 | 0.352 | 0.153 | 0.021 | 1.421 | 1.054 | 1.918 |
| 255 | Volume of CA3-body in the left hemisphere generated by subcortical volumetric sub-segmentation of the Hippocampal Subfields | HippSubfield_lh_volume_CA3-body |  | cirrhosis of liver | IVW | 6 | -0.153 | 0.076 | 0.044 | 0.858 | 0.739 | 0.996 |
| 438 | Volume of caudalanteriorcingulate in the left hemisphere generated by parcellation of the white surface using DKT parcellation | aparc-DKTatlas_lh_volume_caudalanteriorcingulate |  | cirrhosis of liver | IVW | 2 | -0.411 | 0.145 | 0.004 | 0.663 | 0.499 | 0.880 |
| 462 | Volume of rostralmiddlefrontal in the left hemisphere generated by parcellation of the white surface using DKT parcellation | aparc-DKTatlas_lh_volume_rostralmiddlefrontal |  | cirrhosis of liver | IVW | 2 | 0.303 | 0.141 | 0.032 | 1.354 | 1.026 | 1.786 |
| 621 | Volume of S-circular-insula-inf in the right hemisphere generated by parcellation of the white surface using Destrieux (a2010s) parcellation | aparc-a2009s_rh_volume_S-circular-insula-inf |  | cirrhosis of liver | IVW | 2 | 0.378 | 0.148 | 0.011 | 1.460 | 1.091 | 1.953 |
| 651 | Area of caudalmiddlefrontal in the left hemisphere generated by parcellation of the white surface using Desikan-Killiany parcellation | aparc-Desikan_lh_area_caudalmiddlefrontal |  | cirrhosis of liver | IVW | 3 | 0.317 | 0.117 | 0.007 | 1.373 | 1.091 | 1.727 |
| 682 | Area of TotalSurface in the right hemisphere generated by parcellation of the white surface using Desikan-Killiany parcellation | aparc-Desikan_rh_area_TotalSurface |  | cirrhosis of liver | IVW | 4 | -0.288 | 0.096 | 0.003 | 0.750 | 0.621 | 0.904 |
| 691 | Area of isthmuscingulate in the right hemisphere generated by parcellation of the white surface using Desikan-Killiany parcellation | aparc-Desikan_rh_area_isthmuscingulate |  | cirrhosis of liver | IVW | 2 | -0.483 | 0.205 | 0.018 | 0.617 | 0.412 | 0.922 |
| 727 | Area of lateralorbitofrontal in the left hemisphere generated by parcellation of the pial surface using Desikan-Killiany parcellation | aparc-pial_lh_area_lateralorbitofrontal |  | cirrhosis of liver | IVW | 3 | -0.255 | 0.126 | 0.043 | 0.775 | 0.606 | 0.992 |
| 730 | Area of middletemporal in the left hemisphere generated by parcellation of the pial surface using Desikan-Killiany parcellation | aparc-pial_lh_area_middletemporal |  | cirrhosis of liver | IVW | 2 | -0.462 | 0.143 | 0.001 | 0.630 | 0.476 | 0.834 |
| 810 | Area of caudalanteriorcingulate in the left hemisphere generated by parcellation of the white surface using DKT parcellation | aparc-DKTatlas_lh_area_caudalanteriorcingulate |  | cirrhosis of liver | IVW | 2 | -0.433 | 0.141 | 0.002 | 0.649 | 0.492 | 0.856 |
| 811 | Area of caudalmiddlefrontal in the left hemisphere generated by parcellation of the white surface using DKT parcellation | aparc-DKTatlas_lh_area_caudalmiddlefrontal |  | cirrhosis of liver | IVW | 3 | 0.317 | 0.117 | 0.007 | 1.373 | 1.091 | 1.727 |
| 848 | Area of isthmuscingulate in the right hemisphere generated by parcellation of the white surface using DKT parcellation | aparc-DKTatlas_rh_area_isthmuscingulate |  | cirrhosis of liver | IVW | 2 | -0.496 | 0.204 | 0.015 | 0.609 | 0.408 | 0.908 |
| 947 | Area of G+S-occipital-inf in the right hemisphere generated by parcellation of the white surface using Destrieux (a2009s) parcellation | aparc-a2009s_rh_area_G+S-occipital-inf |  | cirrhosis of liver | IVW | 2 | 0.295 | 0.130 | 0.023 | 1.344 | 1.042 | 1.733 |
| 1080 | Mean thickness of rostralmiddlefrontal in the right hemisphere generated by parcellation of the white surface using Desikan-Killiany parcellation | aparc-Desikan_rh_thickness_rostralmiddlefrontal |  | cirrhosis of liver | IVW | 2 | 0.351 | 0.132 | 0.008 | 1.421 | 1.098 | 1.839 |
| 1081 | Mean thickness of superiorfrontal in the right hemisphere generated by parcellation of the white surface using Desikan-Killiany parcellation | aparc-Desikan_rh_thickness_superiorfrontal |  | cirrhosis of liver | IVW | 4 | 0.295 | 0.104 | 0.005 | 1.344 | 1.095 | 1.648 |
| 1172 | Mean thickness of superiorfrontal in the right hemisphere generated by parcellation of the white surface using DKT parcellation | aparc-DKTatlas_rh_thickness_superiorfrontal |  | cirrhosis of liver | IVW | 4 | 0.291 | 0.102 | 0.004 | 1.338 | 1.096 | 1.633 |
| 1196 | Mean thickness of G-occipital-middle in the left hemisphere generated by parcellation of the white surface using Destrieux (a2009s) parcellation | aparc-a2009s_lh_thickness_G-occipital-middle |  | cirrhosis of liver | IVW | 4 | 0.246 | 0.116 | 0.034 | 1.278 | 1.018 | 1.605 |
| 1267 | Mean thickness of G-front-sup in the right hemisphere generated by parcellation of the white surface using Destrieux (a2009s) parcellation | aparc-a2009s_rh_thickness_G-front-sup |  | cirrhosis of liver | IVW | 3 | 0.294 | 0.112 | 0.009 | 1.341 | 1.077 | 1.671 |

BIDPs=brain imaging-derived phenotypes;IVW=inverse-variance weighted; nSNPs=number of single nucleotide polymorphisms;OR=odds ratio;SE=standard error;CI=confidence interval.

| **Tables S4: Sensitivity Analysis results** | | | | | | | | | | |
| --- | --- | --- | --- | --- | --- | --- | --- | --- | --- | --- |
| BIDPs id | Outcome | Trait | Methods | nSNPs | BETA | SE | Pval | OR | OR_lci95 | OR_uci95 |
| 138 | GCST90319877 | cirrhosis of liver | MR Egger | 4 | -0.09372798 | 0.33090468 | 0.803613898 | 0.910530411 | 0.476017266 | 1.74167134 |
| 138 | GCST90319877 | cirrhosis of liver | Inverse variance weighted | 4 | -0.23571162 | 0.106619254 | 0.027051137 | 0.790008464 | 0.641025938 | 0.973616411 |
| 138 | GCST90319877 | cirrhosis of liver | Simple median | 4 | -0.165324938 | 0.117123219 | 0.158083365 | 0.847618236 | 0.673756542 | 1.066344636 |
| 138 | GCST90319877 | cirrhosis of liver | Weighted median | 4 | -0.166081277 | 0.116791246 | 0.155015995 | 0.846977391 | 0.673685349 | 1.064845336 |
| 138 | GCST90319877 | cirrhosis of liver | Inverse variance weighted (multiplicative random effects) | 4 | -0.23571162 | 0.106619254 | 0.027051137 | 0.790008464 | 0.641025938 | 0.973616411 |
| 138 | GCST90319877 | cirrhosis of liver | Simple mode | 4 | -0.12437758 | 0.156022101 | 0.483609192 | 0.883046357 | 0.65039143 | 1.198925498 |
| 138 | GCST90319877 | cirrhosis of liver | Weighted mode | 4 | -0.149529146 | 0.149424302 | 0.390712191 | 0.86111334 | 0.642492087 | 1.154125006 |
| 138 | GCST90319878 | cirrhosis of liver | MR Egger | 5 | -0.094953407 | 0.257306961 | 0.736613735 | 0.909415306 | 0.549209641 | 1.505866134 |
| 138 | GCST90319878 | cirrhosis of liver | Inverse variance weighted | 5 | -0.222946693 | 0.092330898 | 0.015750388 | 0.800157502 | 0.667700704 | 0.958890748 |
| 138 | GCST90319878 | cirrhosis of liver | Simple median | 5 | -0.194661087 | 0.140254739 | 0.165164165 | 0.823113575 | 0.625277095 | 1.083545139 |
| 138 | GCST90319878 | cirrhosis of liver | Weighted median | 5 | -0.165782732 | 0.112205879 | 0.139545132 | 0.847230291 | 0.679970234 | 1.055633216 |
| 138 | GCST90319878 | cirrhosis of liver | Inverse variance weighted (multiplicative random effects) | 5 | -0.222946693 | 0.073154024 | 0.002306506 | 0.800157502 | 0.693274968 | 0.923518168 |
| 138 | GCST90319878 | cirrhosis of liver | Simple mode | 5 | -0.158626581 | 0.138203789 | 0.315040501 | 0.853314943 | 0.65083052 | 1.118795707 |
| 138 | GCST90319878 | cirrhosis of liver | Weighted mode | 5 | -0.151223894 | 0.120449544 | 0.277637772 | 0.859655206 | 0.678884003 | 1.088561623 |
| 237 | GCST90319877 | cirrhosis of liver | MR Egger | 3 | 0.059702229 | 0.14595427 | 0.752812303 | 1.061520409 | 0.797424483 | 1.413081243 |
| 237 | GCST90319877 | cirrhosis of liver | Inverse variance weighted | 3 | 0.202327299 | 0.080646555 | 0.012113686 | 1.224248638 | 1.045254139 | 1.433895043 |
| 237 | GCST90319877 | cirrhosis of liver | Simple median | 3 | 0.346091696 | 0.135843412 | 0.010842781 | 1.413532225 | 1.083112249 | 1.844751875 |
| 237 | GCST90319877 | cirrhosis of liver | Weighted median | 3 | 0.204351922 | 0.085590893 | 0.016961427 | 1.226729791 | 1.037271566 | 1.450792664 |
| 237 | GCST90319877 | cirrhosis of liver | Inverse variance weighted (multiplicative random effects) | 3 | 0.202327299 | 0.067014137 | 0.002534654 | 1.224248638 | 1.073559315 | 1.396089352 |
| 237 | GCST90319877 | cirrhosis of liver | Simple mode | 3 | 0.357737838 | 0.148519293 | 0.137648881 | 1.430090656 | 1.068910626 | 1.913311773 |
| 237 | GCST90319877 | cirrhosis of liver | Weighted mode | 3 | 0.144517622 | 0.093145262 | 0.260945441 | 1.155482057 | 0.962667606 | 1.386915666 |
| 237 | GCST90319878 | cirrhosis of liver | Inverse variance weighted | 2 | 0.351708441 | 0.152791517 | 0.02134168 | 1.421494014 | 1.053625494 | 1.917802146 |
| 237 | GCST90319878 | cirrhosis of liver | Inverse variance weighted (multiplicative random effects) | 2 | 0.351708441 | 0.015510959 | 7.94E-114 | 1.421494014 | 1.378928794 | 1.465373152 |
| 238 | GCST90319877 | cirrhosis of liver | Inverse variance weighted | 2 | 0.171523531 | 0.082919141 | 0.038587041 | 1.187112076 | 1.009042654 | 1.396606055 |
| 238 | GCST90319877 | cirrhosis of liver | Inverse variance weighted (multiplicative random effects) | 2 | 0.171523531 | 0.072684572 | 0.01828311 | 1.187112076 | 1.029488183 | 1.368869603 |
| 255 | GCST90319877 | cirrhosis of liver | MR Egger | 6 | -0.548163008 | 0.426164941 | 0.267752099 | 0.578010636 | 0.250713032 | 1.332584481 |
| 255 | GCST90319877 | cirrhosis of liver | Inverse variance weighted | 6 | -0.168285315 | 0.080500172 | 0.036573237 | 0.845112677 | 0.7217578 | 0.989550007 |
| 255 | GCST90319877 | cirrhosis of liver | Simple median | 6 | -0.213334877 | 0.102674774 | 0.037730268 | 0.80788555 | 0.660619377 | 0.987980498 |
| 255 | GCST90319877 | cirrhosis of liver | Weighted median | 6 | -0.192472513 | 0.100241962 | 0.054847889 | 0.824916993 | 0.677770354 | 1.004009751 |
| 255 | GCST90319877 | cirrhosis of liver | Inverse variance weighted (multiplicative random effects) | 6 | -0.168285315 | 0.054198284 | 0.001902832 | 0.845112677 | 0.759941402 | 0.939829616 |
| 255 | GCST90319877 | cirrhosis of liver | Simple mode | 6 | -0.275227232 | 0.139309667 | 0.105161288 | 0.759399544 | 0.577946382 | 0.997822092 |
| 255 | GCST90319877 | cirrhosis of liver | Weighted mode | 6 | -0.264892384 | 0.135637447 | 0.108261278 | 0.767288518 | 0.588168516 | 1.00095747 |
| 255 | GCST90319878 | cirrhosis of liver | MR Egger | 6 | -0.479982955 | 0.417833319 | 0.31468377 | 0.618793939 | 0.27282185 | 1.403501733 |
| 255 | GCST90319878 | cirrhosis of liver | Inverse variance weighted | 6 | -0.153143165 | 0.075940353 | 0.043734731 | 0.858006877 | 0.739348225 | 0.99570916 |
| 255 | GCST90319878 | cirrhosis of liver | Simple median | 6 | -0.184093058 | 0.093702532 | 0.049454293 | 0.831858389 | 0.692290219 | 0.999564 |
| 255 | GCST90319878 | cirrhosis of liver | Weighted median | 6 | -0.161577652 | 0.096230398 | 0.093138245 | 0.850800462 | 0.704554752 | 1.02740266 |
| 255 | GCST90319878 | cirrhosis of liver | Inverse variance weighted (multiplicative random effects) | 6 | -0.153143165 | 0.048103291 | 0.001454399 | 0.858006877 | 0.780808299 | 0.942838084 |
| 255 | GCST90319878 | cirrhosis of liver | Simple mode | 6 | -0.235117988 | 0.125159009 | 0.11909762 | 0.790477577 | 0.618517591 | 1.010245803 |
| 255 | GCST90319878 | cirrhosis of liver | Weighted mode | 6 | -0.196208599 | 0.1308924 | 0.194151171 | 0.821840782 | 0.635872185 | 1.062198168 |
| 438 | GCST90319877 | cirrhosis of liver | Inverse variance weighted | 2 | -0.389206018 | 0.154584496 | 0.011810516 | 0.677594659 | 0.500478004 | 0.91739201 |
| 438 | GCST90319877 | cirrhosis of liver | Inverse variance weighted (multiplicative random effects) | 2 | -0.389206018 | 0.102681073 | 0.000150383 | 0.677594659 | 0.554071843 | 0.828655214 |
| 438 | GCST90319878 | cirrhosis of liver | Inverse variance weighted | 2 | -0.41105598 | 0.144676949 | 0.004494428 | 0.662949818 | 0.499262735 | 0.880302957 |
| 438 | GCST90319878 | cirrhosis of liver | Inverse variance weighted (multiplicative random effects) | 2 | -0.41105598 | 0.096740207 | 2.15E-05 | 0.662949818 | 0.548445818 | 0.801359856 |
| 462 | GCST90319877 | cirrhosis of liver | Inverse variance weighted | 2 | 0.329753697 | 0.154657371 | 0.032993995 | 1.390625571 | 1.026982868 | 1.883029929 |
| 462 | GCST90319877 | cirrhosis of liver | Inverse variance weighted (multiplicative random effects) | 2 | 0.329753697 | 0.011838887 | 9.79E-171 | 1.390625571 | 1.358728693 | 1.423271245 |
| 462 | GCST90319878 | cirrhosis of liver | Inverse variance weighted | 2 | 0.302765619 | 0.141380446 | 0.032234084 | 1.353597169 | 1.025991994 | 1.78580857 |
| 462 | GCST90319878 | cirrhosis of liver | Inverse variance weighted (multiplicative random effects) | 2 | 0.302765619 | 0.040363968 | 6.34E-14 | 1.353597169 | 1.250636024 | 1.465034799 |
| 621 | GCST90319877 | cirrhosis of liver | Inverse variance weighted | 2 | 0.371265908 | 0.151016441 | 0.01395394 | 1.449568474 | 1.07817919 | 1.9488864 |
| 621 | GCST90319877 | cirrhosis of liver | Inverse variance weighted (multiplicative random effects) | 2 | 0.371265908 | 0.056051896 | 3.51E-11 | 1.449568474 | 1.298752503 | 1.617897755 |
| 621 | GCST90319878 | cirrhosis of liver | Inverse variance weighted | 2 | 0.378187833 | 0.148465985 | 0.010855916 | 1.459637085 | 1.091108883 | 1.952637773 |
| 621 | GCST90319878 | cirrhosis of liver | Inverse variance weighted (multiplicative random effects) | 2 | 0.378187833 | 0.0412906 | 5.23E-20 | 1.459637085 | 1.34616291 | 1.582676512 |
| 651 | GCST90319877 | cirrhosis of liver | MR Egger | 3 | 1.113514211 | 0.867856304 | 0.421470192 | 3.04504053 | 0.555723648 | 16.68504095 |
| 651 | GCST90319877 | cirrhosis of liver | Inverse variance weighted | 3 | 0.284875705 | 0.127788384 | 0.02579548 | 1.329596756 | 1.035009363 | 1.708030474 |
| 651 | GCST90319877 | cirrhosis of liver | Simple median | 3 | 0.265576034 | 0.170684747 | 0.119721233 | 1.304182013 | 0.933358349 | 1.822334075 |
| 651 | GCST90319877 | cirrhosis of liver | Weighted median | 3 | 0.270888035 | 0.16149626 | 0.093471297 | 1.311128262 | 0.955381412 | 1.799341391 |
| 651 | GCST90319877 | cirrhosis of liver | Inverse variance weighted (multiplicative random effects) | 3 | 0.284875705 | 0.093020405 | 0.002194908 | 1.329596756 | 1.107999029 | 1.595513612 |
| 651 | GCST90319877 | cirrhosis of liver | Simple mode | 3 | 0.236999474 | 0.200855025 | 0.359353784 | 1.267440451 | 0.854980708 | 1.878879001 |
| 651 | GCST90319877 | cirrhosis of liver | Weighted mode | 3 | 0.231214808 | 0.197498412 | 0.362324427 | 1.260129897 | 0.855660084 | 1.855792255 |
| 651 | GCST90319878 | cirrhosis of liver | MR Egger | 3 | 1.013941727 | 0.772142905 | 0.414335689 | 2.756444782 | 0.606859266 | 12.52018097 |
| 651 | GCST90319878 | cirrhosis of liver | Inverse variance weighted | 3 | 0.316751849 | 0.117089951 | 0.006826308 | 1.372661903 | 1.091175489 | 1.726762301 |
| 651 | GCST90319878 | cirrhosis of liver | Simple median | 3 | 0.28505161 | 0.156322525 | 0.06823013 | 1.329830659 | 0.97888556 | 1.80659482 |
| 651 | GCST90319878 | cirrhosis of liver | Weighted median | 3 | 0.296469731 | 0.148718569 | 0.046207265 | 1.345101844 | 1.004993763 | 1.800308656 |
| 651 | GCST90319878 | cirrhosis of liver | Inverse variance weighted (multiplicative random effects) | 3 | 0.316751849 | 0.078523052 | 5.49E-05 | 1.372661903 | 1.176856226 | 1.601045784 |
| 651 | GCST90319878 | cirrhosis of liver | Simple mode | 3 | 0.257462629 | 0.182952922 | 0.294637623 | 1.293643465 | 0.90381996 | 1.851600418 |
| 651 | GCST90319878 | cirrhosis of liver | Weighted mode | 3 | 0.247983824 | 0.18751107 | 0.316972792 | 1.281439204 | 0.887330401 | 1.850591877 |
| 677 | GCST90319877 | cirrhosis of liver | Inverse variance weighted | 2 | 0.335302236 | 0.154577958 | 0.030071564 | 1.398362957 | 1.032857712 | 1.893212333 |
| 677 | GCST90319877 | cirrhosis of liver | Inverse variance weighted (multiplicative random effects) | 2 | 0.335302236 | 0.003365338 | 0 | 1.398362957 | 1.38916962 | 1.407617134 |
| 677 | GCST90319878 | cirrhosis of liver | Inverse variance weighted | 2 | 0.309538102 | 0.144068538 | 0.031670285 | 1.362795497 | 1.027536065 | 1.807441732 |
| 677 | GCST90319878 | cirrhosis of liver | Inverse variance weighted (multiplicative random effects) | 2 | 0.309538102 | 0.101932878 | 0.002391948 | 1.362795497 | 1.115998658 | 1.664170072 |
| 682 | GCST90319877 | cirrhosis of liver | MR Egger | 5 | -0.307985739 | 0.372730628 | 0.469220294 | 0.734925799 | 0.353971892 | 1.525872369 |
| 682 | GCST90319877 | cirrhosis of liver | Inverse variance weighted | 5 | -0.321185253 | 0.08822172 | 0.00027194 | 0.725288877 | 0.610119875 | 0.862197702 |
| 682 | GCST90319877 | cirrhosis of liver | Simple median | 5 | -0.301571944 | 0.116848035 | 0.009854593 | 0.739654611 | 0.588255388 | 0.930019435 |
| 682 | GCST90319877 | cirrhosis of liver | Weighted median | 5 | -0.28032119 | 0.109109614 | 0.010194232 | 0.755541031 | 0.61007338 | 0.935694406 |
| 682 | GCST90319877 | cirrhosis of liver | Inverse variance weighted (multiplicative random effects) | 5 | -0.321185253 | 0.052484873 | 9.38E-10 | 0.725288877 | 0.654387495 | 0.80387226 |
| 682 | GCST90319877 | cirrhosis of liver | Simple mode | 5 | -0.264247263 | 0.144721476 | 0.141901025 | 0.767783672 | 0.578161894 | 1.019596367 |
| 682 | GCST90319877 | cirrhosis of liver | Weighted mode | 5 | -0.267103557 | 0.131393921 | 0.111849952 | 0.765593784 | 0.591770941 | 0.990474188 |
| 682 | GCST90319878 | cirrhosis of liver | MR Egger | 4 | -0.514546886 | 0.402875122 | 0.329762049 | 0.597771392 | 0.271394411 | 1.316647001 |
| 682 | GCST90319878 | cirrhosis of liver | Inverse variance weighted | 4 | -0.288280897 | 0.095583339 | 0.002561196 | 0.749551016 | 0.621496971 | 0.903989483 |
| 682 | GCST90319878 | cirrhosis of liver | Simple median | 4 | -0.308546169 | 0.120839656 | 0.010669164 | 0.73451404 | 0.579614591 | 0.930809686 |
| 682 | GCST90319878 | cirrhosis of liver | Weighted median | 4 | -0.232339559 | 0.120886115 | 0.054609457 | 0.792676917 | 0.625454719 | 1.00460781 |
| 682 | GCST90319878 | cirrhosis of liver | Inverse variance weighted (multiplicative random effects) | 4 | -0.288280897 | 0.068720644 | 2.73E-05 | 0.749551016 | 0.655096085 | 0.857624917 |
| 682 | GCST90319878 | cirrhosis of liver | Simple mode | 4 | -0.246722325 | 0.148104012 | 0.194328909 | 0.781357627 | 0.584495505 | 1.044524269 |
| 682 | GCST90319878 | cirrhosis of liver | Weighted mode | 4 | -0.224938297 | 0.123750529 | 0.166707083 | 0.798565491 | 0.626573412 | 1.017768759 |
| 691 | GCST90319877 | cirrhosis of liver | Inverse variance weighted | 2 | -0.496517678 | 0.203756777 | 0.014817236 | 0.608646477 | 0.40824779 | 0.907415896 |
| 691 | GCST90319877 | cirrhosis of liver | Inverse variance weighted (multiplicative random effects) | 2 | -0.496517678 | 0.203756777 | 0.014817236 | 0.608646477 | 0.40824779 | 0.907415896 |
| 691 | GCST90319878 | cirrhosis of liver | Inverse variance weighted | 2 | -0.48338305 | 0.205212395 | 0.018496523 | 0.616693554 | 0.412466889 | 0.922039924 |
| 691 | GCST90319878 | cirrhosis of liver | Inverse variance weighted (multiplicative random effects) | 2 | -0.48338305 | 0.205212395 | 0.018496523 | 0.616693554 | 0.412466889 | 0.922039924 |
| 711 | GCST90319877 | cirrhosis of liver | Inverse variance weighted | 2 | -0.445378871 | 0.156862978 | 0.00452146 | 0.640581532 | 0.4710315 | 0.871161906 |
| 711 | GCST90319877 | cirrhosis of liver | Inverse variance weighted (multiplicative random effects) | 2 | -0.445378871 | 0.033612721 | 4.49E-40 | 0.640581532 | 0.599739548 | 0.684204837 |
| 711 | GCST90319878 | cirrhosis of liver | Inverse variance weighted | 2 | -0.363553692 | 0.145915479 | 0.012719202 | 0.695201399 | 0.522281778 | 0.925372099 |
| 711 | GCST90319878 | cirrhosis of liver | Inverse variance weighted (multiplicative random effects) | 2 | -0.363553692 | 0.091593785 | 7.21E-05 | 0.695201399 | 0.580957595 | 0.831910951 |
| 727 | GCST90319877 | cirrhosis of liver | MR Egger | 3 | 0.395761447 | 1.355645924 | 0.819173224 | 1.485514901 | 0.104214442 | 21.17513166 |
| 727 | GCST90319877 | cirrhosis of liver | Inverse variance weighted | 3 | -0.255987773 | 0.119435519 | 0.032087926 | 0.774151435 | 0.612576534 | 0.978343783 |
| 727 | GCST90319877 | cirrhosis of liver | Simple median | 3 | -0.21527391 | 0.164788339 | 0.191428067 | 0.806320551 | 0.58376367 | 1.113726093 |
| 727 | GCST90319877 | cirrhosis of liver | Weighted median | 3 | -0.231694898 | 0.155110627 | 0.135243674 | 0.79318809 | 0.585252602 | 1.075001365 |
| 727 | GCST90319877 | cirrhosis of liver | Inverse variance weighted (multiplicative random effects) | 3 | -0.255987773 | 0.111654339 | 0.021866382 | 0.774151435 | 0.621990614 | 0.963536154 |
| 727 | GCST90319877 | cirrhosis of liver | Simple mode | 3 | -0.175261447 | 0.198260299 | 0.469952247 | 0.839237576 | 0.569013207 | 1.237791494 |
| 727 | GCST90319877 | cirrhosis of liver | Weighted mode | 3 | -0.182225351 | 0.199776728 | 0.457978371 | 0.833413509 | 0.563387428 | 1.232860448 |
| 727 | GCST90319878 | cirrhosis of liver | MR Egger | 3 | 0.433713793 | 1.621050346 | 0.833569817 | 1.542977194 | 0.064341858 | 37.00201874 |
| 727 | GCST90319878 | cirrhosis of liver | Inverse variance weighted | 3 | -0.254891905 | 0.125877348 | 0.042875298 | 0.775000267 | 0.605554018 | 0.991861 |
| 727 | GCST90319878 | cirrhosis of liver | Simple median | 3 | -0.185165671 | 0.162853465 | 0.255534998 | 0.830966606 | 0.603892871 | 1.14342383 |
| 727 | GCST90319878 | cirrhosis of liver | Weighted median | 3 | -0.223330552 | 0.147476344 | 0.129937524 | 0.799850414 | 0.599065591 | 1.067930948 |
| 727 | GCST90319878 | cirrhosis of liver | Inverse variance weighted (multiplicative random effects) | 3 | -0.254891905 | 0.125877348 | 0.042875298 | 0.775000267 | 0.605554018 | 0.991861 |
| 727 | GCST90319878 | cirrhosis of liver | Simple mode | 3 | -0.138830282 | 0.192589301 | 0.545867436 | 0.870375734 | 0.596721179 | 1.269527454 |
| 727 | GCST90319878 | cirrhosis of liver | Weighted mode | 3 | -0.156931768 | 0.207359162 | 0.528167653 | 0.854762379 | 0.569295455 | 1.283373542 |
| 730 | GCST90319877 | cirrhosis of liver | Inverse variance weighted | 2 | -0.512504663 | 0.156122 | 0.001028111 | 0.598993422 | 0.441091129 | 0.813421752 |
| 730 | GCST90319877 | cirrhosis of liver | Inverse variance weighted (multiplicative random effects) | 2 | -0.512504663 | 0.030355681 | 5.97E-64 | 0.598993422 | 0.564394504 | 0.635713347 |
| 730 | GCST90319878 | cirrhosis of liver | Inverse variance weighted | 2 | -0.462077099 | 0.142916104 | 0.001224096 | 0.629973768 | 0.476068912 | 0.833633406 |
| 730 | GCST90319878 | cirrhosis of liver | Inverse variance weighted (multiplicative random effects) | 2 | -0.462077099 | 0.033646064 | 6.40E-43 | 0.629973768 | 0.589769566 | 0.672918663 |
| 810 | GCST90319877 | cirrhosis of liver | Inverse variance weighted | 2 | -0.381815772 | 0.152954433 | 0.012550654 | 0.682620799 | 0.505803786 | 0.921248849 |
| 810 | GCST90319877 | cirrhosis of liver | Inverse variance weighted (multiplicative random effects) | 2 | -0.381815772 | 0.116837327 | 0.001083386 | 0.682620799 | 0.542907169 | 0.858288824 |
| 810 | GCST90319878 | cirrhosis of liver | Inverse variance weighted | 2 | -0.432708648 | 0.141202511 | 0.002180675 | 0.648749479 | 0.491907017 | 0.855600493 |
| 810 | GCST90319878 | cirrhosis of liver | Inverse variance weighted (multiplicative random effects) | 2 | -0.432708648 | 0.0790662 | 4.43E-08 | 0.648749479 | 0.555615706 | 0.75749458 |
| 811 | GCST90319877 | cirrhosis of liver | MR Egger | 3 | 1.109510691 | 0.86560998 | 0.421782037 | 3.032874019 | 0.555945579 | 16.54536914 |
| 811 | GCST90319877 | cirrhosis of liver | Inverse variance weighted | 3 | 0.285051648 | 0.127852744 | 0.025778599 | 1.32983071 | 1.035060905 | 1.708546529 |
| 811 | GCST90319877 | cirrhosis of liver | Simple median | 3 | 0.265995177 | 0.170261217 | 0.118222757 | 1.304728765 | 0.934525085 | 1.821585293 |
| 811 | GCST90319877 | cirrhosis of liver | Weighted median | 3 | 0.27138299 | 0.160574887 | 0.091014335 | 1.311777372 | 0.957582129 | 1.796984115 |
| 811 | GCST90319877 | cirrhosis of liver | Inverse variance weighted (multiplicative random effects) | 3 | 0.285051648 | 0.093017523 | 0.002180385 | 1.32983071 | 1.108200249 | 1.595785345 |
| 811 | GCST90319877 | cirrhosis of liver | Simple mode | 3 | 0.238935068 | 0.193661897 | 0.342601408 | 1.269896077 | 0.868800069 | 1.85616473 |
| 811 | GCST90319877 | cirrhosis of liver | Weighted mode | 3 | 0.233152574 | 0.175687909 | 0.315713559 | 1.2625741 | 0.894763602 | 1.781580471 |
| 811 | GCST90319878 | cirrhosis of liver | MR Egger | 3 | 1.010778765 | 0.770238131 | 0.414536521 | 2.747740024 | 0.607205514 | 12.43413486 |
| 811 | GCST90319878 | cirrhosis of liver | Inverse variance weighted | 3 | 0.316921585 | 0.117140719 | 0.006820626 | 1.372894913 | 1.091252126 | 1.727227279 |
| 811 | GCST90319878 | cirrhosis of liver | Simple median | 3 | 0.28550149 | 0.152452518 | 0.061106508 | 1.330429057 | 0.98678268 | 1.793750045 |
| 811 | GCST90319878 | cirrhosis of liver | Weighted median | 3 | 0.296975383 | 0.141136402 | 0.035363373 | 1.34578217 | 1.020556468 | 1.774649131 |
| 811 | GCST90319878 | cirrhosis of liver | Inverse variance weighted (multiplicative random effects) | 3 | 0.316921585 | 0.078491721 | 5.40E-05 | 1.372894913 | 1.177128282 | 1.60121923 |
| 811 | GCST90319878 | cirrhosis of liver | Simple mode | 3 | 0.257497926 | 0.172771349 | 0.27459634 | 1.293689128 | 0.922070201 | 1.81508041 |
| 811 | GCST90319878 | cirrhosis of liver | Weighted mode | 3 | 0.248025062 | 0.184794235 | 0.311610271 | 1.281492048 | 0.892104822 | 1.840839583 |
| 848 | GCST90319877 | cirrhosis of liver | Inverse variance weighted | 2 | -0.509785643 | 0.202450229 | 0.01179969 | 0.600624313 | 0.403899945 | 0.893165671 |
| 848 | GCST90319877 | cirrhosis of liver | Inverse variance weighted (multiplicative random effects) | 2 | -0.509785643 | 0.202450229 | 0.01179969 | 0.600624313 | 0.403899945 | 0.893165671 |
| 848 | GCST90319878 | cirrhosis of liver | Inverse variance weighted | 2 | -0.496032565 | 0.20396573 | 0.015018165 | 0.60894181 | 0.40827864 | 0.908228087 |
| 848 | GCST90319878 | cirrhosis of liver | Inverse variance weighted (multiplicative random effects) | 2 | -0.496032565 | 0.20396573 | 0.015018165 | 0.60894181 | 0.40827864 | 0.908228087 |
| 947 | GCST90319877 | cirrhosis of liver | Inverse variance weighted | 2 | 0.291294018 | 0.138217231 | 0.035073507 | 1.338157968 | 1.020597506 | 1.754527851 |
| 947 | GCST90319877 | cirrhosis of liver | Inverse variance weighted (multiplicative random effects) | 2 | 0.291294018 | 0.062544714 | 3.20E-06 | 1.338157968 | 1.183772506 | 1.512678103 |
| 947 | GCST90319878 | cirrhosis of liver | Inverse variance weighted | 2 | 0.295439087 | 0.129925611 | 0.022971426 | 1.343716238 | 1.041628016 | 1.733414713 |
| 947 | GCST90319878 | cirrhosis of liver | Inverse variance weighted (multiplicative random effects) | 2 | 0.295439087 | 0.120110951 | 0.013904616 | 1.343716238 | 1.061859508 | 1.700388153 |
| 1080 | GCST90319877 | cirrhosis of liver | MR Egger | 3 | -0.400776942 | 1.84146887 | 0.863573677 | 0.669799449 | 0.018132381 | 24.741996 |
| 1080 | GCST90319877 | cirrhosis of liver | Inverse variance weighted | 3 | 0.451226107 | 0.121699487 | 0.000209144 | 1.570236284 | 1.237007446 | 1.993231322 |
| 1080 | GCST90319877 | cirrhosis of liver | Simple median | 3 | 0.487884801 | 0.164274832 | 0.002978673 | 1.628867195 | 1.180462287 | 2.247601104 |
| 1080 | GCST90319877 | cirrhosis of liver | Weighted median | 3 | 0.478509186 | 0.152998074 | 0.001762696 | 1.613666931 | 1.195581839 | 2.177952925 |
| 1080 | GCST90319877 | cirrhosis of liver | Inverse variance weighted (multiplicative random effects) | 3 | 0.451226107 | 0.041475594 | 1.45E-27 | 1.570236284 | 1.447638984 | 1.703216074 |
| 1080 | GCST90319877 | cirrhosis of liver | Simple mode | 3 | 0.49532787 | 0.168210709 | 0.098568184 | 1.641036198 | 1.180142131 | 2.281928364 |
| 1080 | GCST90319877 | cirrhosis of liver | Weighted mode | 3 | 0.496204862 | 0.191507438 | 0.122234759 | 1.642476005 | 1.128455951 | 2.390636005 |
| 1080 | GCST90319878 | cirrhosis of liver | Inverse variance weighted | 2 | 0.351258161 | 0.131567688 | 0.007589859 | 1.420854087 | 1.09788488 | 1.838832443 |
| 1080 | GCST90319878 | cirrhosis of liver | Inverse variance weighted (multiplicative random effects) | 2 | 0.351258161 | 0.103444831 | 0.000684762 | 1.420854087 | 1.160100097 | 1.740217367 |
| 1081 | GCST90319877 | cirrhosis of liver | MR Egger | 4 | 0.962230775 | 0.732384518 | 0.319372425 | 2.617529085 | 0.622978744 | 10.99790094 |
| 1081 | GCST90319877 | cirrhosis of liver | Inverse variance weighted | 4 | 0.360008455 | 0.110825171 | 0.001160414 | 1.433341534 | 1.153488836 | 1.781090452 |
| 1081 | GCST90319877 | cirrhosis of liver | Simple median | 4 | 0.374858933 | 0.134771519 | 0.005411828 | 1.454786178 | 1.117067286 | 1.894606396 |
| 1081 | GCST90319877 | cirrhosis of liver | Weighted median | 4 | 0.388549337 | 0.131644532 | 0.003162307 | 1.474839745 | 1.139427625 | 1.908986781 |
| 1081 | GCST90319877 | cirrhosis of liver | Inverse variance weighted (multiplicative random effects) | 4 | 0.360008455 | 0.081678284 | 1.05E-05 | 1.433341534 | 1.22130385 | 1.682192316 |
| 1081 | GCST90319877 | cirrhosis of liver | Simple mode | 4 | 0.438146153 | 0.176307735 | 0.088866401 | 1.549831403 | 1.09700418 | 2.189579055 |
| 1081 | GCST90319877 | cirrhosis of liver | Weighted mode | 4 | 0.430308617 | 0.168399634 | 0.083556959 | 1.537732021 | 1.105442088 | 2.139071594 |
| 1081 | GCST90319878 | cirrhosis of liver | MR Egger | 4 | 0.202675014 | 0.656625938 | 0.78676328 | 1.224674401 | 0.338134402 | 4.43559538 |
| 1081 | GCST90319878 | cirrhosis of liver | Inverse variance weighted | 4 | 0.295482871 | 0.104259122 | 0.004595206 | 1.343775072 | 1.095416868 | 1.648442247 |
| 1081 | GCST90319878 | cirrhosis of liver | Simple median | 4 | 0.273627267 | 0.126621489 | 0.03069642 | 1.314724669 | 1.025775743 | 1.6850671 |
| 1081 | GCST90319878 | cirrhosis of liver | Weighted median | 4 | 0.303247121 | 0.129583493 | 0.019275076 | 1.354249087 | 1.050497094 | 1.745831187 |
| 1081 | GCST90319878 | cirrhosis of liver | Inverse variance weighted (multiplicative random effects) | 4 | 0.295482871 | 0.063189765 | 2.92E-06 | 1.343775072 | 1.187239581 | 1.520949498 |
| 1081 | GCST90319878 | cirrhosis of liver | Simple mode | 4 | 0.302203812 | 0.165594259 | 0.165498503 | 1.352836923 | 0.977887218 | 1.871552985 |
| 1081 | GCST90319878 | cirrhosis of liver | Weighted mode | 4 | 0.332752465 | 0.167312352 | 0.14084655 | 1.394801994 | 1.004831892 | 1.93611749 |
| 1172 | GCST90319877 | cirrhosis of liver | MR Egger | 4 | 1.154711228 | 0.792198315 | 0.282291484 | 3.17310698 | 0.671663727 | 14.99054885 |
| 1172 | GCST90319877 | cirrhosis of liver | Inverse variance weighted | 4 | 0.352420528 | 0.108257304 | 0.001132339 | 1.422506602 | 1.150545539 | 1.758752665 |
| 1172 | GCST90319877 | cirrhosis of liver | Simple median | 4 | 0.360373251 | 0.130295439 | 0.005678051 | 1.433864506 | 1.110704157 | 1.851048643 |
| 1172 | GCST90319877 | cirrhosis of liver | Weighted median | 4 | 0.372592536 | 0.135375793 | 0.00591813 | 1.451492788 | 1.113219182 | 1.8925575 |
| 1172 | GCST90319877 | cirrhosis of liver | Inverse variance weighted (multiplicative random effects) | 4 | 0.352420528 | 0.078669537 | 7.47E-06 | 1.422506602 | 1.219240637 | 1.659660097 |
| 1172 | GCST90319877 | cirrhosis of liver | Simple mode | 4 | 0.408417972 | 0.191433967 | 0.122608227 | 1.504435842 | 1.033764917 | 2.189402218 |
| 1172 | GCST90319877 | cirrhosis of liver | Weighted mode | 4 | 0.400244232 | 0.161201138 | 0.08904219 | 1.492189093 | 1.087944354 | 2.04663803 |
| 1172 | GCST90319878 | cirrhosis of liver | MR Egger | 4 | 0.3406692 | 0.719086289 | 0.6823555 | 1.405888097 | 0.343441006 | 5.755053431 |
| 1172 | GCST90319878 | cirrhosis of liver | Inverse variance weighted | 4 | 0.291013832 | 0.101769048 | 0.004242406 | 1.337783088 | 1.095867731 | 1.633101825 |
| 1172 | GCST90319878 | cirrhosis of liver | Simple median | 4 | 0.276223654 | 0.120271148 | 0.021637357 | 1.318142639 | 1.041323191 | 1.668550199 |
| 1172 | GCST90319878 | cirrhosis of liver | Weighted median | 4 | 0.311992579 | 0.123166043 | 0.011305693 | 1.366144554 | 1.073138072 | 1.73915267 |
| 1172 | GCST90319878 | cirrhosis of liver | Inverse variance weighted (multiplicative random effects) | 4 | 0.291013832 | 0.057485693 | 4.14E-07 | 1.337783088 | 1.195233869 | 1.497333397 |
| 1172 | GCST90319878 | cirrhosis of liver | Simple mode | 4 | 0.308495318 | 0.164314454 | 0.157087801 | 1.361375135 | 0.986530525 | 1.878646643 |
| 1172 | GCST90319878 | cirrhosis of liver | Weighted mode | 4 | 0.33724445 | 0.156583959 | 0.120280578 | 1.401081517 | 1.030804834 | 1.904365745 |
| 1196 | GCST90319877 | cirrhosis of liver | MR Egger | 3 | 0.837408008 | 1.454025127 | 0.667348067 | 2.310370747 | 0.133656629 | 39.93676213 |
| 1196 | GCST90319877 | cirrhosis of liver | Inverse variance weighted | 3 | 0.370110172 | 0.140777522 | 0.008562551 | 1.447894124 | 1.098764349 | 1.907959059 |
| 1196 | GCST90319877 | cirrhosis of liver | Simple median | 3 | 0.472433825 | 0.167505402 | 0.00479624 | 1.603893042 | 1.155026442 | 2.227198268 |
| 1196 | GCST90319877 | cirrhosis of liver | Weighted median | 3 | 0.425221599 | 0.148664007 | 0.004232589 | 1.529929413 | 1.143210058 | 2.04746625 |
| 1196 | GCST90319877 | cirrhosis of liver | Inverse variance weighted (multiplicative random effects) | 3 | 0.370110172 | 0.140777522 | 0.008562551 | 1.447894124 | 1.098764349 | 1.907959059 |
| 1196 | GCST90319877 | cirrhosis of liver | Simple mode | 3 | 0.514756039 | 0.207989091 | 0.131752997 | 1.67323025 | 1.113042682 | 2.515356791 |
| 1196 | GCST90319877 | cirrhosis of liver | Weighted mode | 3 | 0.516726052 | 0.233469901 | 0.157337604 | 1.676529783 | 1.060907895 | 2.649383726 |
| 1196 | GCST90319878 | cirrhosis of liver | MR Egger | 4 | 1.195356549 | 0.919745919 | 0.323343115 | 3.30473586 | 0.544795136 | 20.04657968 |
| 1196 | GCST90319878 | cirrhosis of liver | Inverse variance weighted | 4 | 0.245605565 | 0.116061676 | 0.034330844 | 1.27839523 | 1.018289929 | 1.604940124 |
| 1196 | GCST90319878 | cirrhosis of liver | Simple median | 4 | 0.402828684 | 0.181498455 | 0.0264554 | 1.496050572 | 1.048218139 | 2.135211395 |
| 1196 | GCST90319878 | cirrhosis of liver | Weighted median | 4 | 0.290746458 | 0.140578047 | 0.038619254 | 1.337425448 | 1.0153298 | 1.761700314 |
| 1196 | GCST90319878 | cirrhosis of liver | Inverse variance weighted (multiplicative random effects) | 4 | 0.245605565 | 0.116061676 | 0.034330844 | 1.27839523 | 1.018289929 | 1.604940124 |
| 1196 | GCST90319878 | cirrhosis of liver | Simple mode | 4 | 0.44795015 | 0.178412052 | 0.086879339 | 1.565100674 | 1.103252382 | 2.2202899 |
| 1196 | GCST90319878 | cirrhosis of liver | Weighted mode | 4 | 0.392423908 | 0.154467545 | 0.084642933 | 1.480565202 | 1.093810554 | 2.004070367 |
| 1267 | GCST90319877 | cirrhosis of liver | MR Egger | 3 | 3.108432713 | 3.928785201 | 0.573879484 | 22.3859317 | 0.010132712 | 49456.64499 |
| 1267 | GCST90319877 | cirrhosis of liver | Inverse variance weighted | 3 | 0.347906462 | 0.120838649 | 0.003988128 | 1.416099785 | 1.117464981 | 1.794542678 |
| 1267 | GCST90319877 | cirrhosis of liver | Simple median | 3 | 0.397865114 | 0.156849658 | 0.011193422 | 1.488643219 | 1.094655589 | 2.024434586 |
| 1267 | GCST90319877 | cirrhosis of liver | Weighted median | 3 | 0.407399921 | 0.138616327 | 0.00329223 | 1.502905029 | 1.145351893 | 1.972078223 |
| 1267 | GCST90319877 | cirrhosis of liver | Inverse variance weighted (multiplicative random effects) | 3 | 0.347906462 | 0.097088197 | 0.000339142 | 1.416099785 | 1.170713738 | 1.712919679 |
| 1267 | GCST90319877 | cirrhosis of liver | Simple mode | 3 | 0.415291376 | 0.181153996 | 0.148915289 | 1.514812057 | 1.062080313 | 2.160529236 |
| 1267 | GCST90319877 | cirrhosis of liver | Weighted mode | 3 | 0.418670268 | 0.187347282 | 0.154990513 | 1.5199391 | 1.052817175 | 2.19431723 |
| 1267 | GCST90319878 | cirrhosis of liver | MR Egger | 3 | 3.533830596 | 3.651201692 | 0.510398577 | 34.25493343 | 0.026715143 | 43922.67199 |
| 1267 | GCST90319878 | cirrhosis of liver | Inverse variance weighted | 3 | 0.293727459 | 0.112115934 | 0.008796723 | 1.341418262 | 1.076785515 | 1.67108763 |
| 1267 | GCST90319878 | cirrhosis of liver | Simple median | 3 | 0.266860747 | 0.144300749 | 0.064409133 | 1.305858589 | 0.984158104 | 1.732716165 |
| 1267 | GCST90319878 | cirrhosis of liver | Weighted median | 3 | 0.307186896 | 0.129051228 | 0.017296272 | 1.359595047 | 1.0557448 | 1.750895379 |
| 1267 | GCST90319878 | cirrhosis of liver | Inverse variance weighted (multiplicative random effects) | 3 | 0.293727459 | 0.081209685 | 0.00029815 | 1.341418262 | 1.144029267 | 1.572864441 |
| 1267 | GCST90319878 | cirrhosis of liver | Simple mode | 3 | 0.300453633 | 0.158587518 | 0.198640389 | 1.350471287 | 0.989675763 | 1.842798183 |
| 1267 | GCST90319878 | cirrhosis of liver | Weighted mode | 3 | 0.331789207 | 0.161094404 | 0.175629736 | 1.393459086 | 1.016173573 | 1.910823383 |

BIDPs=brain imaging-derived phenotypes;MR=Mendelian randomization;IVW=inverse-variance weighted;MR-Egger=Mendelian randomization Egger regression;nSNPs=number of single nucleotide polymorphisms;OR=odds ratio;SE=standard error;CI=confidence interval.

| **Table S5: Heterogeneity test and Pleiotropy test result** | | | | | | | | |
| --- | --- | --- | --- | --- | --- | --- | --- | --- |
| BIDPs id | Outcome | MR-Egger intercept | | | Cochran’s Q | | | MR-PRESSO |
|  |  | Intercept | SE | Pval | Q | DF | Pval | Pval |
| 138 | GCST90319877 | -0.011 | 0.024 | 0.689 | 3.519770791 | 3 | 0.318206954 | 0.194977101 |
| 237 |  | 0.025 | 0.021 | 0.450 | 1.380992158 | 2 | 0.501327309 | 0.143581241 |
| 255 |  | 0.026 | 0.029 | 0.415 | 2.266456841 | 5 | 0.811180639 | 0.753265659 |
| 438 |  | NA | NA | NA | 0.441213636 | 1 | 0.506537266 | 0.753443564 |
| 462 |  | NA | NA | NA | 0.005859769 | 1 | 0.938982228 | 0.095252704 |
| 621 |  | NA | NA | NA | 0.137762862 | 1 | 0.710515237 | 0.249355541 |
| 651 |  | -0.052 | 0.053 | 0.511 | 1.059750648 | 2 | 0.588678359 | 0.095569405 |
| 682 |  | -0.001 | 0.038 | 0.973 | 1.41571961 | 4 | 0.841458322 | 0.093340774 |
| 691 |  | NA | NA | NA | 1.675326824 | 1 | 0.195546567 | 0.964947652 |
| 727 |  | -0.049 | 0.102 | 0.713 | 1.747890413 | 2 | 0.417301955 | 0.661551582 |
| 730 |  | NA | NA | NA | 0.037805217 | 1 | 0.845834788 | 0.966149154 |
| 810 |  | NA | NA | NA | 0.583497624 | 1 | 0.44494462 | 0.545071134 |
| 811 |  | -0.051 | 0.053 | 0.512 | 1.058618393 | 2 | 0.589011721 | 0.36627834 |
| 848 |  | NA | NA | NA | 1.583432245 | 1 | 0.208266998 | 0.557550417 |
| 947 |  | NA | NA | NA | 0.204765531 | 1 | 0.650901435 | 0.519900588 |
| 1080 |  | 0.050 | 0.108 | 0.724 | 0.232293864 | 2 | 0.890344394 | 0.202294495 |
| 1081 |  | -0.041 | 0.049 | 0.493 | 1.629512328 | 3 | 0.652716765 | 0.536522041 |
| 1172 |  | -0.056 | 0.054 | 0.414 | 1.584236528 | 3 | 0.66296934 | 0.681319011 |
| 1196 |  | -0.033 | 0.102 | 0.800 | 2.916638284 | 2 | 0.232626959 | 0.639409596 |
| 1267 |  | -0.175 | 0.249 | 0.610 | 1.291074041 | 2 | 0.524380863 | 0.651398006 |
| 138 | GCST90319878 | -0.010 | 0.018 | 0.631 | 2.510974534 | 4 | 0.642671717 | 0.78148562 |
| 237 |  | NA | NA | NA | 0.010305731 | 1 | 0.919139946 | 0.403354631 |
| 255 |  | 0.022 | 0.028 | 0.471 | 2.006200606 | 5 | 0.8482864 | 0.18345075 |
| 438 |  | NA | NA | NA | 0.44711099 | 1 | 0.503710102 | 0.432379965 |
| 462 |  | NA | NA | NA | 0.081509647 | 1 | 0.775261913 | 0.288817792 |
| 621 |  | NA | NA | NA | 0.077347888 | 1 | 0.780924171 | 0.086222394 |
| 651 |  | -0.044 | 0.048 | 0.529 | 0.899466695 | 2 | 0.6377982 | 0.218221532 |
| 682 |  | 0.022 | 0.038 | 0.622 | 1.550712549 | 3 | 0.670616314 | 0.067576596 |
| 691 |  | NA | NA | NA | 1.868367169 | 1 | 0.171662207 | 0.179856145 |
| 727 |  | -0.052 | 0.121 | 0.743 | 2.468932374 | 2 | 0.290990055 | 0.695455941 |
| 730 |  | NA | NA | NA | 0.055425063 | 1 | 0.813878666 | 0.283686222 |
| 810 |  | NA | NA | NA | 0.313542841 | 1 | 0.57551425 | 0.510513452 |
| 811 |  | -0.044 | 0.048 | 0.529 | 0.897970206 | 2 | 0.638275607 | 0.387148456 |
| 848 |  | NA | NA | NA | 1.769512577 | 1 | 0.183442413 | 0.756936367 |
| 947 |  | NA | NA | NA | 0.854625153 | 1 | 0.355247189 | 0.34167012 |
| 1080 |  | NA | NA | NA | 0.618185913 | 1 | 0.431722212 | 0.646240962 |
| 1081 |  | 0.006 | 0.045 | 0.899 | 1.102012717 | 3 | 0.776588228 | 0.33614861 |
| 1172 |  | -0.003 | 0.050 | 0.951 | 0.95721474 | 3 | 0.811603123 | 0.864012003 |
| 1196 |  | -0.067 | 0.064 | 0.407 | 3.429380315 | 3 | 0.330037417 | 0.282291281 |
| 1267 |  | -0.206 | 0.232 | 0.538 | 1.049327297 | 2 | 0.591754368 | 0.20723674 |

BIDPs=brain imaging-derived phenotypes;MR-Egger=Mendelian randomization Egger regression;MR-PRESSO=Mendelian Randomization Pleiotropy RESidual Sum and Outlier test;SE=standard error;Pval=P value.
